# Supplementary material for: Validation of COI metabarcoding primers for terrestrial arthropods
Source: PeerJ. 2019 Oct 7;7:e7745. doi: 10.7717/peerj.7745 (PMC6786254; doi:10.7717/peerj.7745)
Supplement: Figure S1 [file peerj-07-7745-s001.pdf]

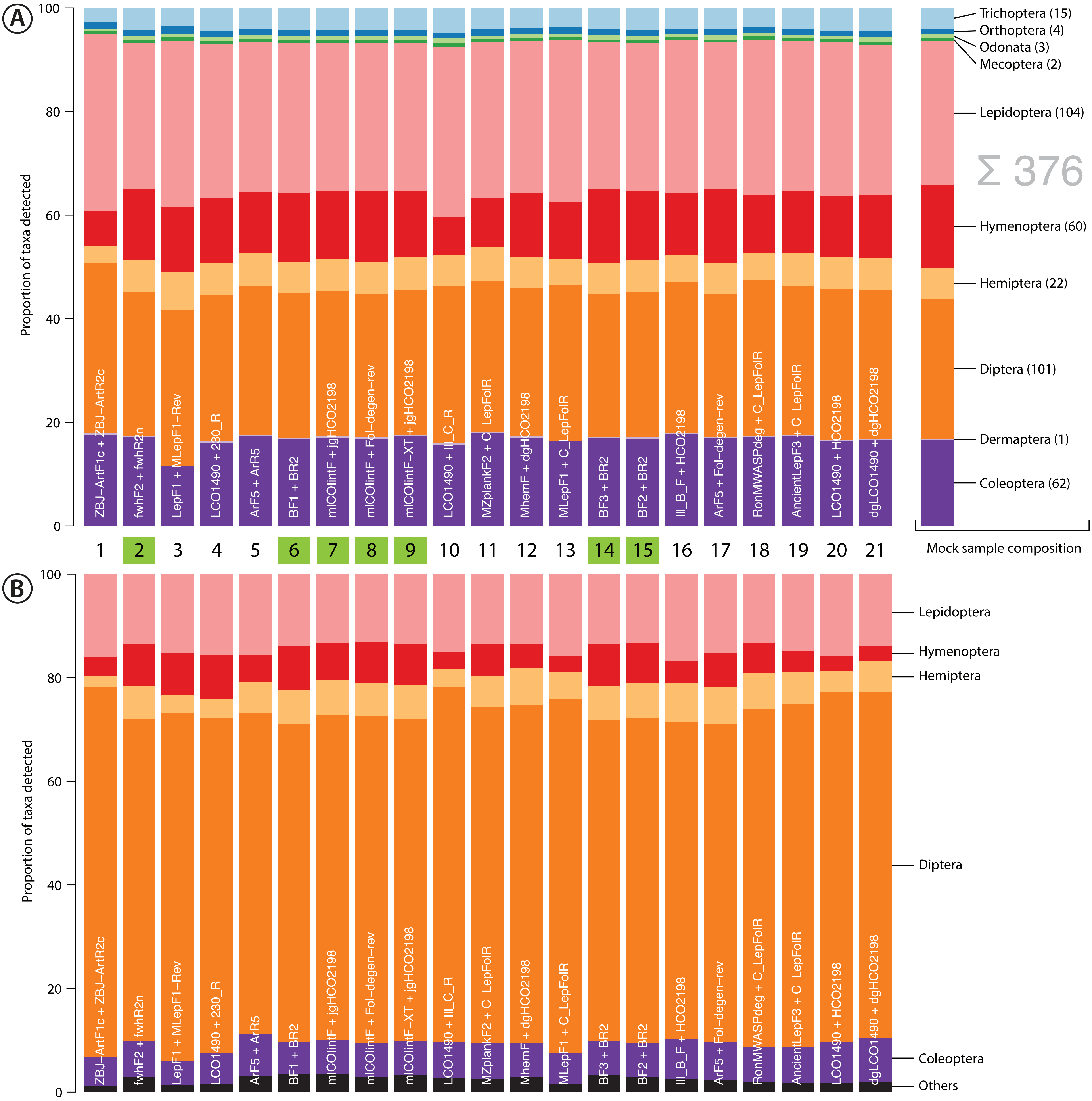


**Figure S1:** Comparison of taxonomic recovery on Order level with 21 primer sets, for the mock community (**A**) and malaise sample (**B**). The mock community composition containing 376 specimens of different orders is shown as well. For a detailed mock community species list see Table S1. Low abundant orders in the malaise trap sample where aggregated into the category "others" and contained species from the orders: Araneae, Entomobryomorpha, Mesostigmata, Neuroptera, Odonata, Psocodea, Sarcoptiformes, Symphypleona, Thysanoptera, Trichoptera and Trombidiformes. IDs of the primer sets with the best taxa recovery in both mock community and malaise sample are highlighted in green (see also Figure 4). Raw read counts are available in Table S1.
