# Supplementary figures and images for: Validation of COI metabarcoding primers for terrestrial arthropods
Source: PeerJ. 2019 Oct 7;7:e7745. doi: 10.7717/peerj.7745 (PMC6786254; doi:10.7717/peerj.7745)

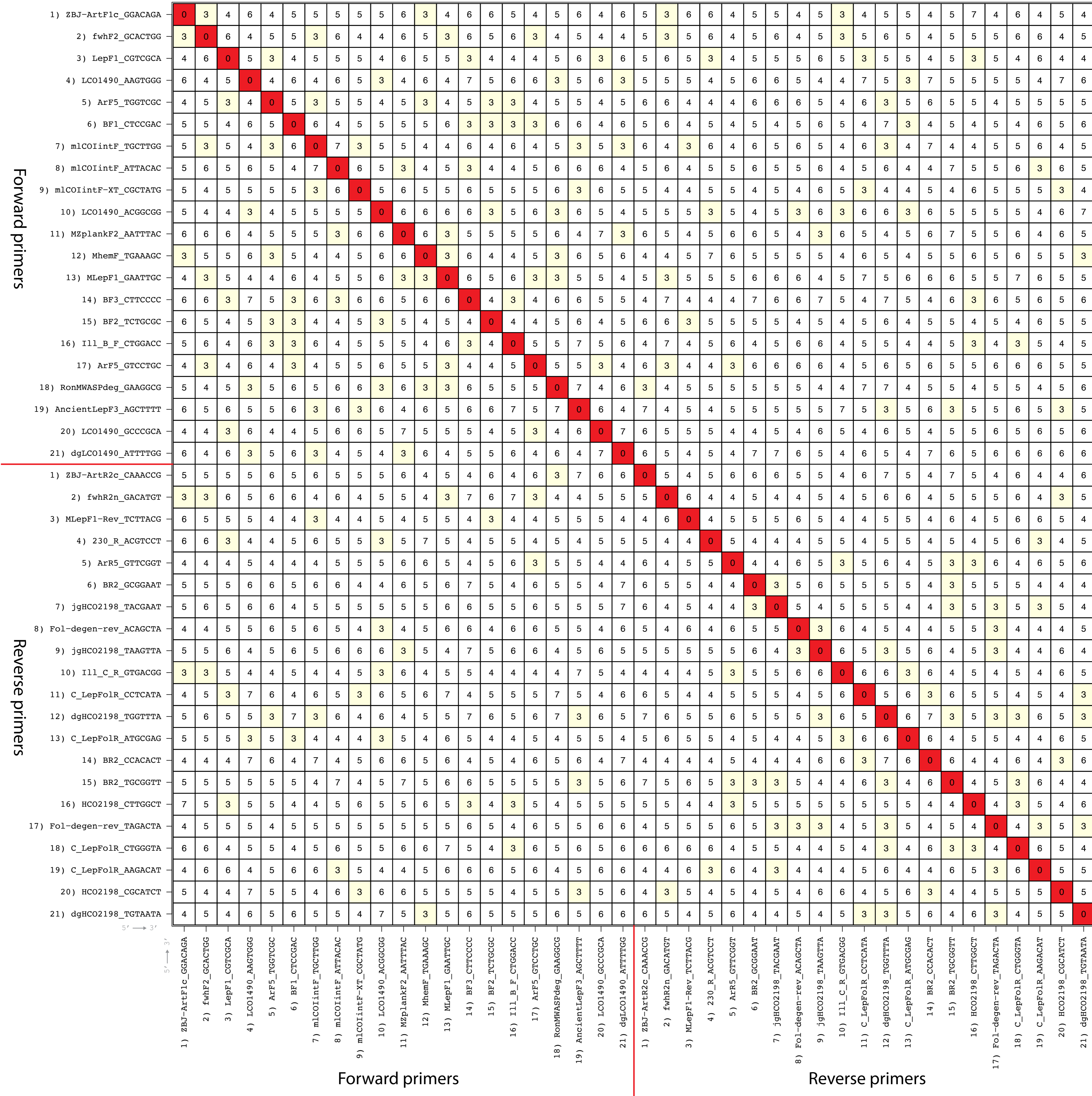

Supplement: Figure S3 [file peerj-07-7745-s003.pdf]

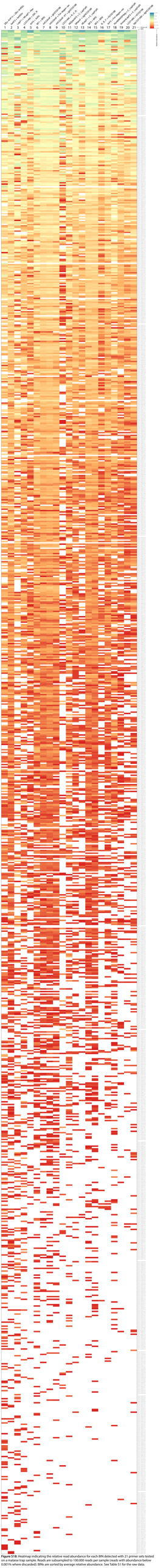

Supplement: Figure S18 [file peerj-07-7745-s018.pdf]

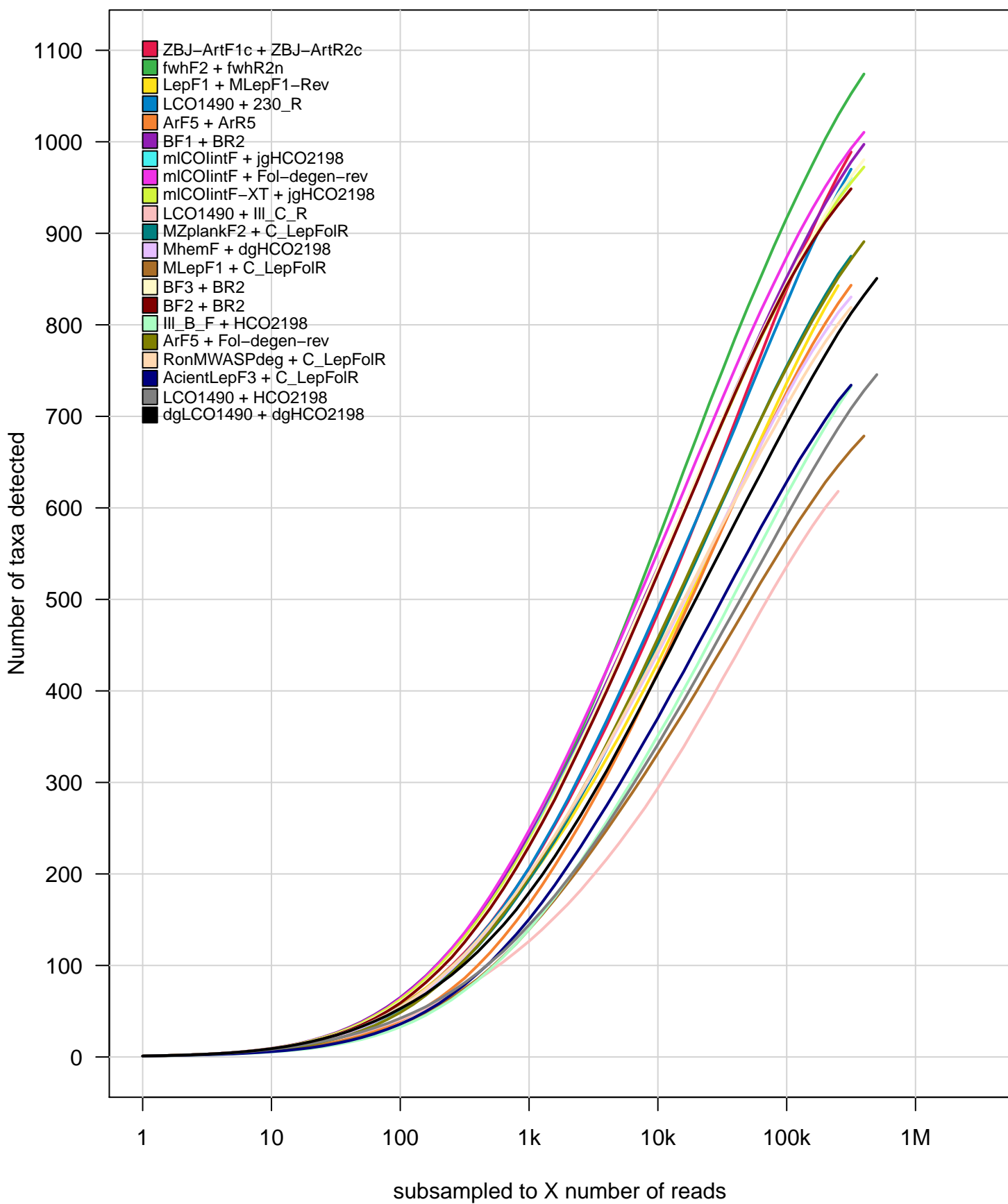

Supplement: Supplemental Information 1 [file peerj-07-7745-s020.zip › Scripts_1_v2/R_scripts/subsampling malaise trap/plot_rare_v2_mBRAVE.pdf]

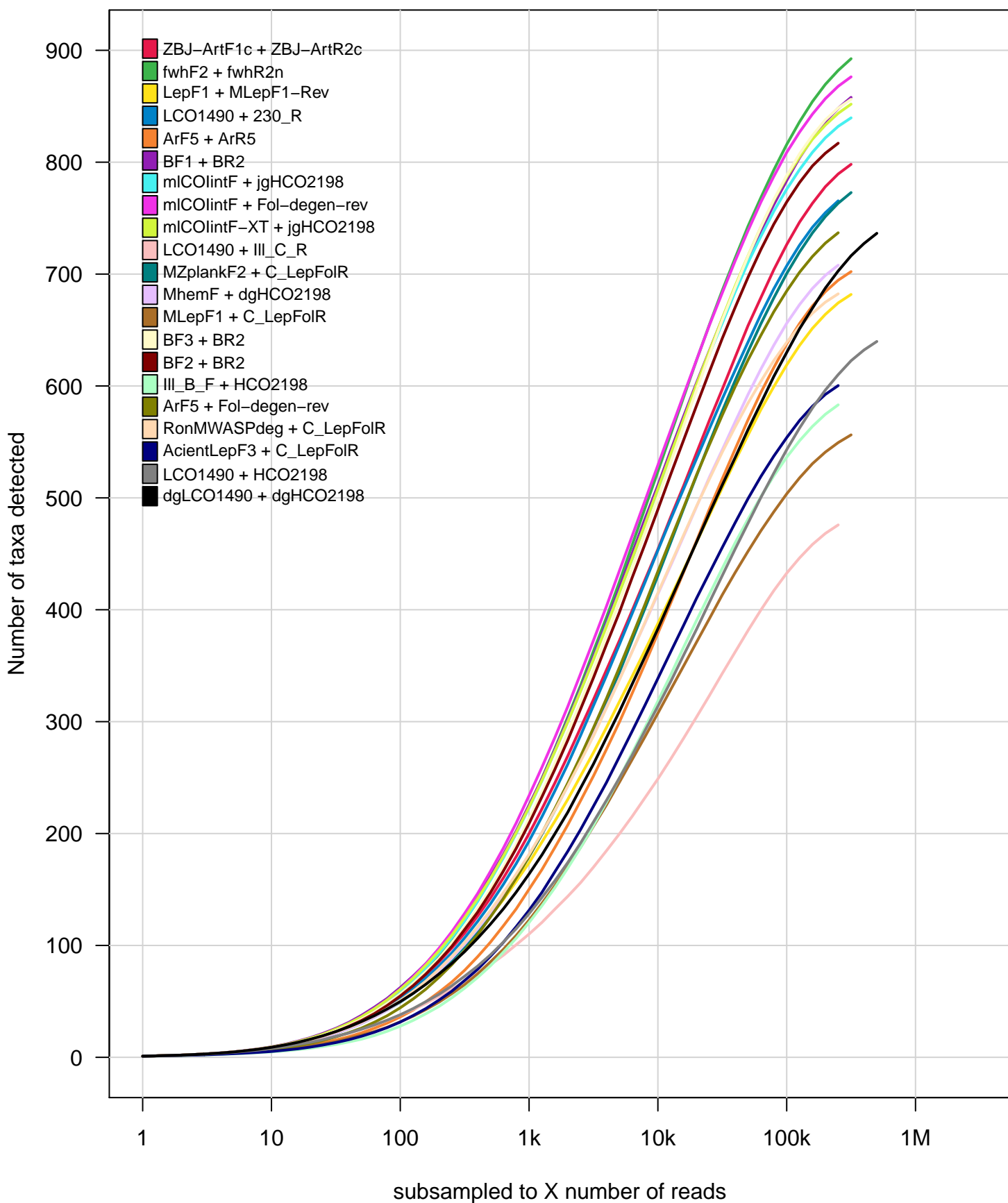

Supplement: Supplemental Information 1 [file peerj-07-7745-s020.zip › Scripts_1_v2/R_scripts/subsampling malaise trap/plot_rare_v2_local.pdf]

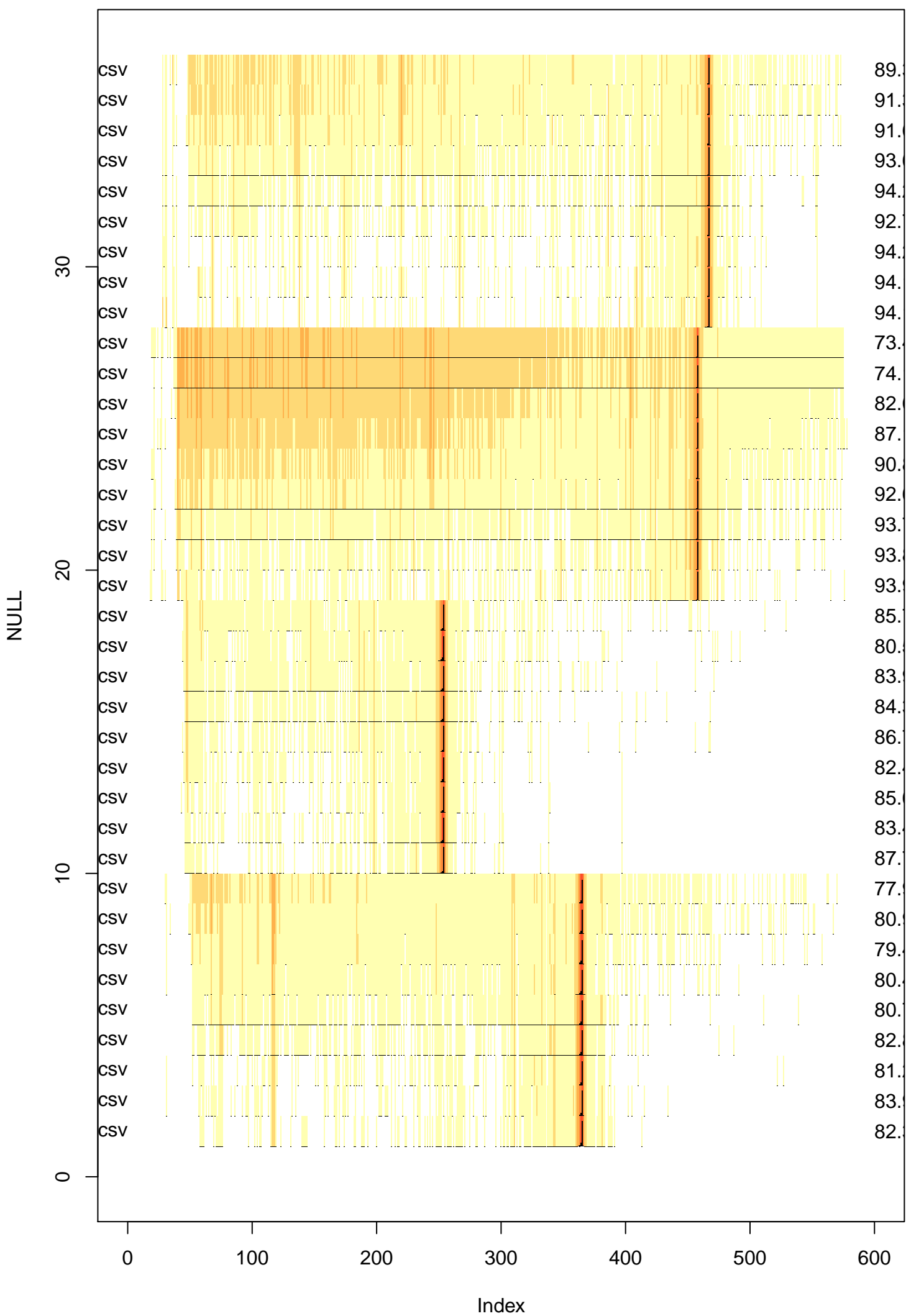

Supplement: Supplemental Information 1 [file peerj-07-7745-s020.zip › Scripts_1_v2/R_scripts/unspecific_amplification/180912_gradient.pdf]

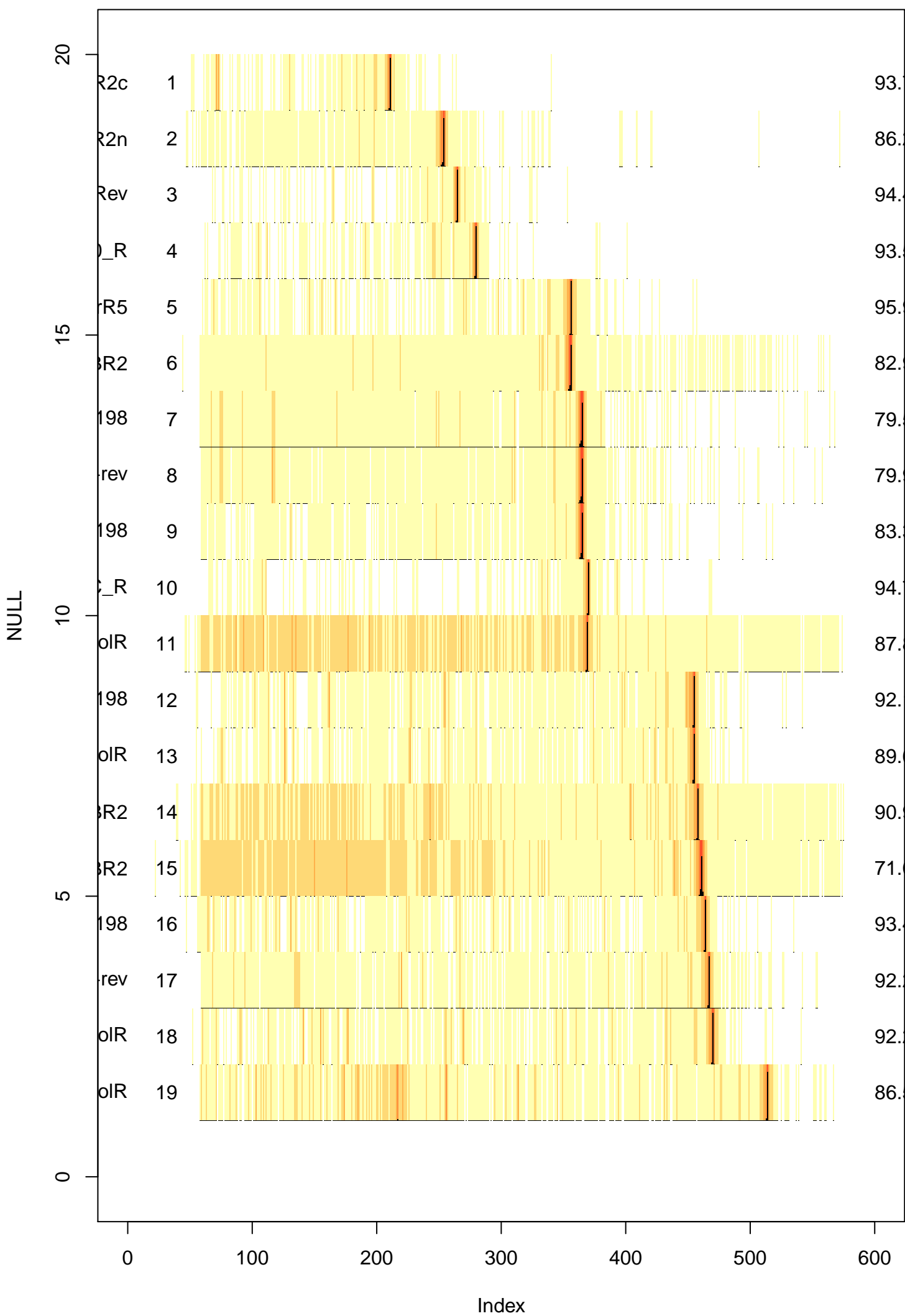

Supplement: Supplemental Information 1 [file peerj-07-7745-s020.zip › Scripts_1_v2/R_scripts/unspecific_amplification/21primer.pdf]

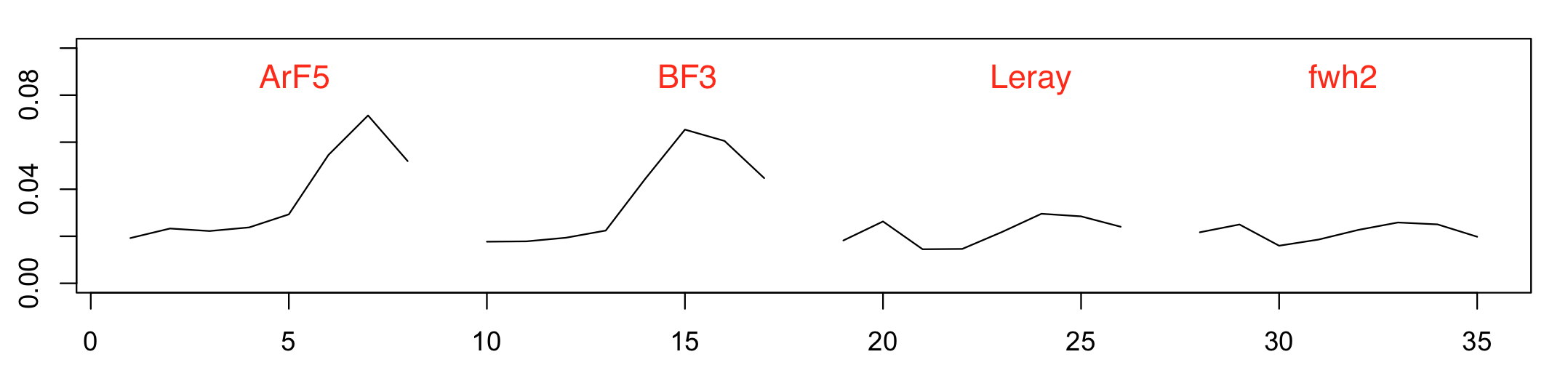

Supplement: Supplemental Information 1 [file peerj-07-7745-s020.zip › Scripts_1_v2/R_scripts/Fig SXXX SXXX SXX gradient differences/Screen Shot 2018-08-18 at 10.42.09.png]

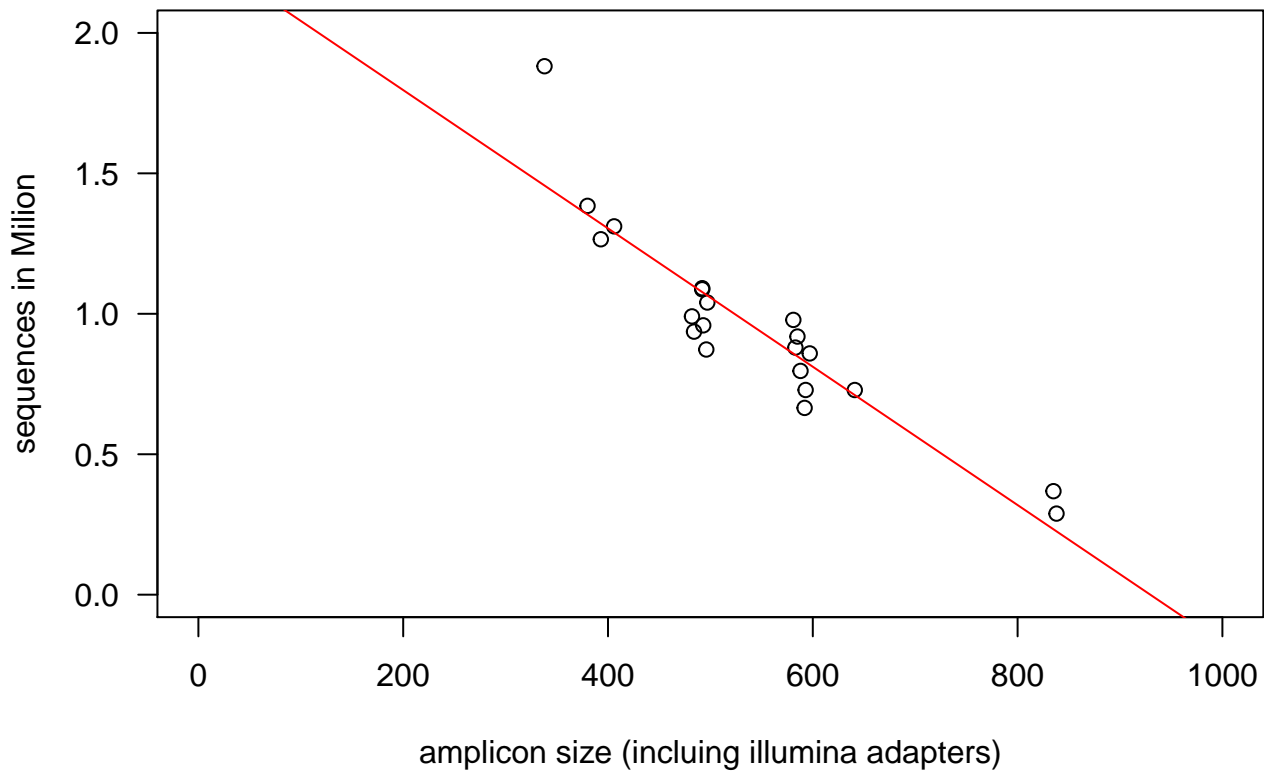

Supplement: Supplemental Information 1 [file peerj-07-7745-s020.zip › Scripts_1_v2/R_scripts/sequencing depth/abline_v4.pdf]

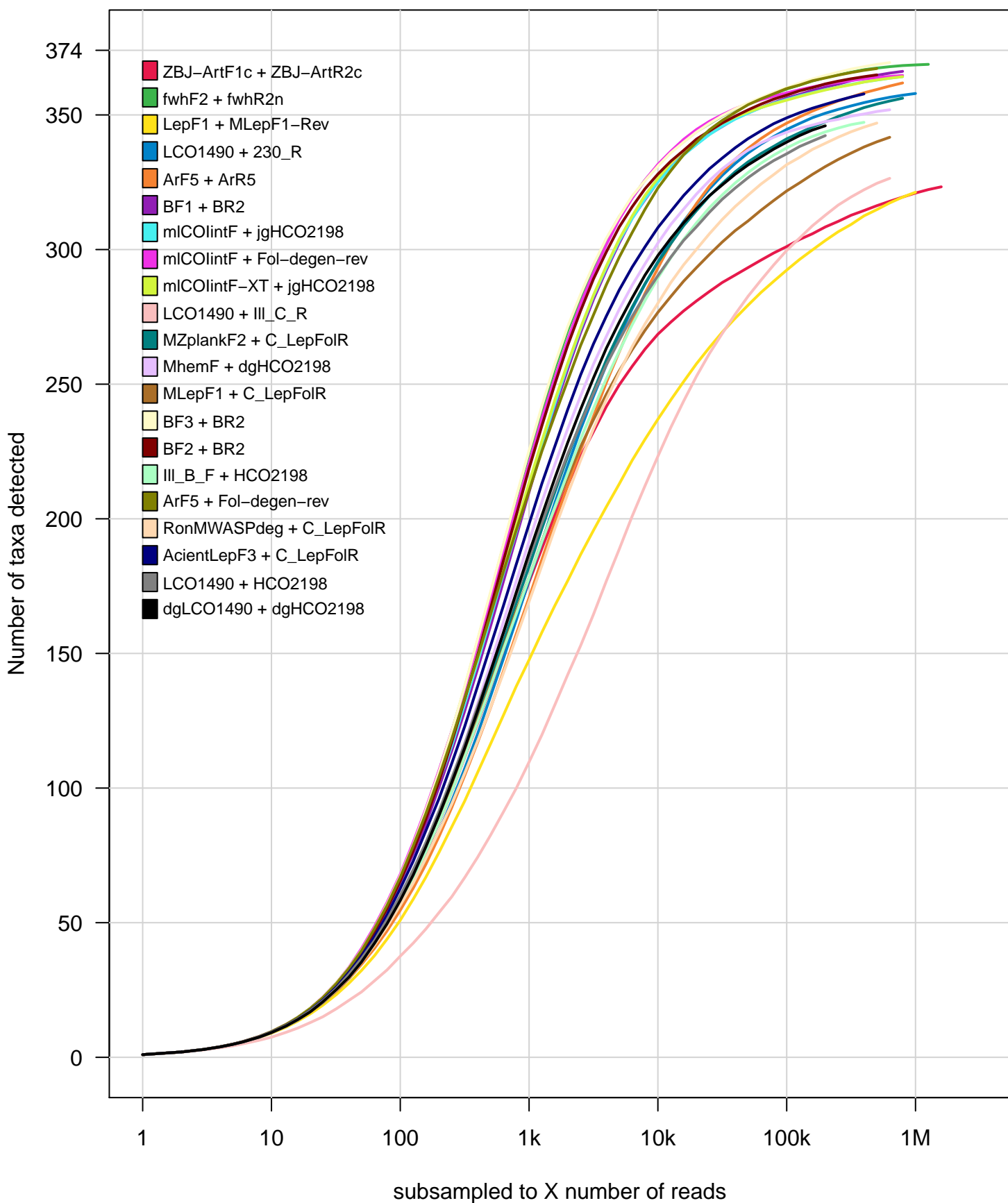

Supplement: Supplemental Information 1 [file peerj-07-7745-s020.zip › Scripts_1_v2/R_scripts/subsampling mock community/plot_rare_v2.pdf]

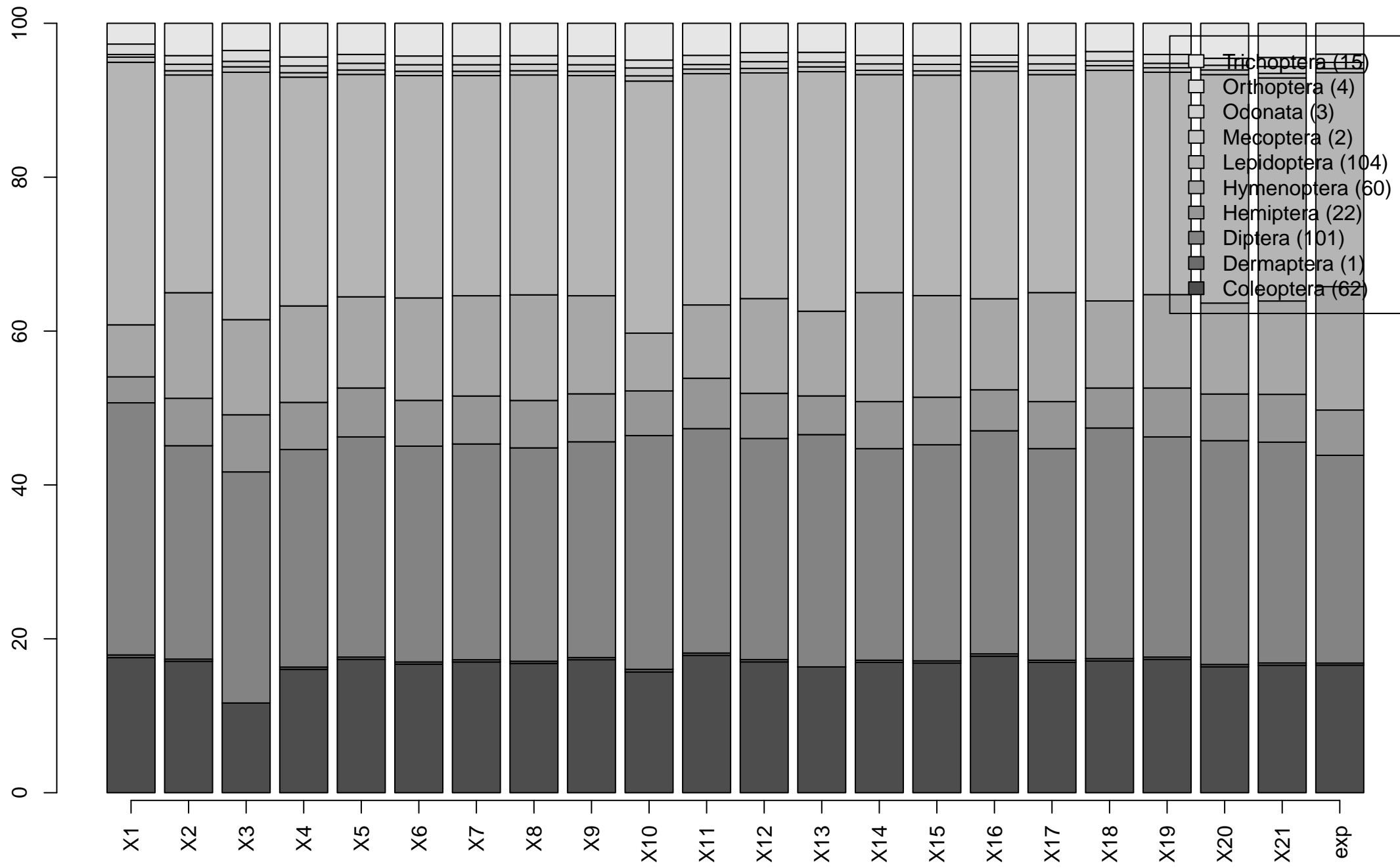

Supplement: Supplemental Information 1 [file peerj-07-7745-s020.zip › Scripts_1_v2/R_scripts/taxonomic groups/Mock_plot_old.pdf]

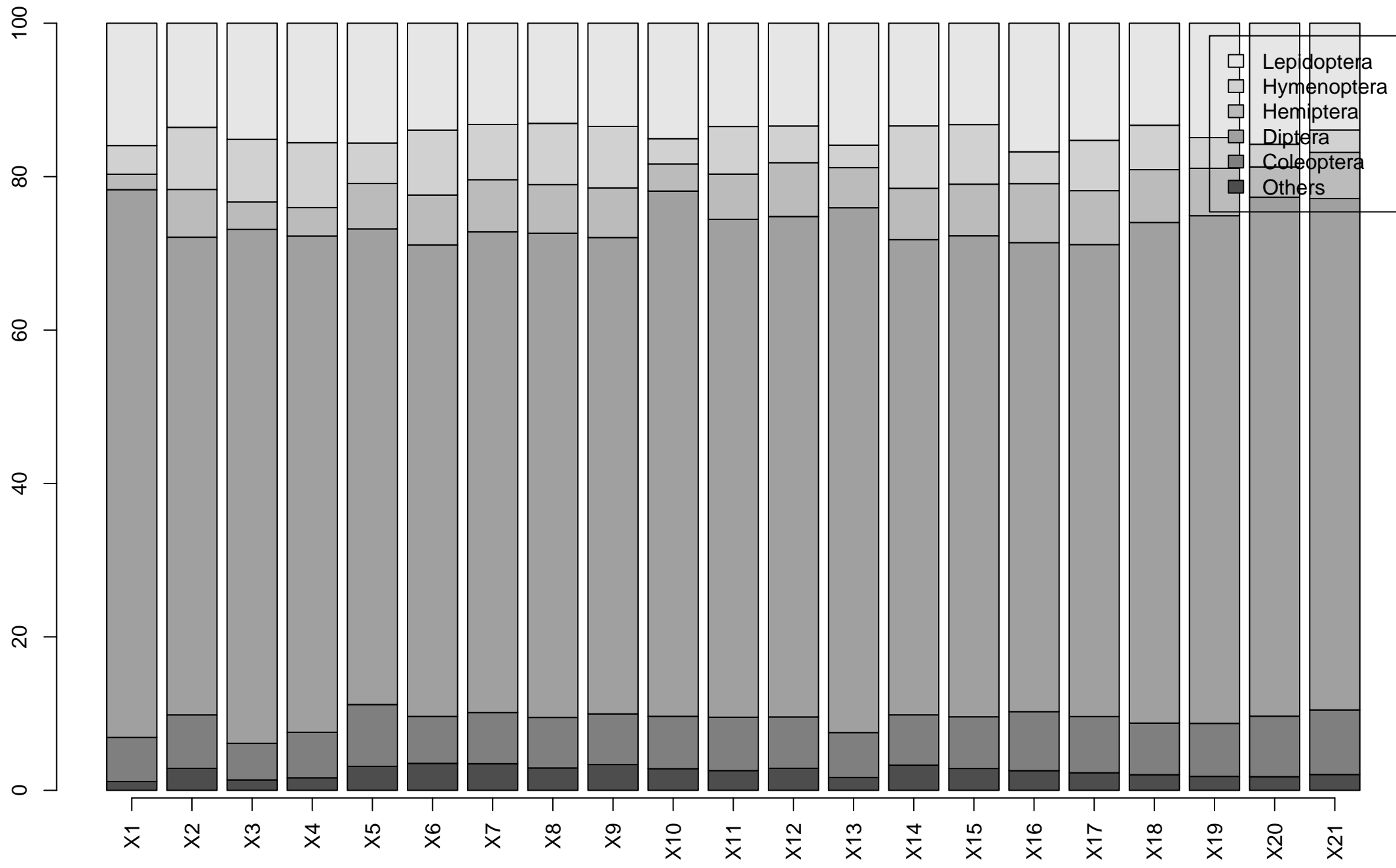

Supplement: Supplemental Information 1 [file peerj-07-7745-s020.zip › Scripts_1_v2/R_scripts/taxonomic groups/malaise_plot.pdf]

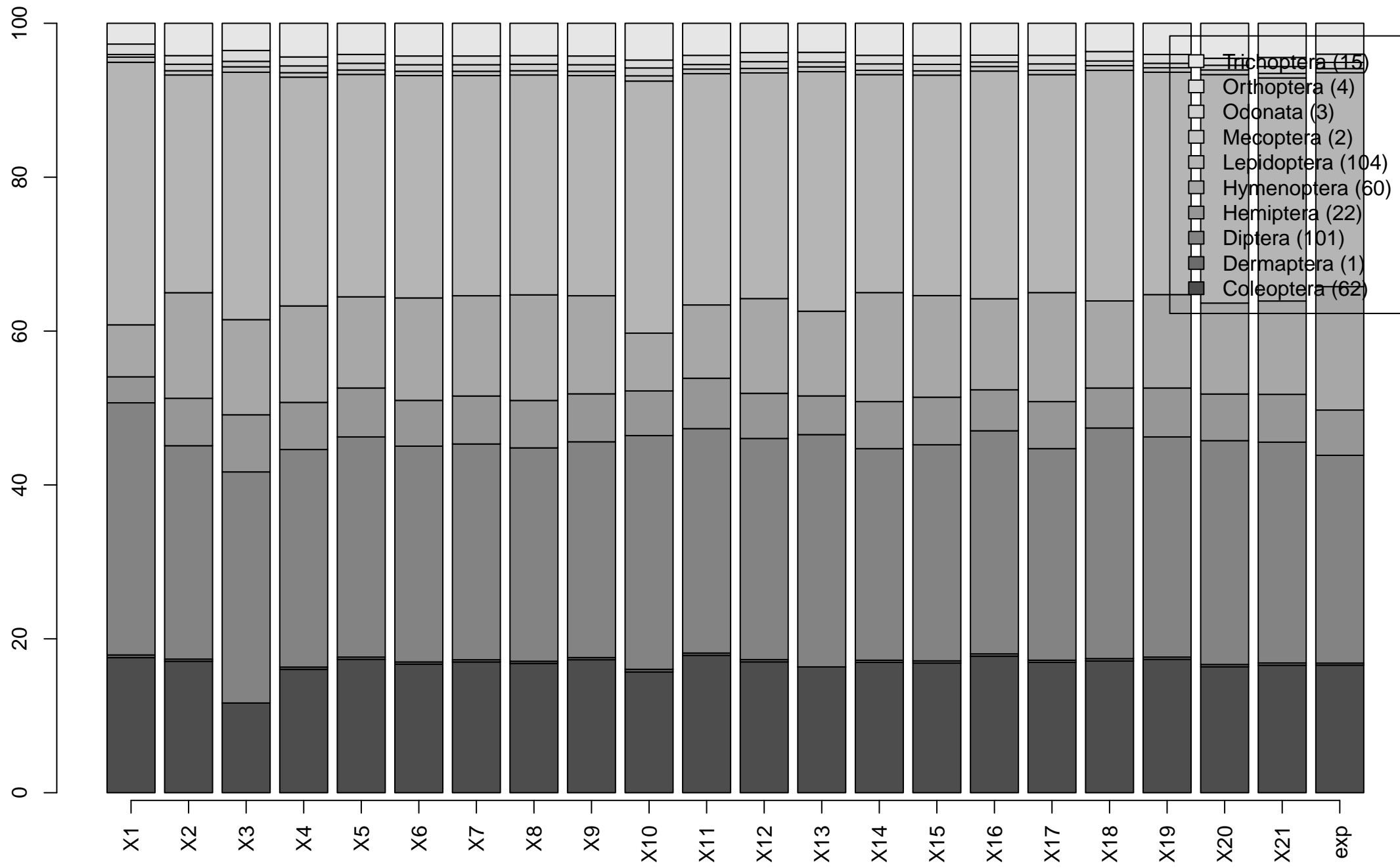

Supplement: Supplemental Information 1 [file peerj-07-7745-s020.zip › Scripts_1_v2/R_scripts/taxonomic groups/Mock_plot.pdf]

due to low read quality

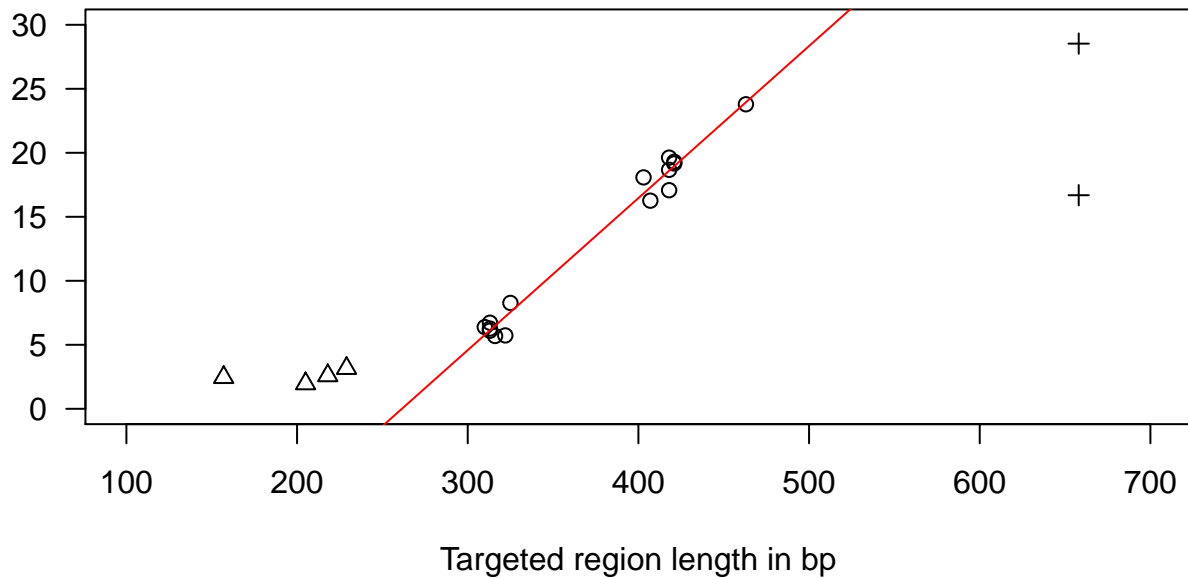

Supplement: Supplemental Information 1 [file peerj-07-7745-s020.zip › Scripts_1_v2/R_scripts/sequ loss (filtering)/ee_filtering.pdf]

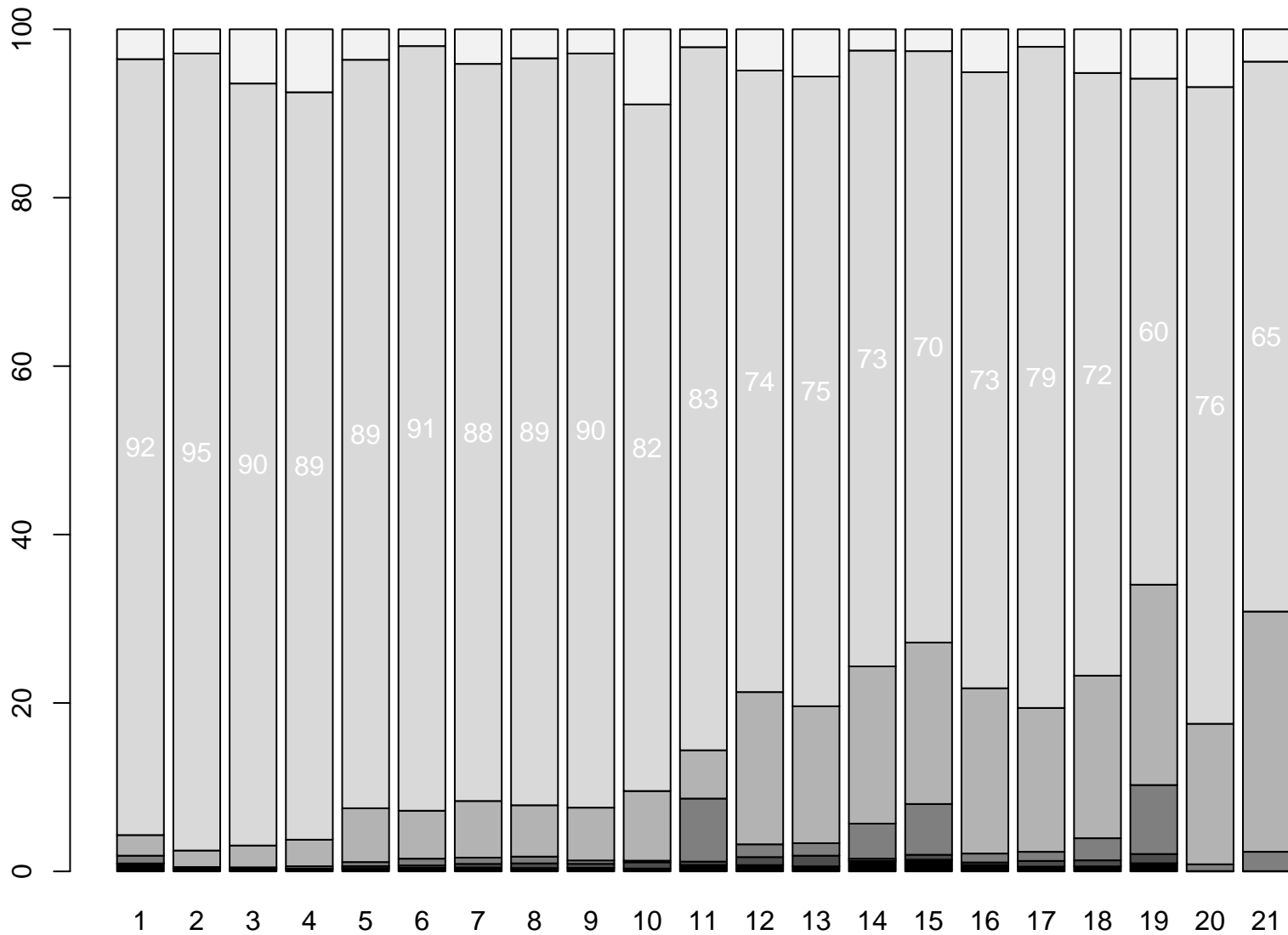

Supplement: Supplemental Information 1 [file peerj-07-7745-s020.zip › Scripts_1_v2/R_scripts/sequ loss (filtering)/sequ_loss.pdf]
