# Supplementary material for: Validation of COI metabarcoding primers for terrestrial arthropods
Source: PeerJ. 2019 Oct 7;7:e7745. doi: 10.7717/peerj.7745 (PMC6786254; doi:10.7717/peerj.7745)
Supplement: Figure S4 [file peerj-07-7745-s004.pdf]

Figure S4: Fusion primers for testing 21 primer sets.

P5 1) ZBJ-ArtF1c	AATGATACGGCGACCACCGAGATCTACACTCTTTCCCTACACGACGCTCTTCCGATCTGGACAGATATTGGAACWTTATATTTTATTTTTGG
P7 2) fwhF2	CAAGCAGAAGACGGCATACGAGATGTGACTGGAGTTCAGACGTGTGCTCTTCCGATCTGCACTGGDACWGGWTGAACWGTWTAYCCHCC
P5 3) LepF1	AATGATACGGCGACCACCGAGATCTACACTCTTTCCCTACACGACGCTCTTCCGATCTCGTCGCAATTCAACCAATCATAAAGATATTGG
P7 4) LCO1490	CAAGCAGAAGACGGCATACGAGATGTGACTGGAGTTCAGACGTGTGCTCTTCCGATCTAAGTGGGTCAACAAATCATAAAGATATTGG
P5 5) ArF5	AATGATACGGCGACCACCGAGATCTACACTCTTTCCCTACACGACGCTCTTCCGATCTTGGTCGCICCIGAYATRKCITTYCCICG
P7 6) BF1	CAAGCAGAAGACGGCATACGAGATGTGACTGGAGTTCAGACGTGTGCTCTTCCGATCTCTCCGACWGGWTGRACWGTNTAYCC
P5 7) mlCOIintF	AATGATACGGCGACCACCGAGATCTACACTCTTTCCCTACACGACGCTCTTCCGATCTTGCTTGGWACWGGWTGAACWGTWTAYCCYCC
P7 8) mlCOIintF	CAAGCAGAAGACGGCATACGAGATGTGACTGGAGTTCAGACGTGTGCTCTTCCGATCTATTACACGGWACWGGWTGAACWGTWTAYCCYCC
P5 9) mlCOIintF-XT	AATGATACGGCGACCACCGAGATCTACACTCTTTCCCTACACGACGCTCTTCCGATCTCGCTATGGWACWRGWTGRACWITITAYCCYCC
P7 10) LCO1490	CAAGCAGAAGACGGCATACGAGATGTGACTGGAGTTCAGACGTGTGCTCTTCCGATCTACGGCGGTCAACAAATCATAAAGATATTGG
P5 11) MZplankF2	AATGATACGGCGACCACCGAGATCTACACTCTTTCCCTACACGACGCTCTTCCGATCTAATTTACRGYNGGNACRGGNTGRACNGT
P7 12) MhemF	CAAGCAGAAGACGGCATACGAGATGTGACTGGAGTTCAGACGTGTGCTCTTCCGATCTTGAAAGCATTYCCACGAATAAATAAYATAAG
P5 13) MLepF1	AATGATACGGCGACCACCGAGATCTACACTCTTTCCCTACACGACGCTCTTCCGATCTGAATTGCTTTCCCACGAATAAATAATA
P7 14) BF3	CAAGCAGAAGACGGCATACGAGATGTGACTGGAGTTCAGACGTGTGCTCTTCCGATCTCTTCCCCHGAYATRGCHTTYCCHCG
P5 15) BF2	AATGATACGGCGACCACCGAGATCTACACTCTTTCCCTACACGACGCTCTTCCGATCTTCTGCGCHCCHGAYATRGCHTTYCC
P7 16) Ill_B_F	CAAGCAGAAGACGGCATACGAGATGTGACTGGAGTTCAGACGTGTGCTCTTCCGATCTCTGGACCCIGAYATRGCITTYCCICG
P5 17) ArF5	AATGATACGGCGACCACCGAGATCTACACTCTTTCCCTACACGACGCTCTTCCGATCTGTCCTGCICCIGAYATRKCITTYCCICG
P7 18) RonMWASPdeg	CAAGCAGAAGACGGCATACGAGATGTGACTGGAGTTCAGACGTGTGCTCTTCCGATCTGAAGGCGGWTCWCCWGATATAKCWTTTCC
P5 19) AncientLepF3	AATGATACGGCGACCACCGAGATCTACACTCTTTCCCTACACGACGCTCTTCCGATCTAGCTTTTATAATTGGDGGWTTTGGWAATTG
P7 20) LCO1490	CAAGCAGAAGACGGCATACGAGATGTGACTGGAGTTCAGACGTGTGCTCTTCCGATCTGCCCGCAGGTCAACAAATCATAAAGATATTGG
P5 21) dgLCO1490	AATGATACGGCGACCACCGAGATCTACACTCTTTCCCTACACGACGCTCTTCCGATCTATTTTGGTCAACAAATCATAAAGAYATYGG

P7 1) ZBJ-ArtR2c	CAAGCAGAAGACGGCATACGAGATGTGACTGGAGTTCAGACGTGTGCTCTTCCGATCTCAAACCGWACTAATCAATTWCCAAATCCTCC
P5 2) fwhR2n	AATGATACGGCGACCACCGAGATCTACACTCTTTCCCTACACGACGCTCTTCCGATCTGACATGTRATWGCHCCDGCTARWACWGG
P7 3) MLepF1-Rev	CAAGCAGAAGACGGCATACGAGATGTGACTGGAGTTCAGACGTGTGCTCTTCCGATCTTCTTACGTGGAAAWGCTATATCWGGTG
P5 4) 230_R	AATGATACGGCGACCACCGAGATCTACACTCTTTCCCTACACGACGCTCTTCCGATCTACGTCCTTATRTTRTTTATICGIGGRAAIGC
P7 5) ArR5	CAAGCAGAAGACGGCATACGAGATGTGACTGGAGTTCAGACGTGTGCTCTTCCGATCTGTTCGGTRATIGCICCIGCIARIACIGG
P5 6) BR2	AATGATACGGCGACCACCGAGATCTACACTCTTTCCCTACACGACGCTCTTCCGATCTGCGGAATTCDGGRTGNCCRAARAAYCA
P7 7) jgHCO2198	CAAGCAGAAGACGGCATACGAGATGTGACTGGAGTTCAGACGTGTGCTCTTCCGATCTTACGAATAIACYTCIGGRTGICCRAARAAYCA
P5 8) Fol-degen-rev	AATGATACGGCGACCACCGAGATCTACACTCTTTCCCTACACGACGCTCTTCCGATCTACAGCTANACYTCNGGRTGNCCRAARAAYCA
P7 9) jgHCO2198	CAAGCAGAAGACGGCATACGAGATGTGACTGGAGTTCAGACGTGTGCTCTTCCGATCTTAAGTTAIACYTCIGGRTGICCRAARAAYCA
P5 10) Ill_C_R	AATGATACGGCGACCACCGAGATCTACACTCTTTCCCTACACGACGCTCTTCCGATCTGTGACGGIGGRTAIACIGTTCAICC
P7 11) C_LepFolR	CAAGCAGAAGACGGCATACGAGATGTGACTGGAGTTCAGACGTGTGCTCTTCCGATCTCCTCATAAACTTCWGGRTGWCCAAAAAATCA
P5 12) dgHCO2198	AATGATACGGCGACCACCGAGATCTACACTCTTTCCCTACACGACGCTCTTCCGATCTTGGTTTAAACTTCAGGGTGACCAAARAAYCA
P7 13) C_LepFolR	CAAGCAGAAGACGGCATACGAGATGTGACTGGAGTTCAGACGTGTGCTCTTCCGATCTATGCGAGTAAACTTCWGGRTGWCCAAAAAATCA
P5 14) BR2	AATGATACGGCGACCACCGAGATCTACACTCTTTCCCTACACGACGCTCTTCCGATCTCCACACTCDGGRTGNCCRAARAAYCA
P7 15) BR2	CAAGCAGAAGACGGCATACGAGATGTGACTGGAGTTCAGACGTGTGCTCTTCCGATCTTGCGGTTCDGGRTGNCCRAARAAYCA
P5 16) HCO2198	AATGATACGGCGACCACCGAGATCTACACTCTTTCCCTACACGACGCTCTTCCGATCTCTTGGCTAAACTTCAGGGTGACCAAAAAATCA
P7 17) Fol-degen-rev	CAAGCAGAAGACGGCATACGAGATGTGACTGGAGTTCAGACGTGTGCTCTTCCGATCTTAGACTANACYTCNGGRTGNCCRAARAAYCA
P5 18) C_LepFolR	AATGATACGGCGACCACCGAGATCTACACTCTTTCCCTACACGACGCTCTTCCGATCTCTGGGTAAACTTCWGGRTGWCCAAAAAATCA
P7 19) C_LepFolR	CAAGCAGAAGACGGCATACGAGATGTGACTGGAGTTCAGACGTGTGCTCTTCCGATCTAAGACATAAACTTCWGGRTGWCCAAAAAATCA
P5 20) HCO2198	AATGATACGGCGACCACCGAGATCTACACTCTTTCCCTACACGACGCTCTTCCGATCTCGCATCTAAACTTCAGGGTGACCAAAAAATCA
P7 21) dgHCO2198	CAAGCAGAAGACGGCATACGAGATGTGACTGGAGTTCAGACGTGTGCTCTTCCGATCTTGTAATAAACTTCAGGGTGACCAAARAAYCA

Legend: P5 / P7 Illumina adapter (incl. flow cell & sequencing primer bind)
In-line tag & heterogeneity spacer
Forward / Reverse primer
