# Supplementary material for: Validation of COI metabarcoding primers for terrestrial arthropods
Source: PeerJ. 2019 Oct 7;7:e7745. doi: 10.7717/peerj.7745 (PMC6786254; doi:10.7717/peerj.7745)
Supplement: Figure S5 [file peerj-07-7745-s005.pdf]

Figure S5: Fusion primers for gradient metabarcoding.

P7 2) fwhF2	CAAGCAGAAGACGGCATACGAGATGTGACTGGAGTTCAGACGTGTGCTCTTCCGATCT	GCACT	GGDACWGGWTGAACWGTWTAYCCHCC
P7 2B) fwhF2	CAAGCAGAAGACGGCATACGAGATGTGACTGGAGTTCAGACGTGTGCTCTTCCGATCT	TGATGG	GGDACWGGWTGAACWGTWTAYCCHCC
P7 2C) fwhF2	CAAGCAGAAGACGGCATACGAGATGTGACTGGAGTTCAGACGTGTGCTCTTCCGATCT	CGGG	GGDACWGGWTGAACWGTWTAYCCHCC
P7 8) mlCOIintF	CAAGCAGAAGACGGCATACGAGATGTGACTGGAGTTCAGACGTGTGCTCTTCCGATCT	ATTACAC	GGWACWGGWTGAACWGTWTAYCCYCC
P7 8B) mlCOIintF	CAAGCAGAAGACGGCATACGAGATGTGACTGGAGTTCAGACGTGTGCTCTTCCGATCT	GCGTAT	GGWACWGGWTGAACWGTWTAYCCYCC
P7 8C) mlCOIintF	CAAGCAGAAGACGGCATACGAGATGTGACTGGAGTTCAGACGTGTGCTCTTCCGATCT	ATTTA	GGWACWGGWTGAACWGTWTAYCCYCC
P5 14) BF3	AATGATACGGCGACCACCGAGATCTACACTCTTTCCCTACACGACGCTCTTCCGATCT	CTTCC	CCHGAYATRGCHTTYCCHCG
P5 14B) BF3	AATGATACGGCGACCACCGAGATCTACACTCTTTCCCTACACGACGCTCTTCCGATCT	AACG	CCHGAYATRGCHTTYCCHCG
P5 14C) BF3	AATGATACGGCGACCACCGAGATCTACACTCTTTCCCTACACGACGCTCTTCCGATCT	GCAAAC	CCHGAYATRGCHTTYCCHCG
P5 17n) ArF5	AATGATACGGCGACCACCGAGATCTACACTCTTTCCCTACACGACGCTCTTCCGATCT	GTCCT	GC**N**CC**N**GAYATRKC**N**TTYCC**N**CG
P5 17Bn) ArF5	AATGATACGGCGACCACCGAGATCTACACTCTTTCCCTACACGACGCTCTTCCGATCT	ATTGGAA	GC**N**CC**N**GAYATRKC**N**TTYCC**N**CG
P5 17Cn) ArF5	AATGATACGGCGACCACCGAGATCTACACTCTTTCCCTACACGACGCTCTTCCGATCT	CAATAA	GC**N**CC**N**GAYATRKC**N**TTYCC**N**CG
P5 2) fwhR2n	AATGATACGGCGACCACCGAGATCTACACTCTTTCCCTACACGACGCTCTTCCGATCT	GACAT	GTRATWGCHCCDGCTARWACWGG
P5 2B) fwhR2n	AATGATACGGCGACCACCGAGATCTACACTCTTTCCCTACACGACGCTCTTCCGATCT	GATTCC	GTRATWGCHCCDGCTARWACWGG
P5 2C) fwhR2n	AATGATACGGCGACCACCGAGATCTACACTCTTTCCCTACACGACGCTCTTCCGATCT	CGCC	GTRATWGCHCCDGCTARWACWGG
P5 8) Fol-degen-rev	AATGATACGGCGACCACCGAGATCTACACTCTTTCCCTACACGACGCTCTTCCGATCT	ACAGC	TANACYTCNGGRTGNCCRAARAAYCA
P5 8A) Fol-degen-rev	AATGATACGGCGACCACCGAGATCTACACTCTTTCCCTACACGACGCTCTTCCGATCT	TCGC	TANACYTCNGGRTGNCCRAARAAYCA
P5 8B) Fol-degen-rev	AATGATACGGCGACCACCGAGATCTACACTCTTTCCCTACACGACGCTCTTCCGATCT	AGGTGC	TANACYTCNGGRTGNCCRAARAAYCA
P7 **15**) BR2	CAAGCAGAAGACGGCATACGAGATGTGACTGGAGTTCAGACGTGTGCTCTTCCGATCT	TGCGGT	TCDGGRTGNCCRAARAAYCA
P7 14A) BR2	CAAGCAGAAGACGGCATACGAGATGTGACTGGAGTTCAGACGTGTGCTCTTCCGATCT	CTCCA	TCDGGRTGNCCRAARAAYCA
P7 14B) BR2	CAAGCAGAAGACGGCATACGAGATGTGACTGGAGTTCAGACGTGTGCTCTTCCGATCT	CGGA	TCDGGRTGNCCRAARAAYCA
P7 17) Fol-degen-rev	CAAGCAGAAGACGGCATACGAGATGTGACTGGAGTTCAGACGTGTGCTCTTCCGATCT	TAGAC	TANACYTCNGGRTGNCCRAARAAYCA
P7 17A) Fol-degen-rev	CAAGCAGAAGACGGCATACGAGATGTGACTGGAGTTCAGACGTGTGCTCTTCCGATCT	TCAGAA	TANACYTCNGGRTGNCCRAARAAYCA
P7 17B) Fol-degen-rev	CAAGCAGAAGACGGCATACGAGATGTGACTGGAGTTCAGACGTGTGCTCTTCCGATCT	TATA	TANACYTCNGGRTGNCCRAARAAYCA

Legend: P5 / P7 Illumina adapter (incl. flow cell & sequencing primer bind)
In-line tag & heterogeneity spacer
Forward / Reverse primer
