# Supplementary material for: Validation of COI metabarcoding primers for terrestrial arthropods
Source: PeerJ. 2019 Oct 7;7:e7745. doi: 10.7717/peerj.7745 (PMC6786254; doi:10.7717/peerj.7745)
Supplement: Figure S7 [file peerj-07-7745-s007.pdf]

Figure S7: Gel pictures for gradients of all 36 tested primer sets. Primer pairs are ordered by size of the targeted region, as in Figure 2. The temperatures 44.3 °C and 44.7 °C were used as positive and negative controls. A 1% agarose gel was and GeneRuler 1 kb Plus DNA Ladder used in each picture. The expected amplicon size in base pair is annotated on the first visible band of each primer pair.

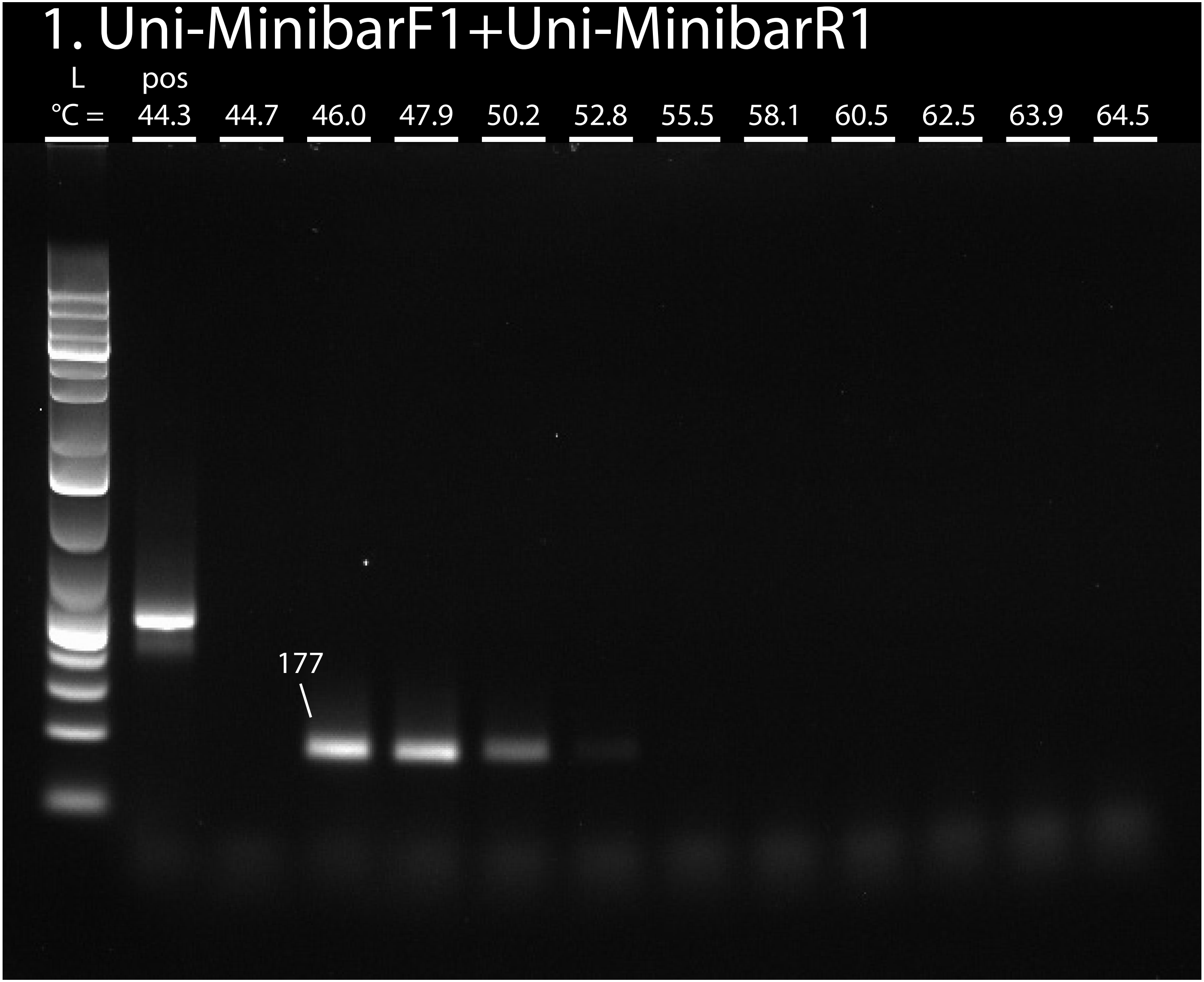
1. Uni-MinibarF1+Uni-MinibarR1
L
pos
°C =
44.3
44.7
46.0
47.9
50.2
52.8
55.5
58.1
60.5
62.5
63.9
64.5
177

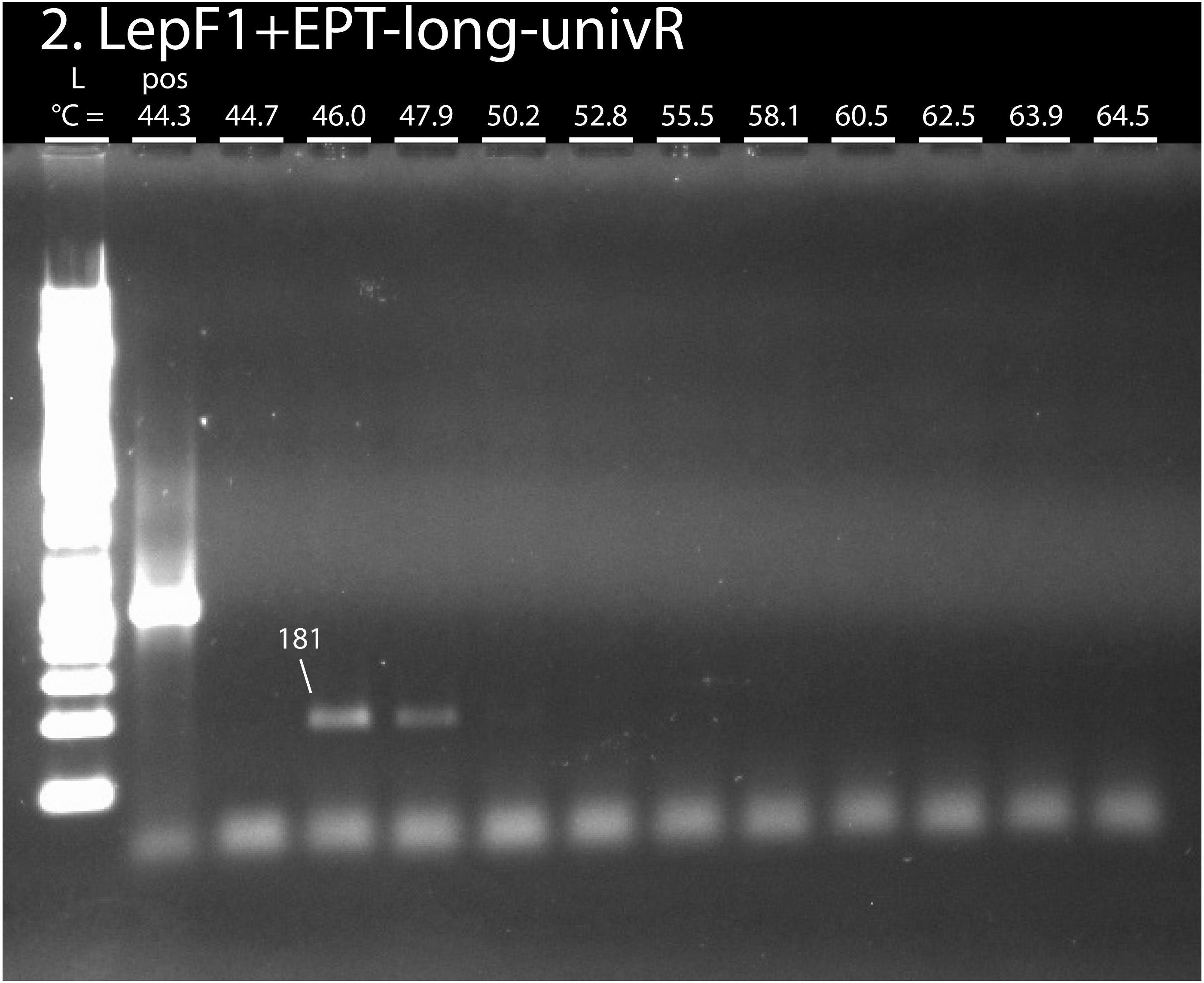
2. LepF1+EPT-long-univR
L
pos
°C =
44.3
44.7
46.0
47.9
50.2
52.8
55.5
58.1
60.5
62.5
63.9
64.5
181

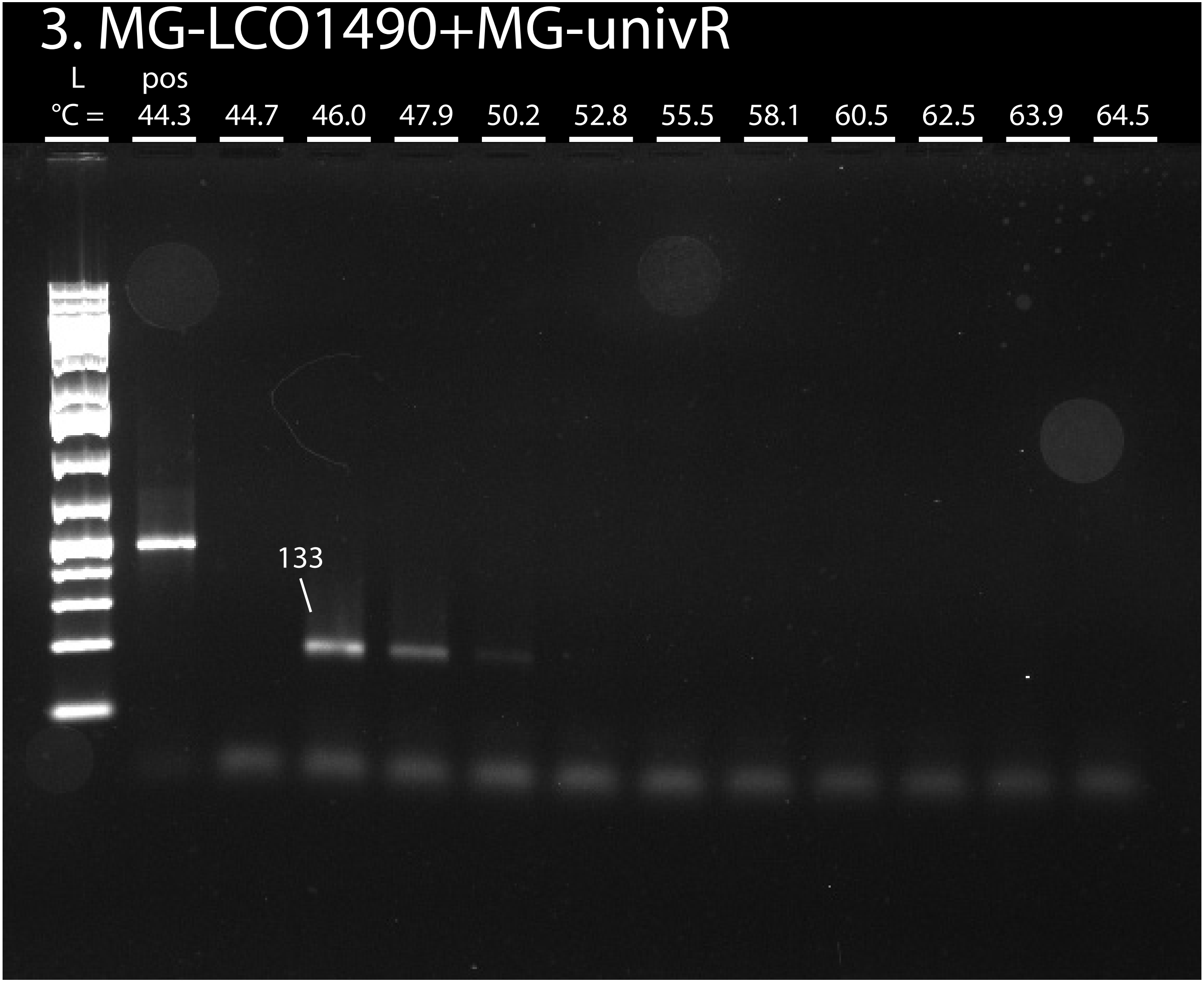
3. MG-LCO1490+MG-univR
L
pos
°C =
44.3
44.7
46.0
47.9
50.2
52.8
55.5
58.1
60.5
62.5
63.9
64.5
133

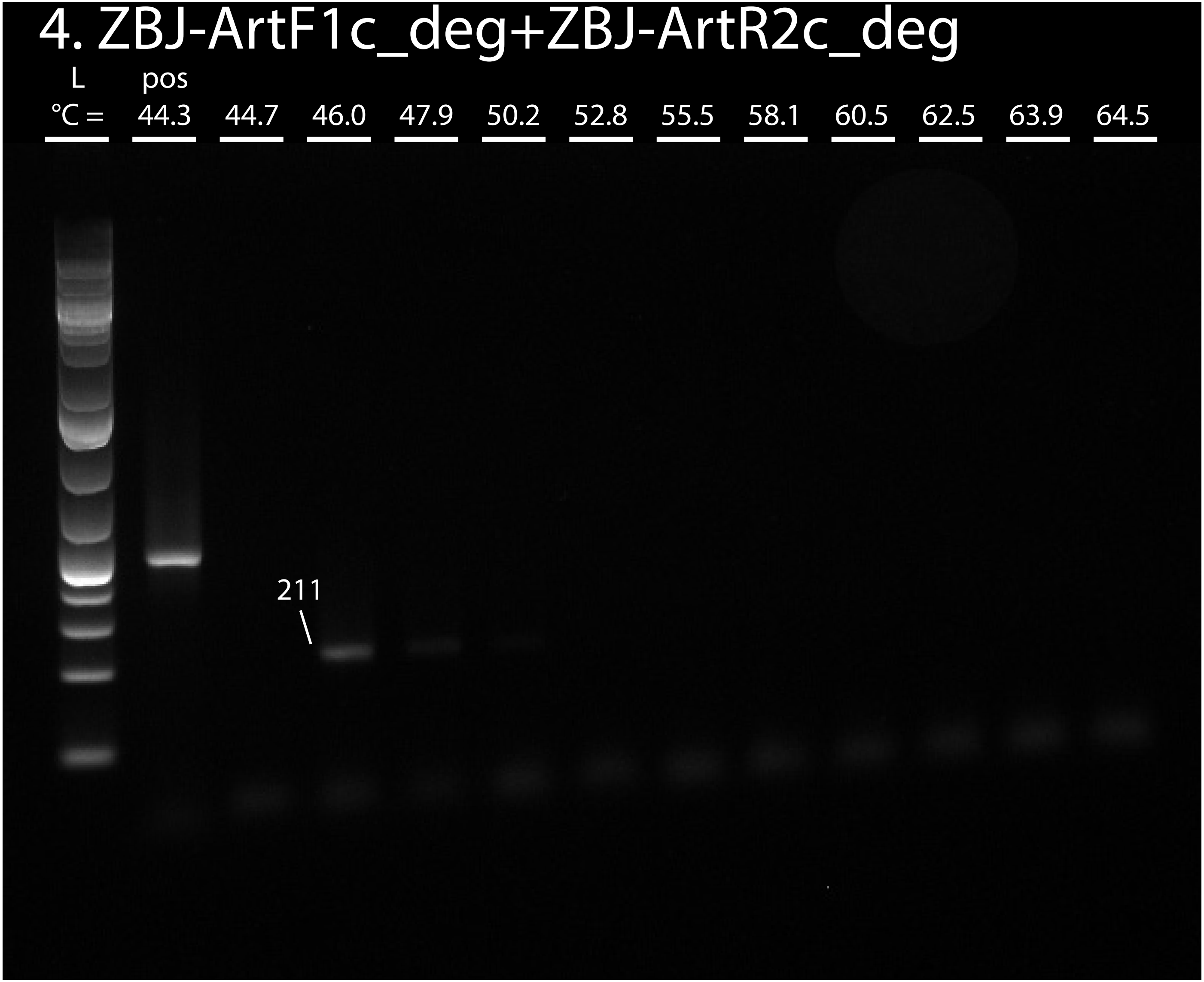
4. ZBJ-ArtF1c_deg+ZBJ-ArtR2c_deg
L
pos
°C =
44.3
44.7
46.0
47.9
50.2
52.8
55.5
58.1
60.5
62.5
63.9
64.5
211

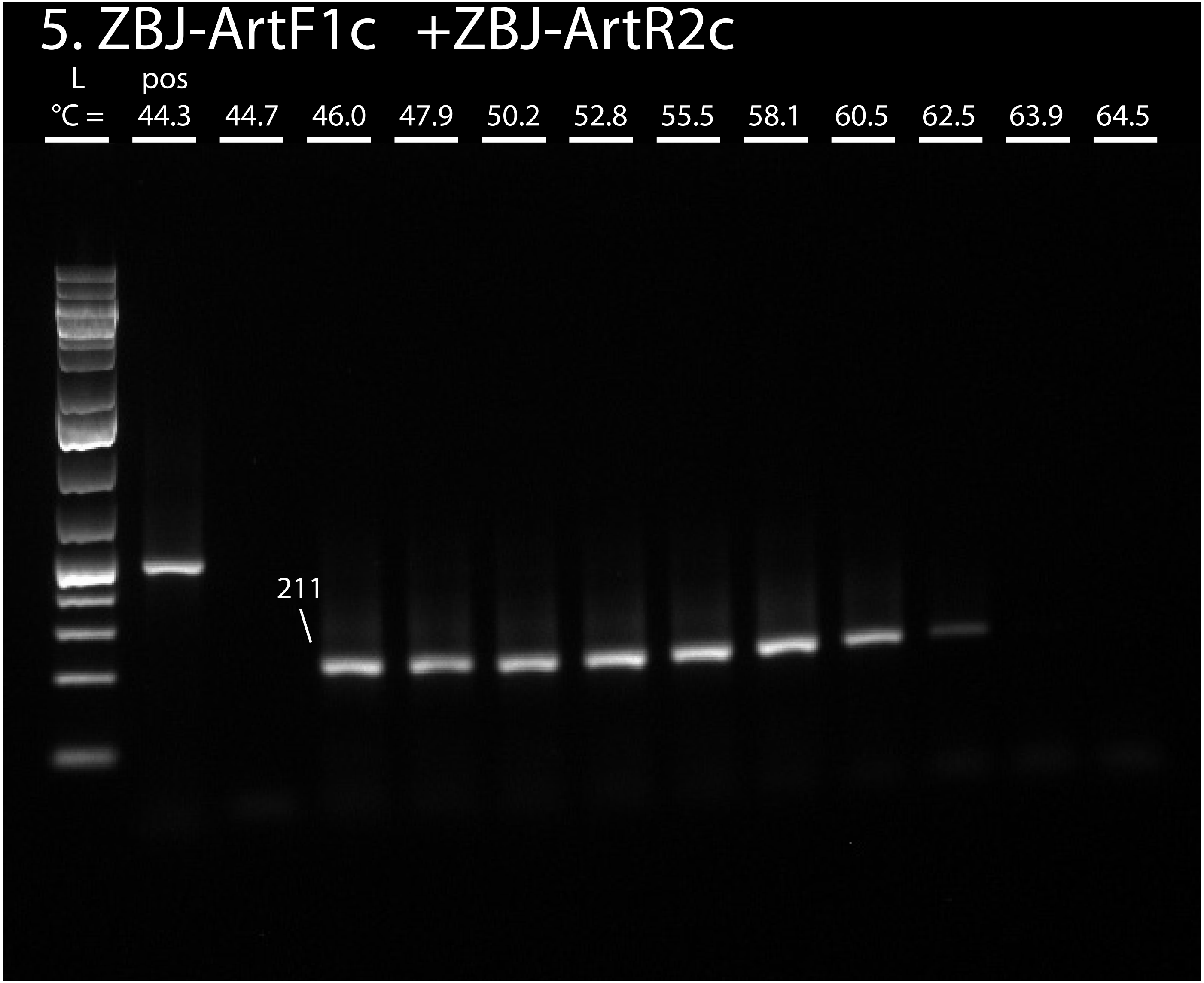
5. ZBJ-ArtF1c +ZBJ-ArtR2c
L
pos
°C =
44.3
44.7
46.0
47.9
50.2
52.8
55.5
58.1
60.5
62.5
63.9
64.5
211

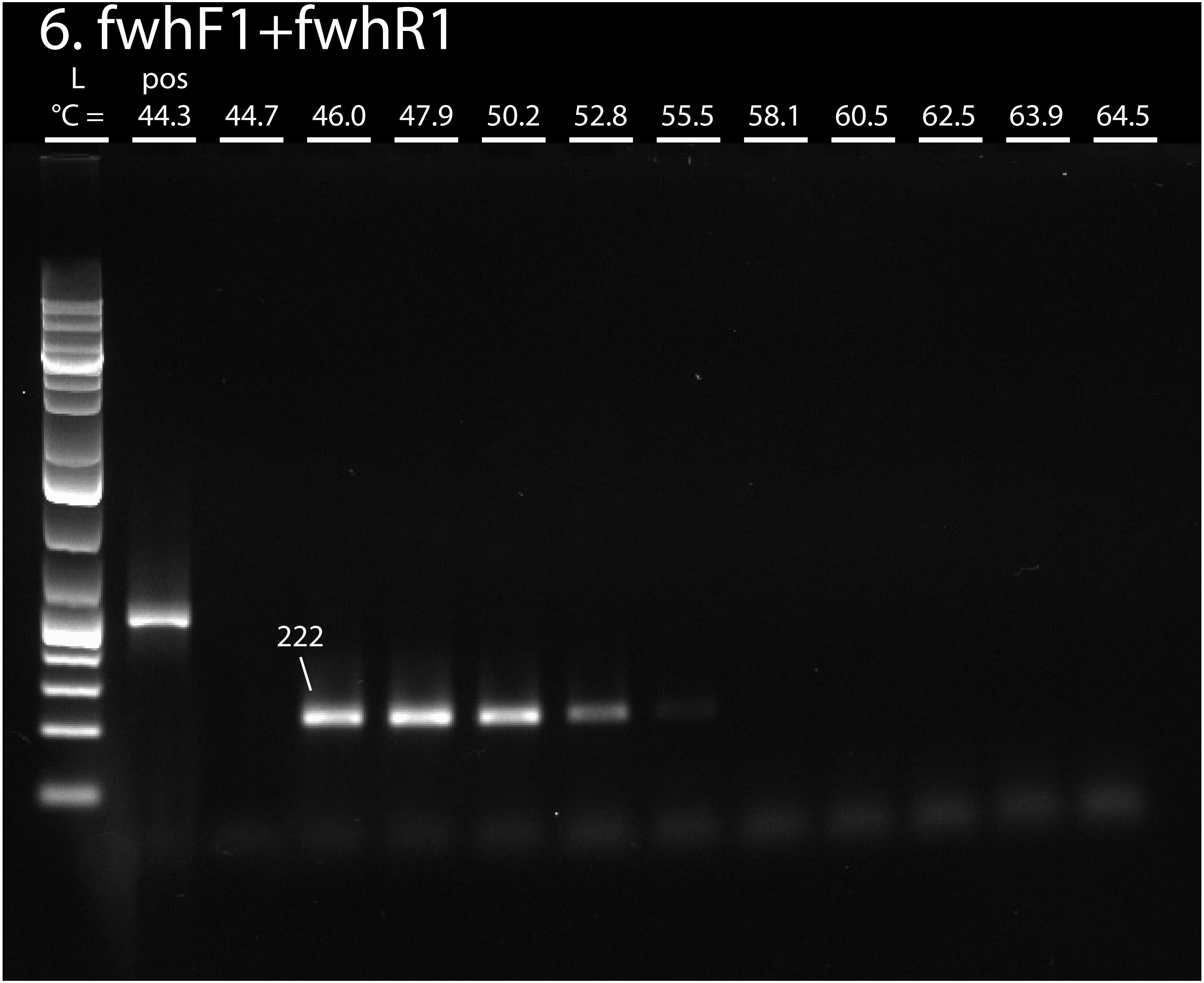
6. fwhF1+fwhR1
L
pos
°C =
44.3
44.7
46.0
47.9
50.2
52.8
55.5
58.1
60.5
62.5
63.9
64.5
222

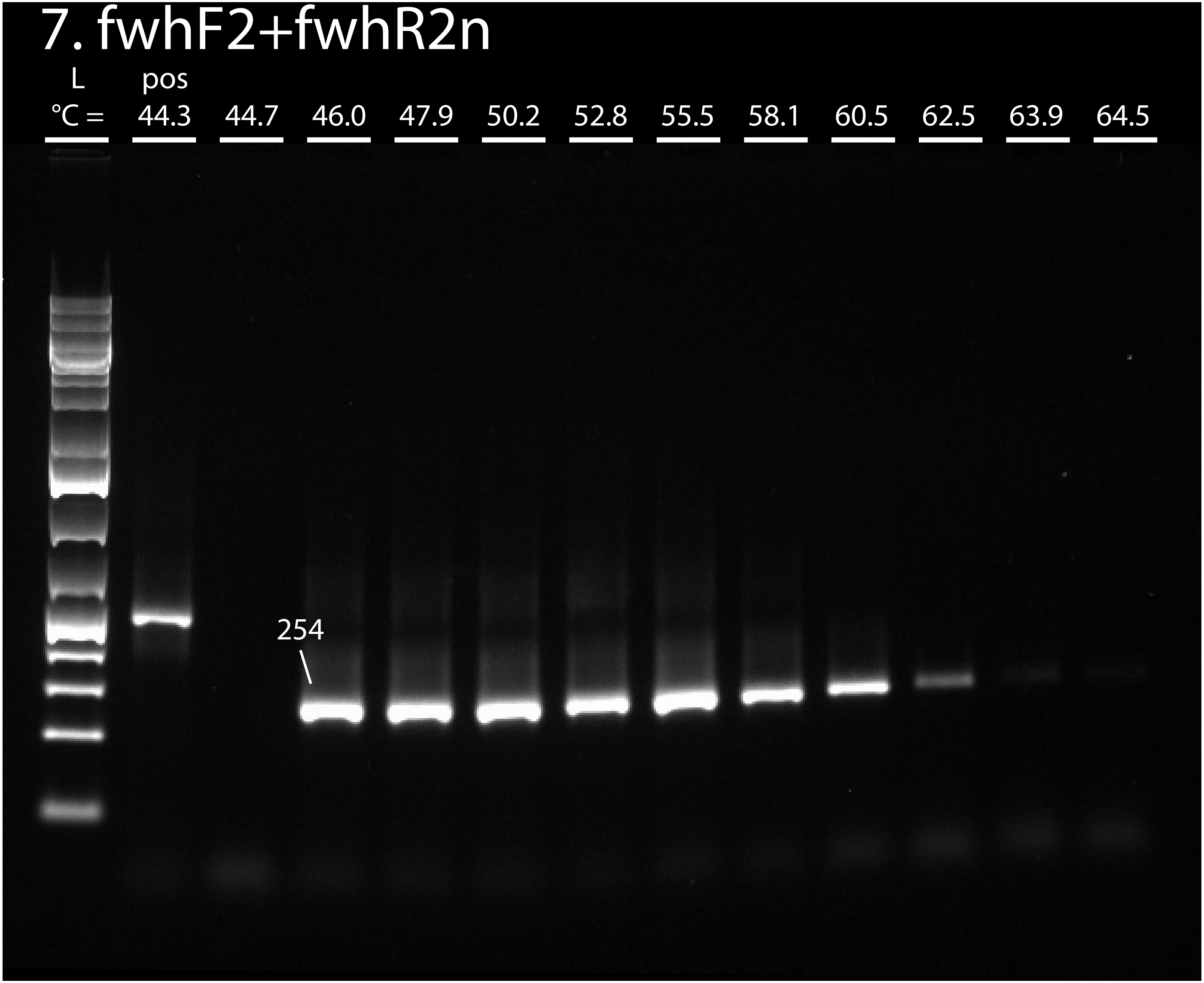
7. fwhF2+fwhR2n
L
pos
°C =
44.3
44.7
46.0
47.9
50.2
52.8
55.5
58.1
60.5
62.5
63.9
64.5
254

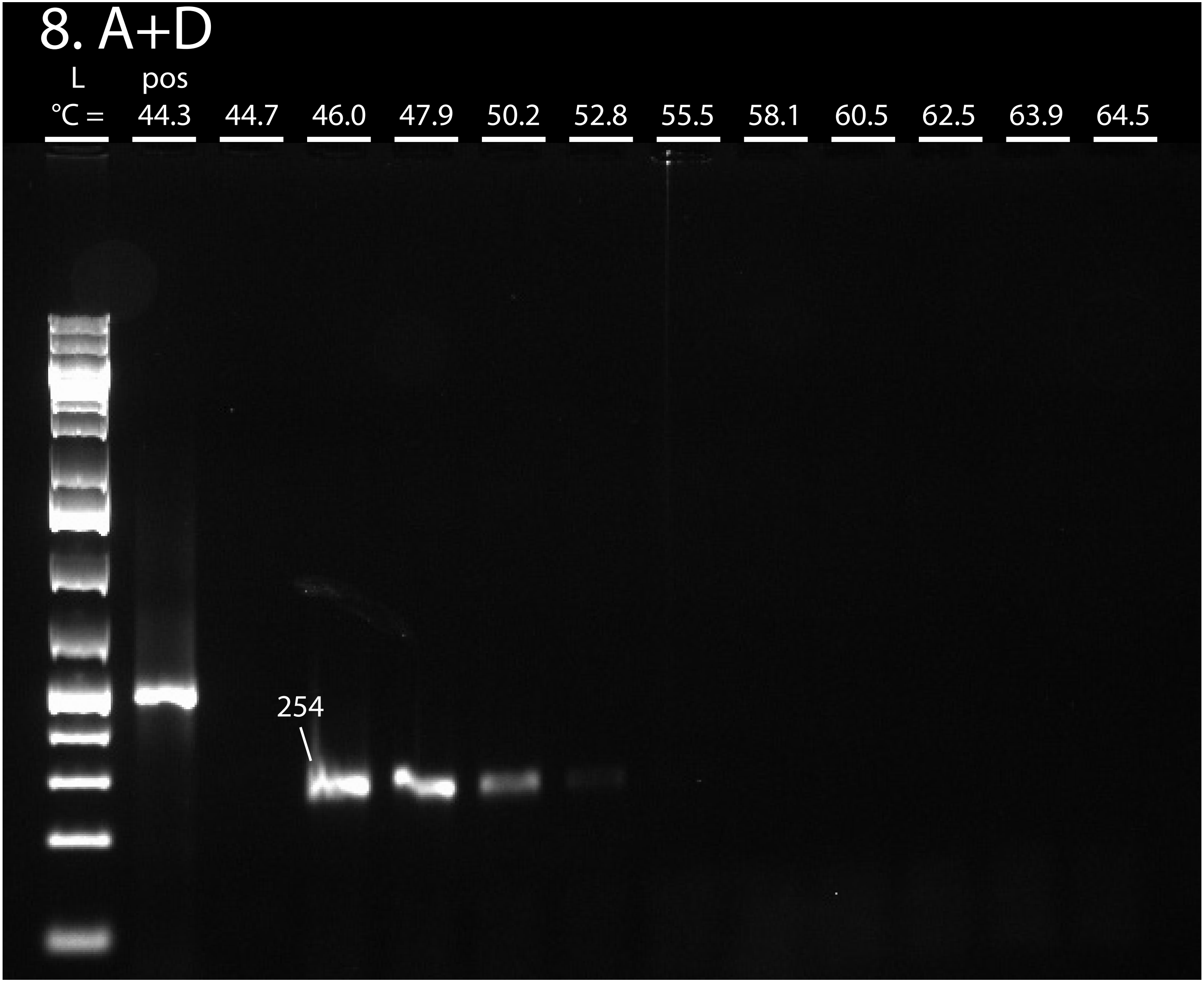
8. A+D
L
pos
°C =
44.3
44.7
46.0
47.9
50.2
52.8
55.5
58.1
60.5
62.5
63.9
64.5
254

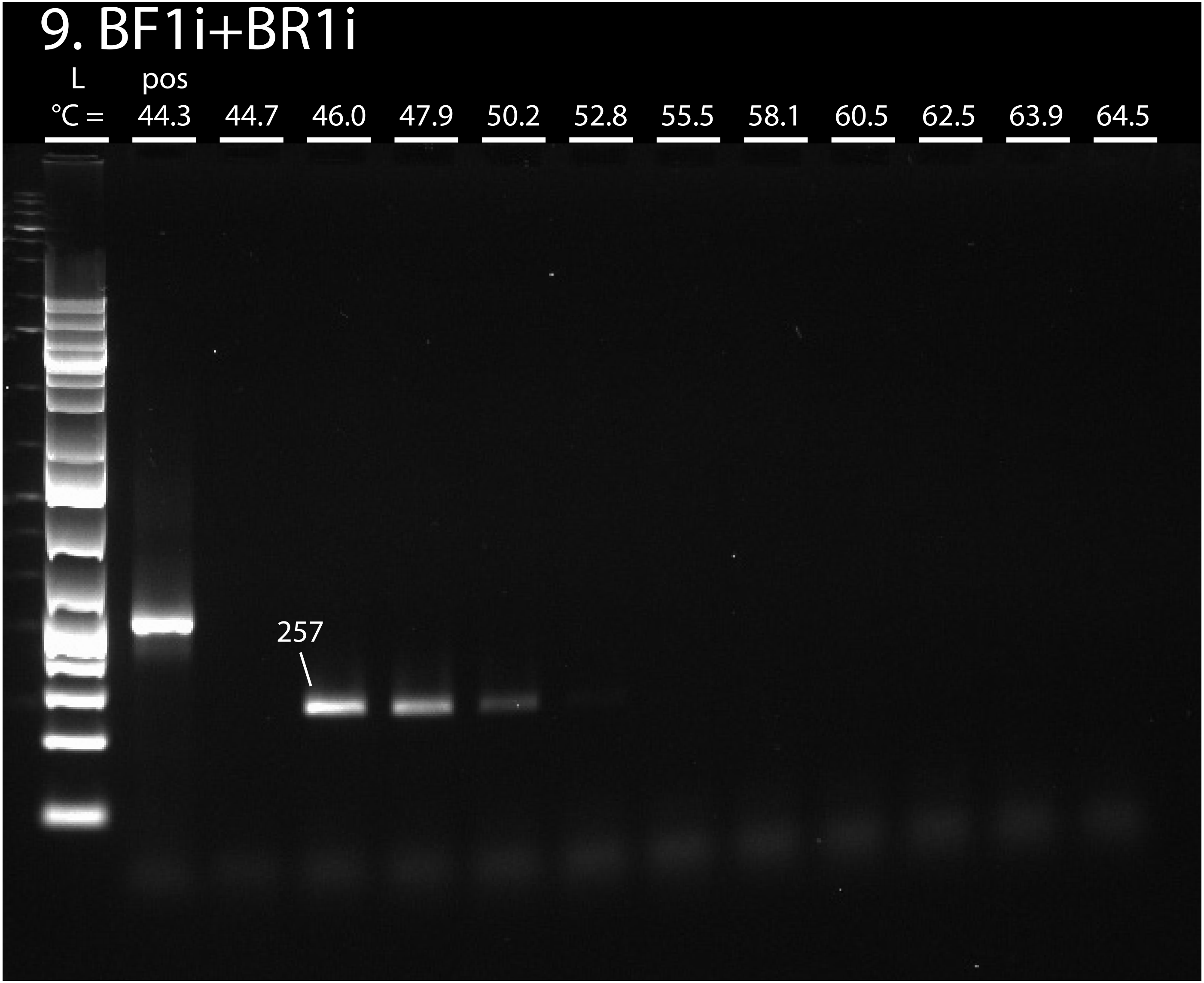
9. BF1i+BR1i
L
pos
°C =
44.3
44.7
46.0
47.9
50.2
52.8
55.5
58.1
60.5
62.5
63.9
64.5
257

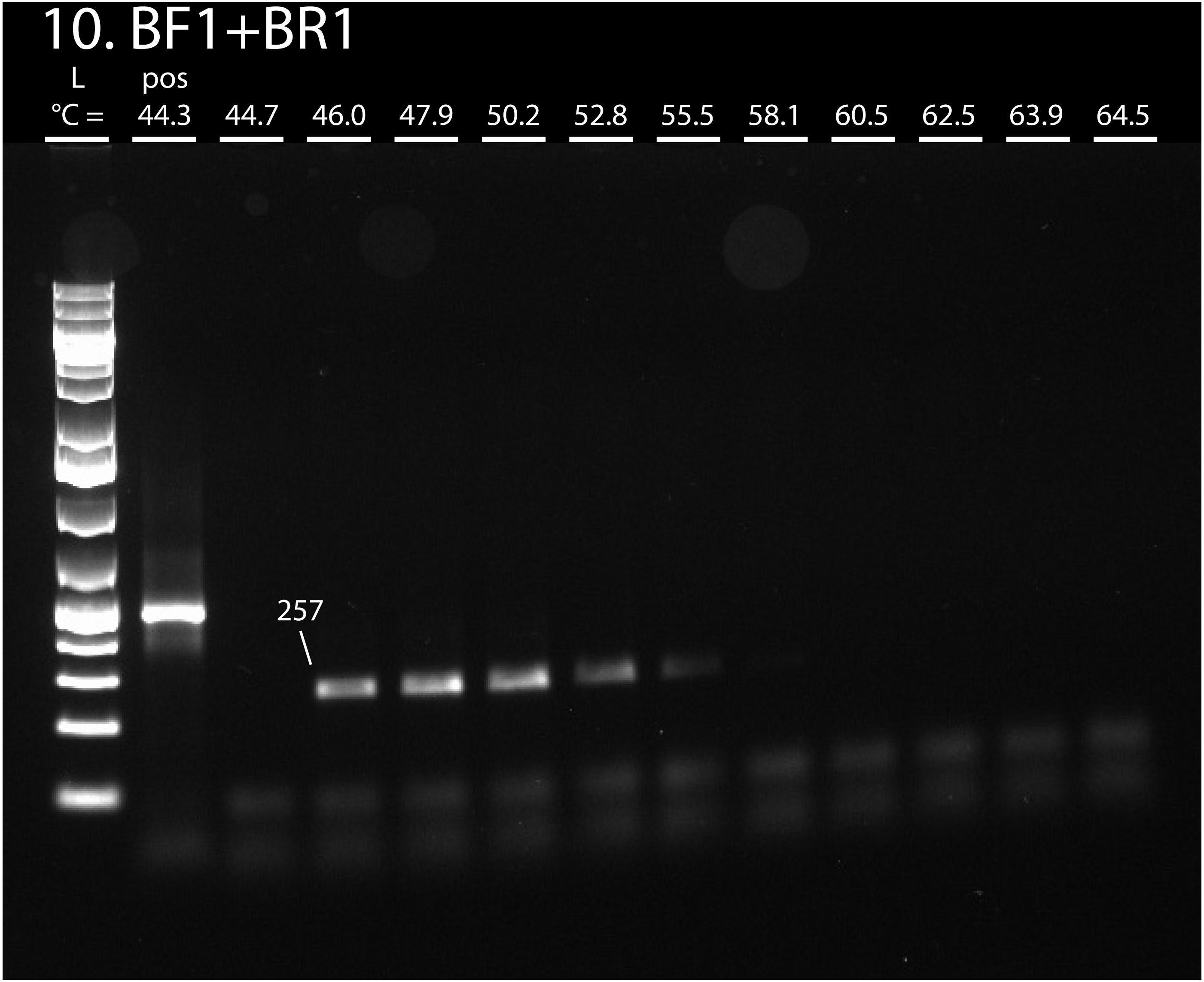
10. BF1+BR1
L
pos
°C =
44.3
44.7
46.0
47.9
50.2
52.8
55.5
58.1
60.5
62.5
63.9
64.5
257

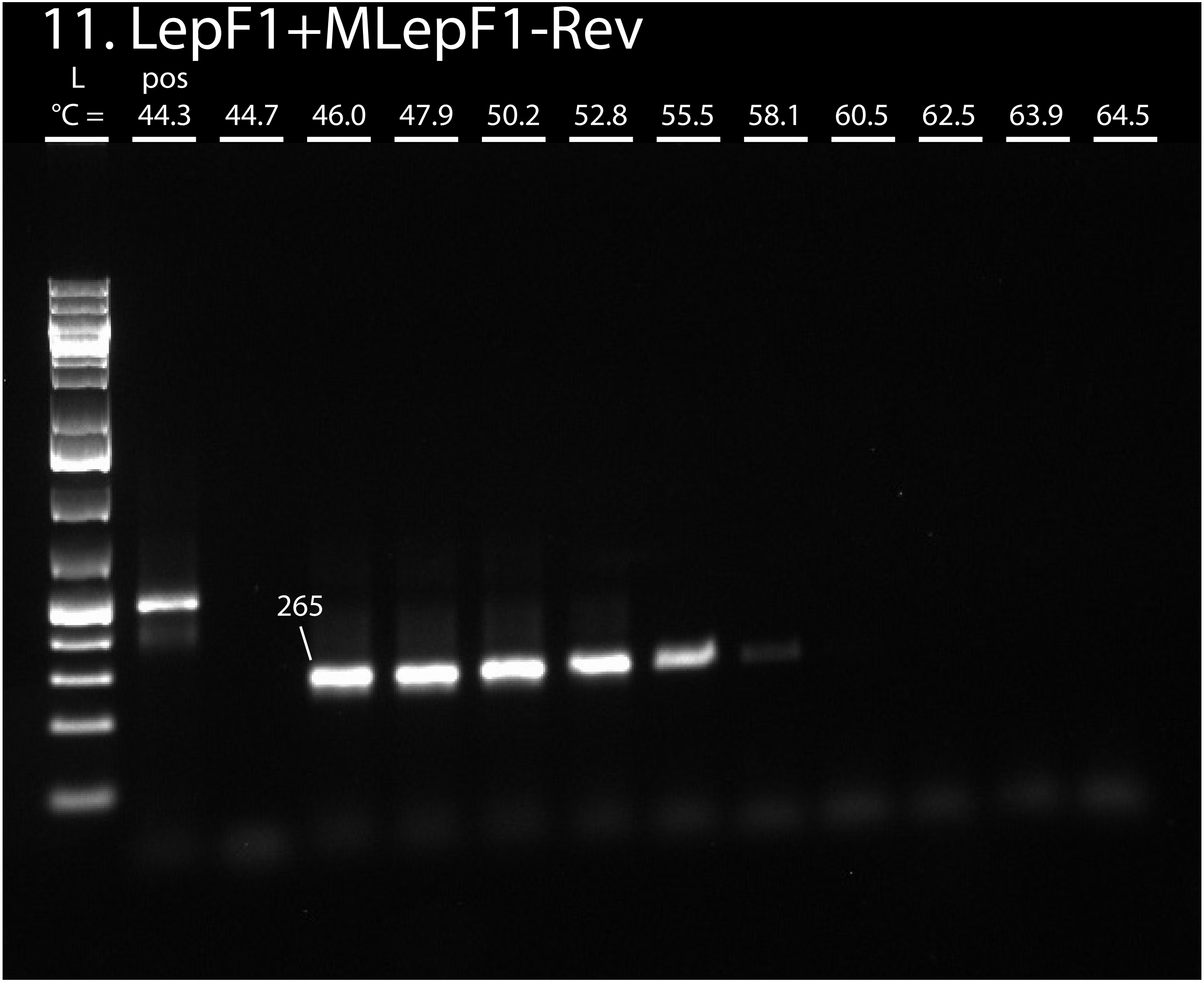
11. LepF1+MLepF1-Rev
L
pos
°C =
44.3
44.7
46.0
47.9
50.2
52.8
55.5
58.1
60.5
62.5
63.9
64.5
265

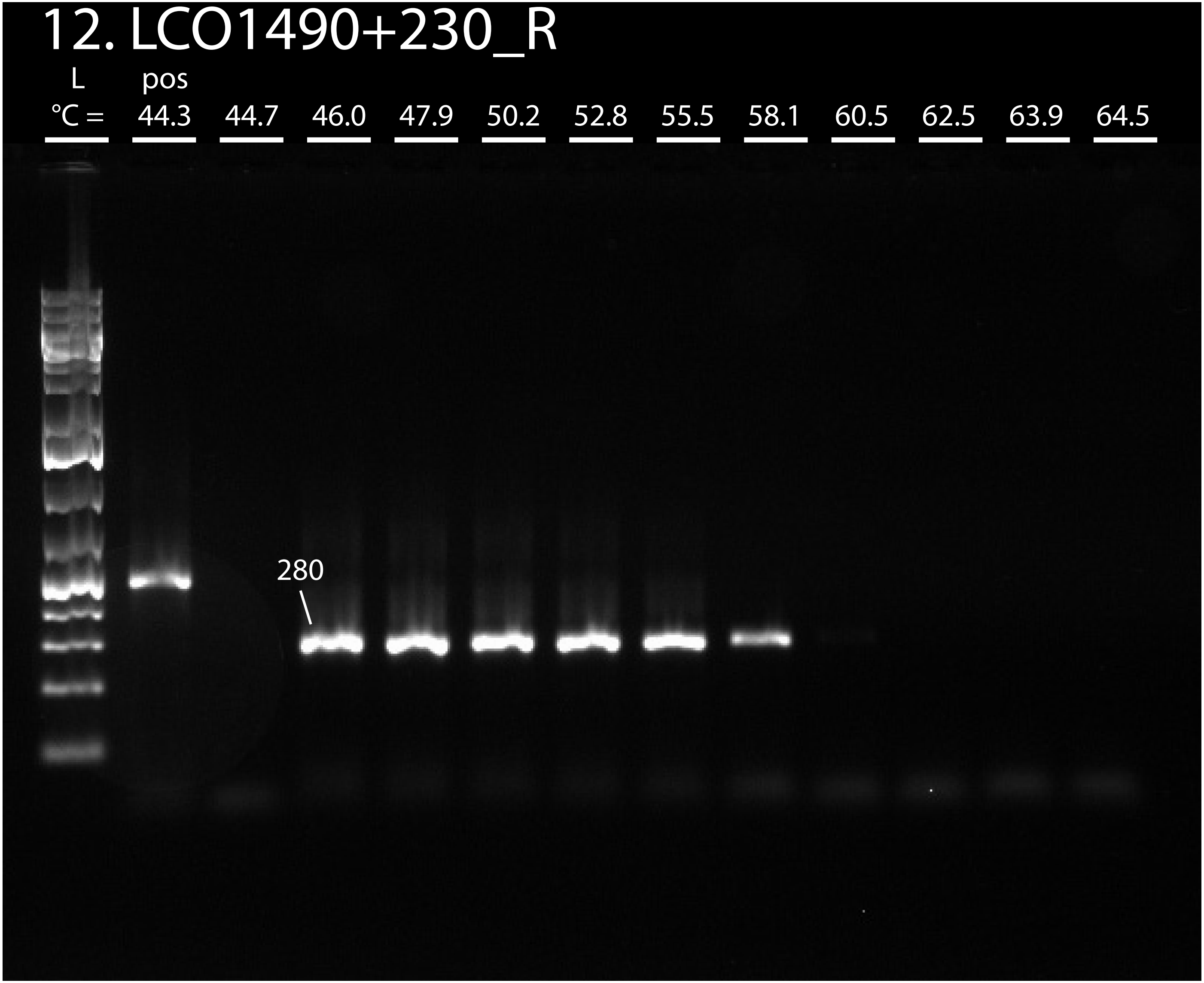
12. LCO1490+230_R
L
pos
°C =
44.3
44.7
46.0
47.9
50.2
52.8
55.5
58.1
60.5
62.5
63.9
64.5
280

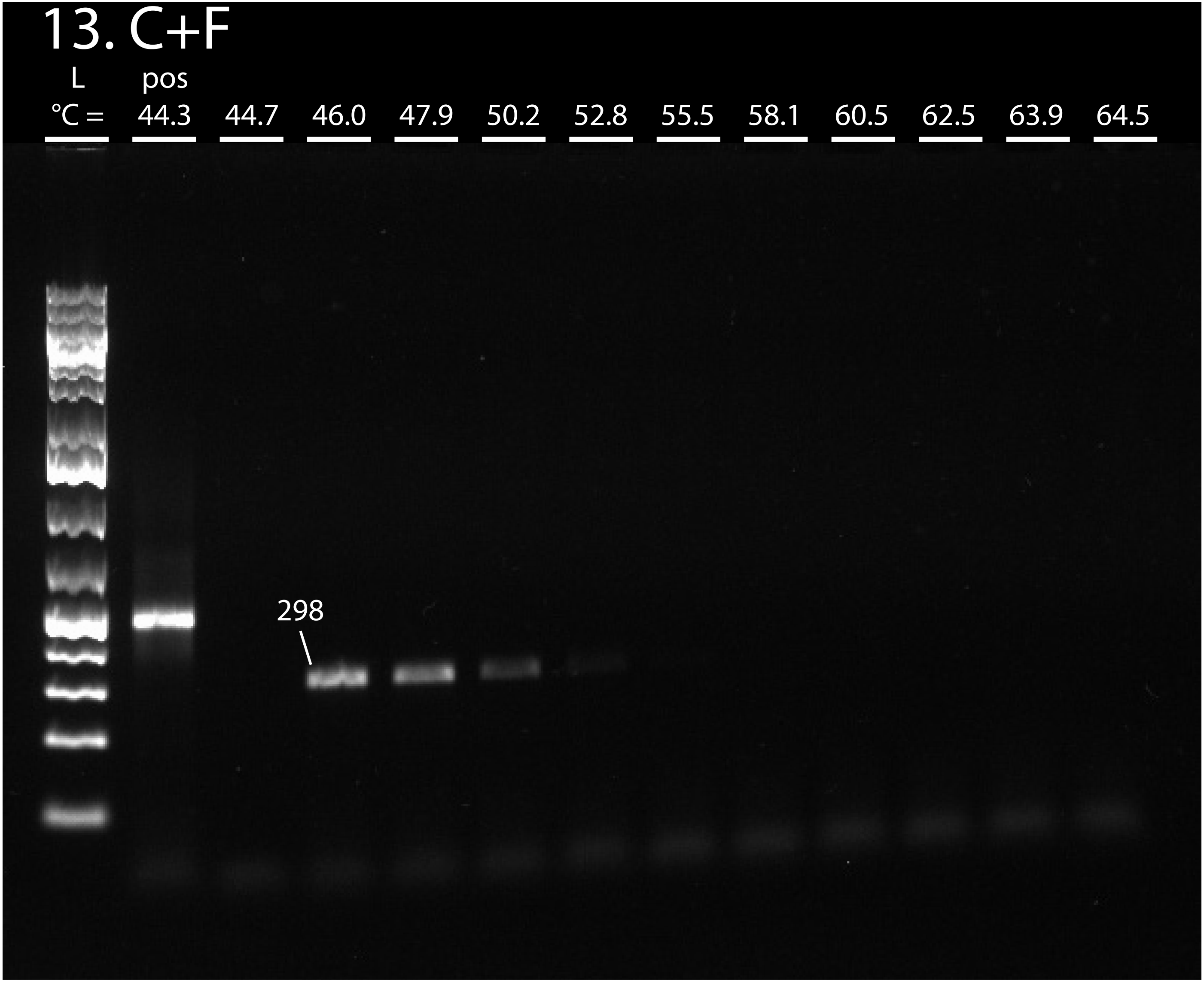
13. C+F
L
pos
°C =
44.3
44.7
46.0
47.9
50.2
52.8
55.5
58.1
60.5
62.5
63.9
64.5
298

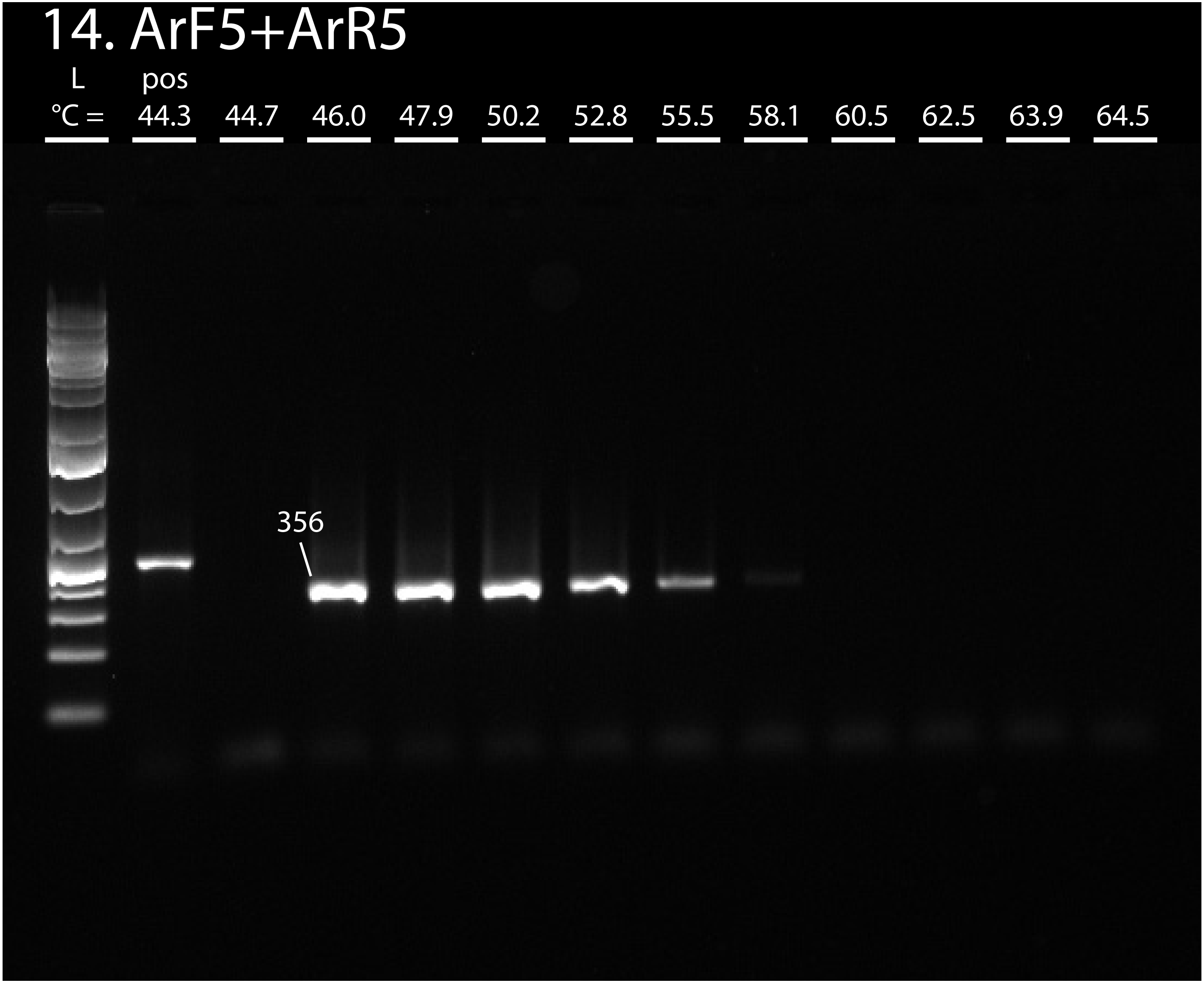
14. ArF5+ArR5
L
pos
°C =
44.3
44.7
46.0
47.9
50.2
52.8
55.5
58.1
60.5
62.5
63.9
64.5
356

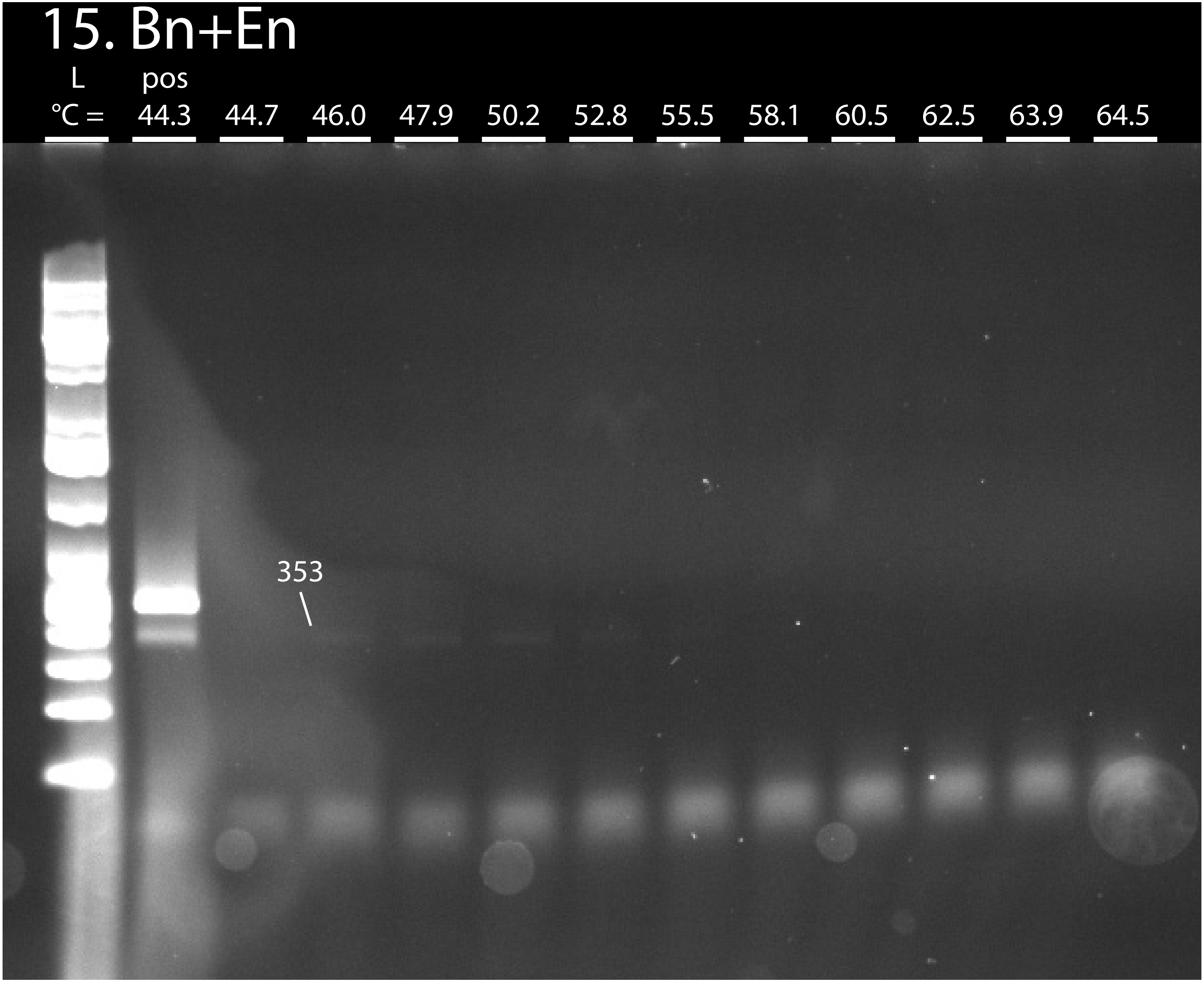
15. Bn+En
L
pos
°C =
44.3
44.7
46.0
47.9
50.2
52.8
55.5
58.1
60.5
62.5
63.9
64.5
353

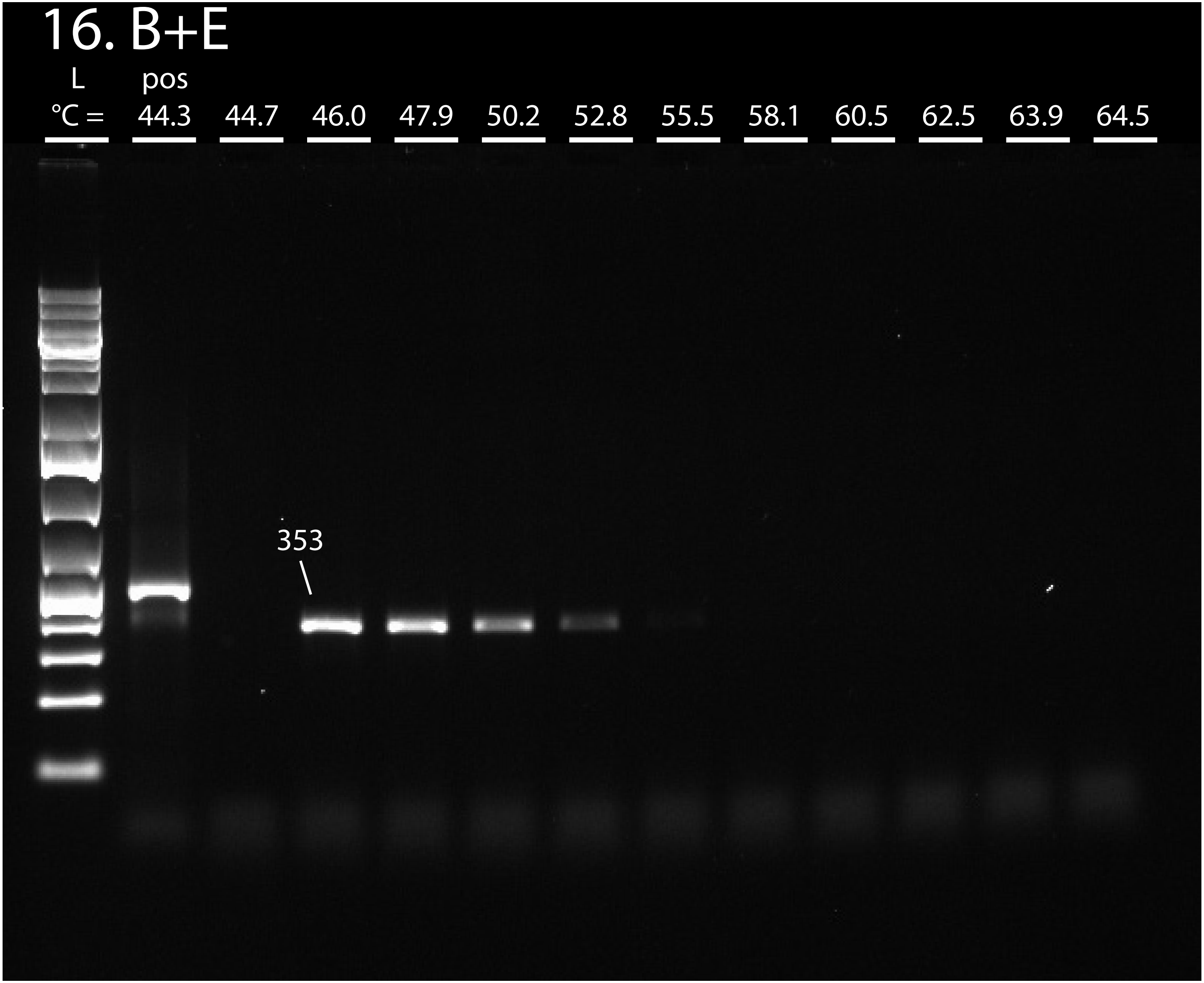
16. B+E
L
pos
°C =
44.3
44.7
46.0
47.9
50.2
52.8
55.5
58.1
60.5
62.5
63.9
64.5
353

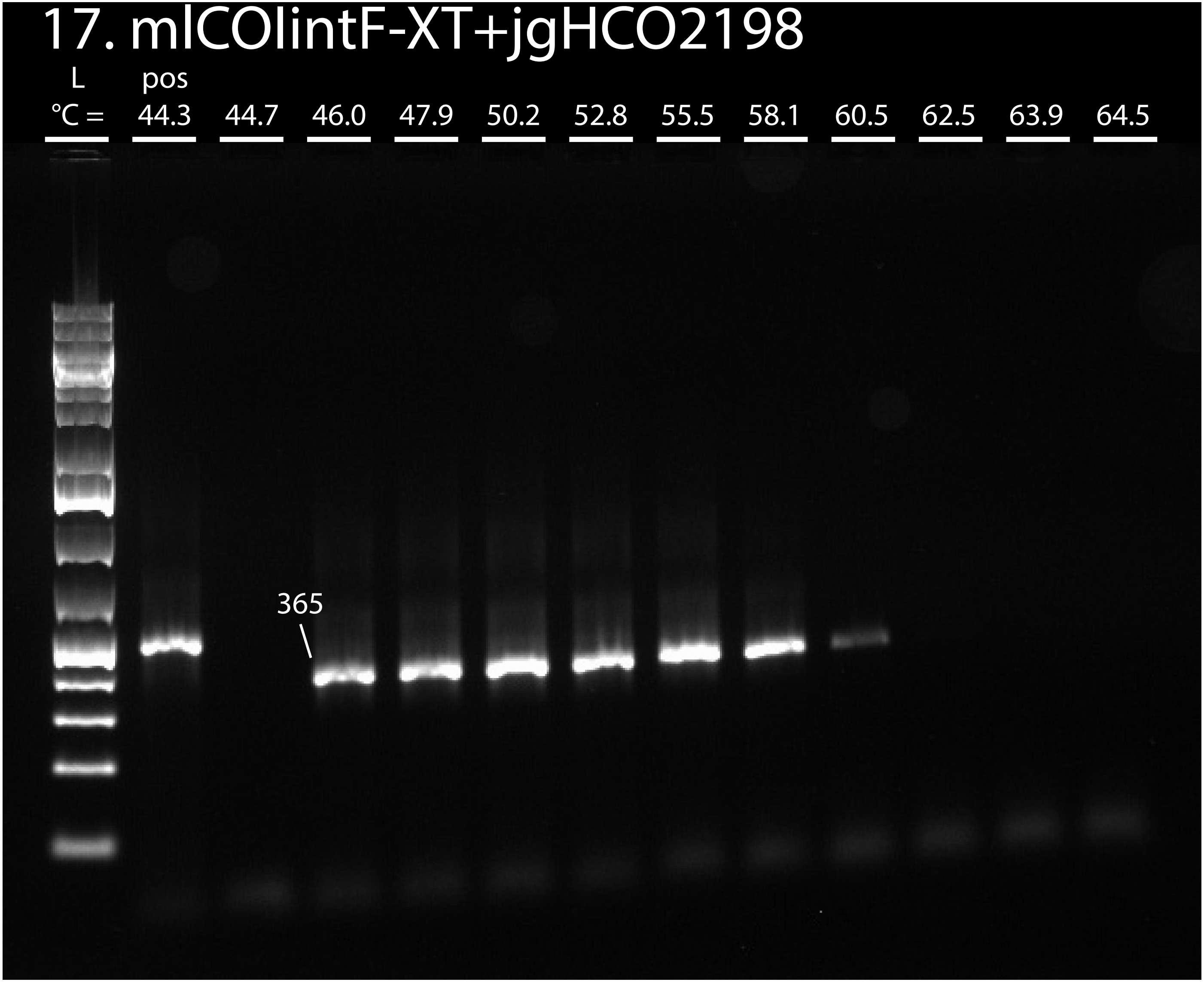
17. mlCOIintF-XT+jgHCO2198
L
pos
°C =
44.3
44.7
46.0
47.9
50.2
52.8
55.5
58.1
60.5
62.5
63.9
64.5
365

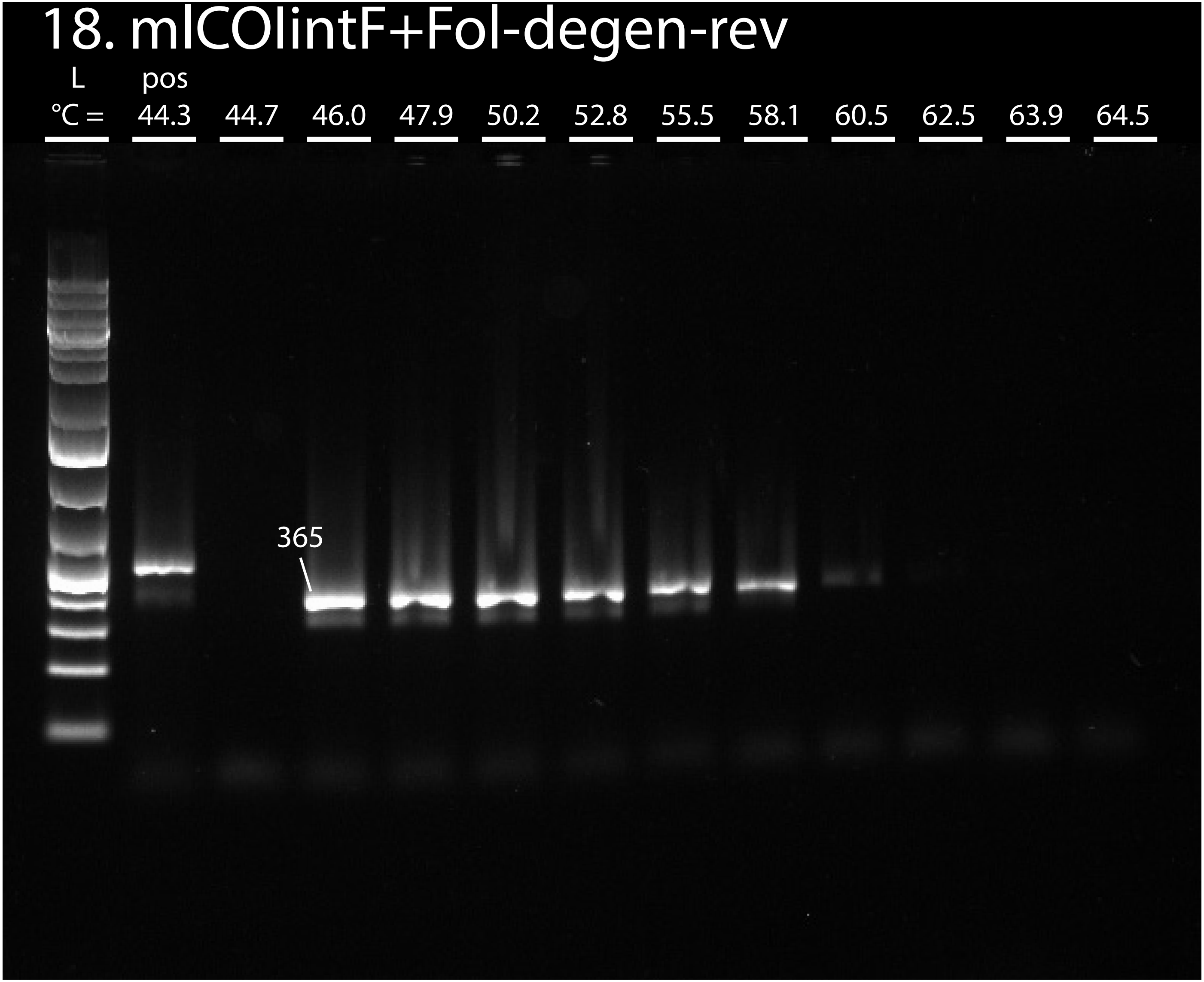
18. mlCOIintF+Fol-degen-rev
L
pos
°C =
44.3
44.7
46.0
47.9
50.2
52.8
55.5
58.1
60.5
62.5
63.9
64.5
365

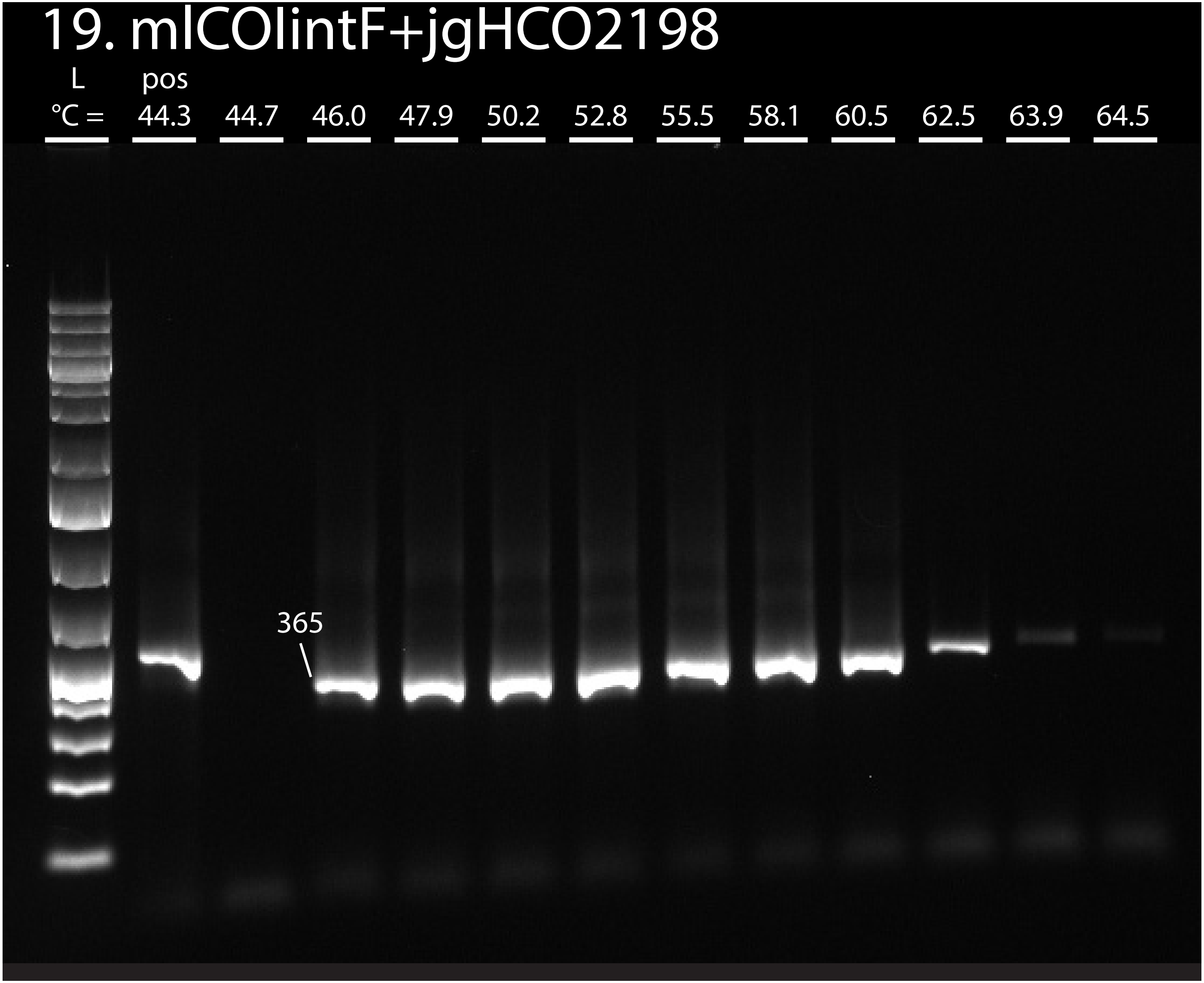
19. mlCOIintF+jgHCO2198
L
pos
°C =
44.3
44.7
46.0
47.9
50.2
52.8
55.5
58.1
60.5
62.5
63.9
64.5
365

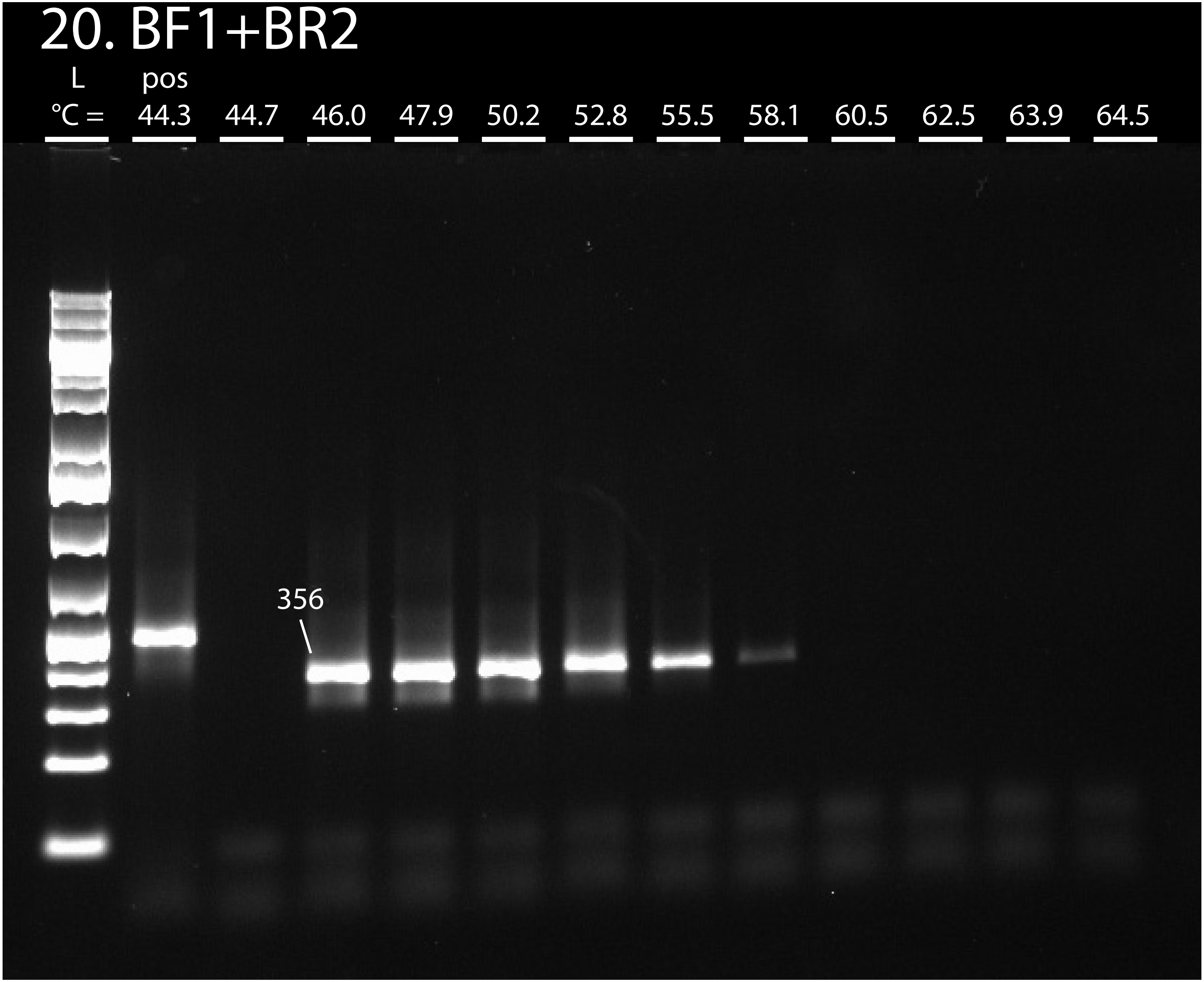
20. BF1+BR2
L
pos
°C =
44.3
44.7
46.0
47.9
50.2
52.8
55.5
58.1
60.5
62.5
63.9
64.5
356

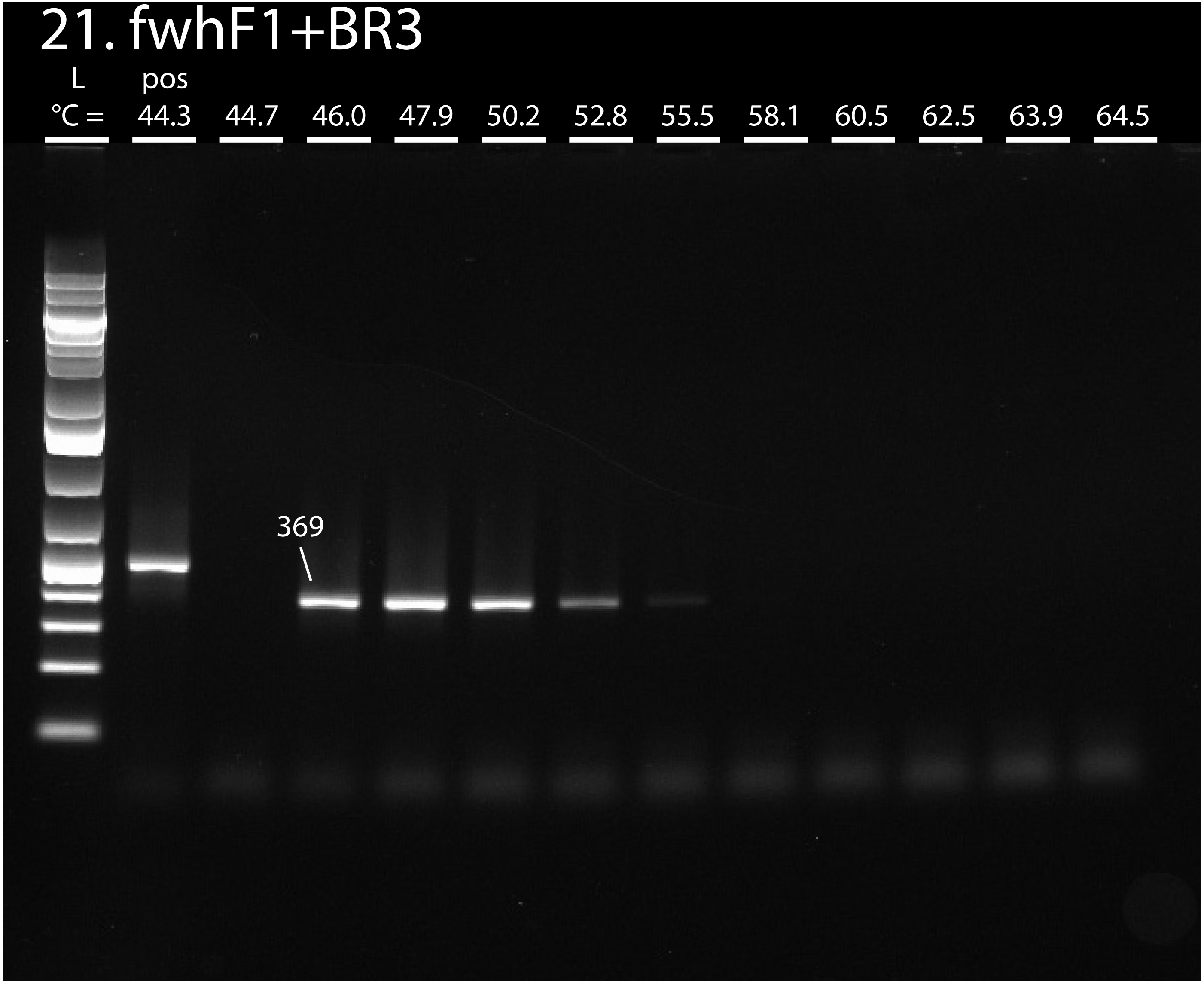
21. fwhF1+BR3
L
pos
°C =
44.3
44.7
46.0
47.9
50.2
52.8
55.5
58.1
60.5
62.5
63.9
64.5
369

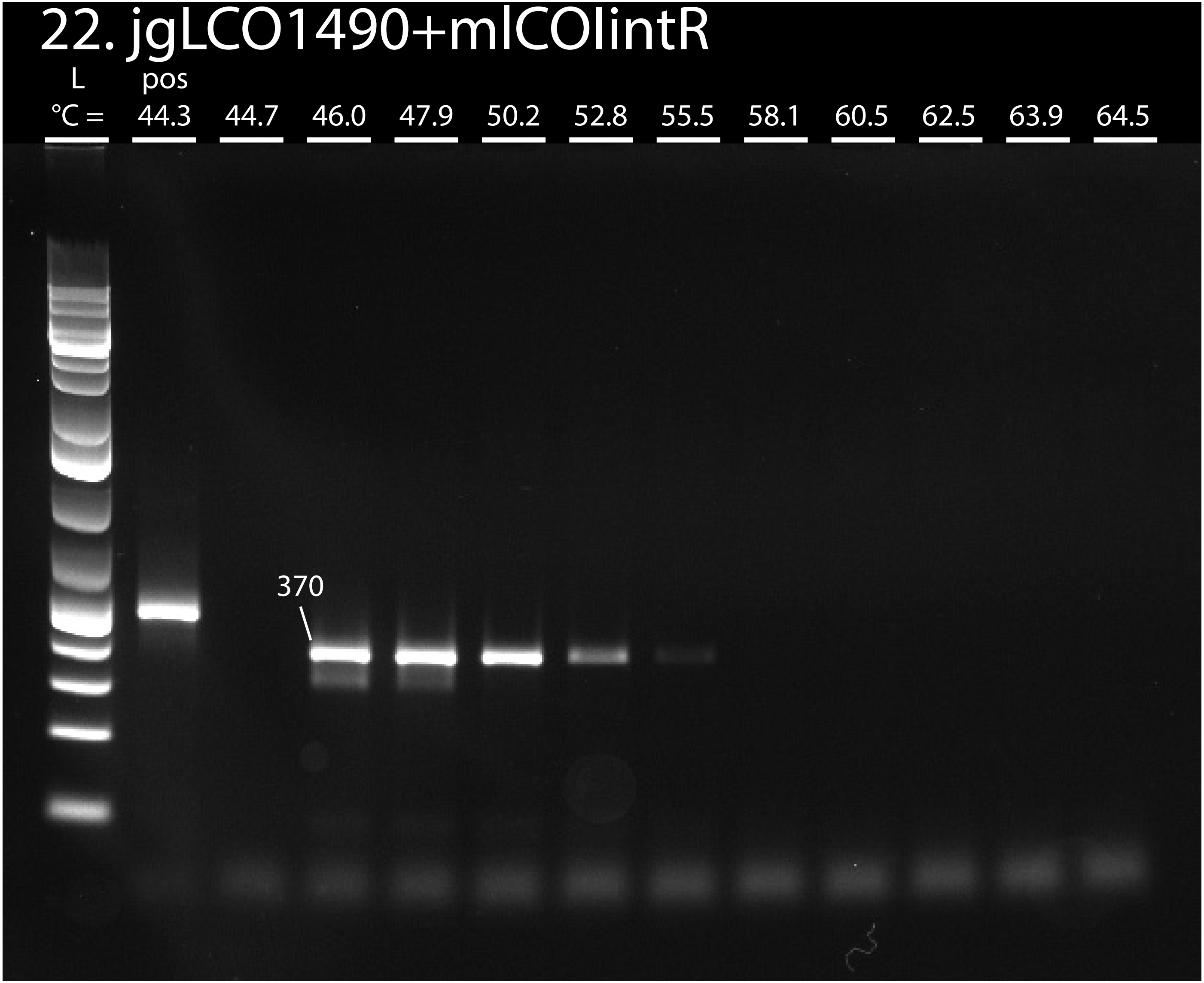
22. jgLCO1490+mlCOintR
L
pos
°C =
44.3
44.7
46.0
47.9
50.2
52.8
55.5
58.1
60.5
62.5
63.9
64.5
370

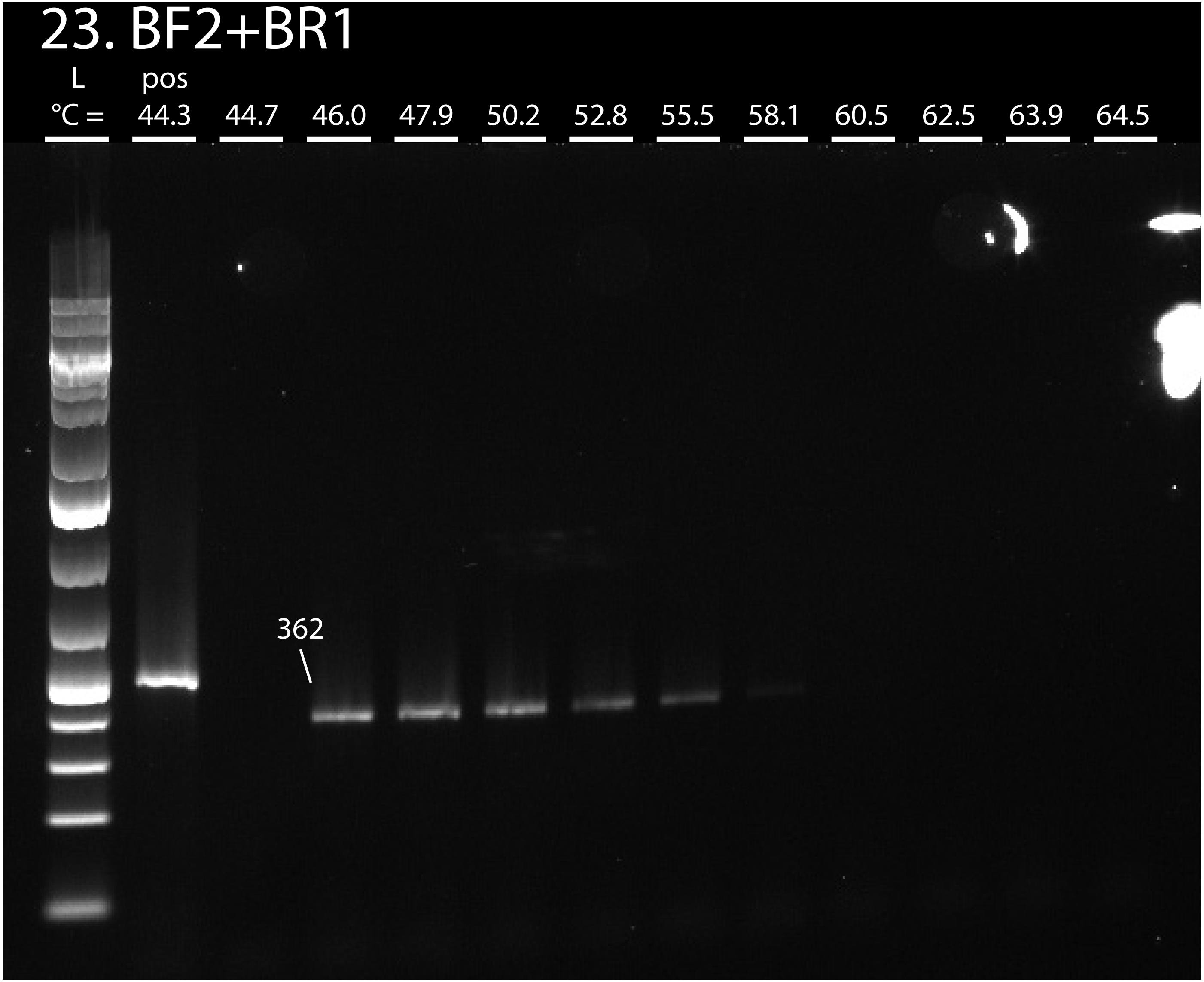
23. BF2+BR1
L
pos
°C =
44.3
44.7
46.0
47.9
50.2
52.8
55.5
58.1
60.5
62.5
63.9
64.5
362

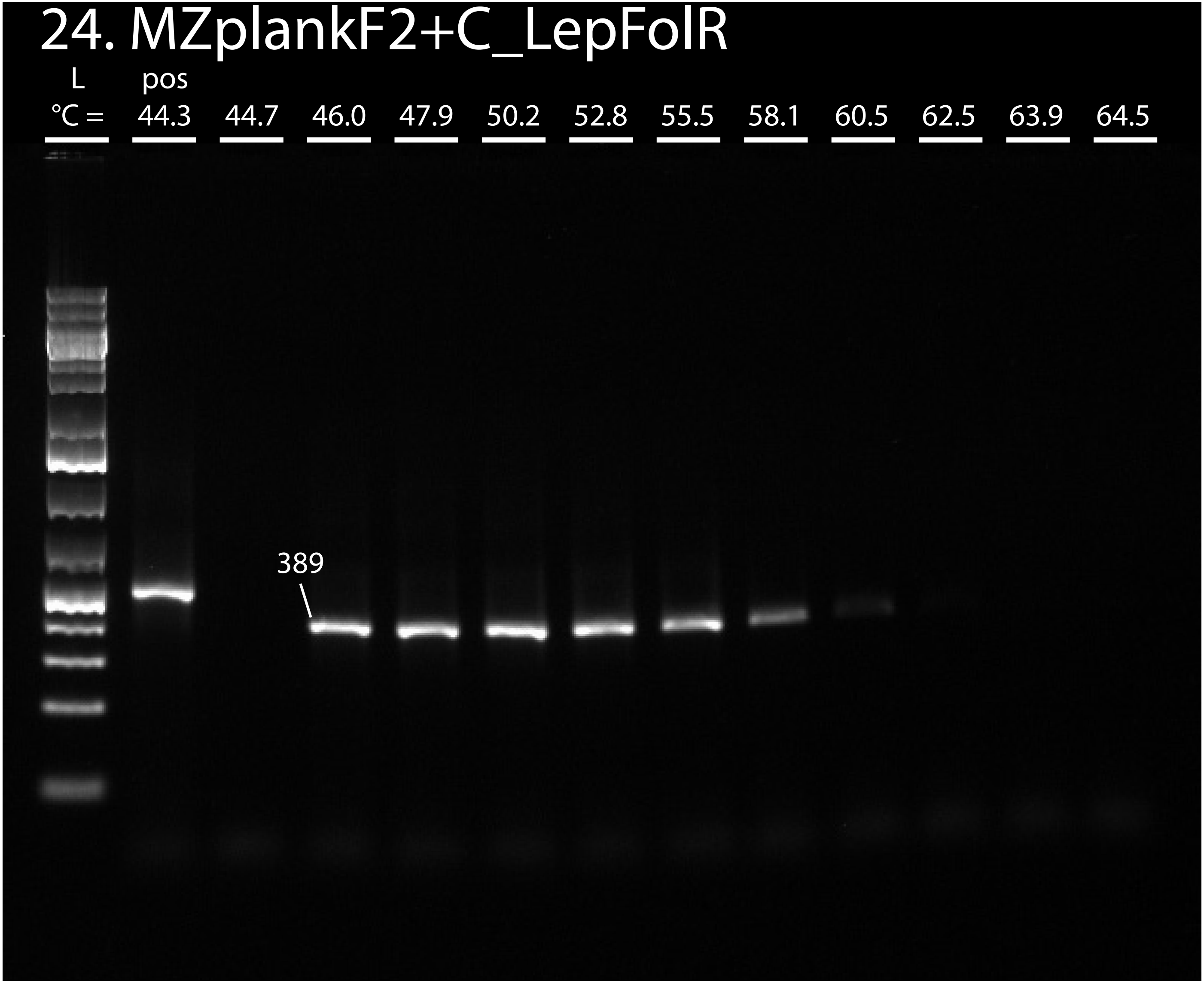
24. MZplankF2+C_LepFolR
L
pos
°C =
44.3
44.7
46.0
47.9
50.2
52.8
55.5
58.1
60.5
62.5
63.9
64.5
389

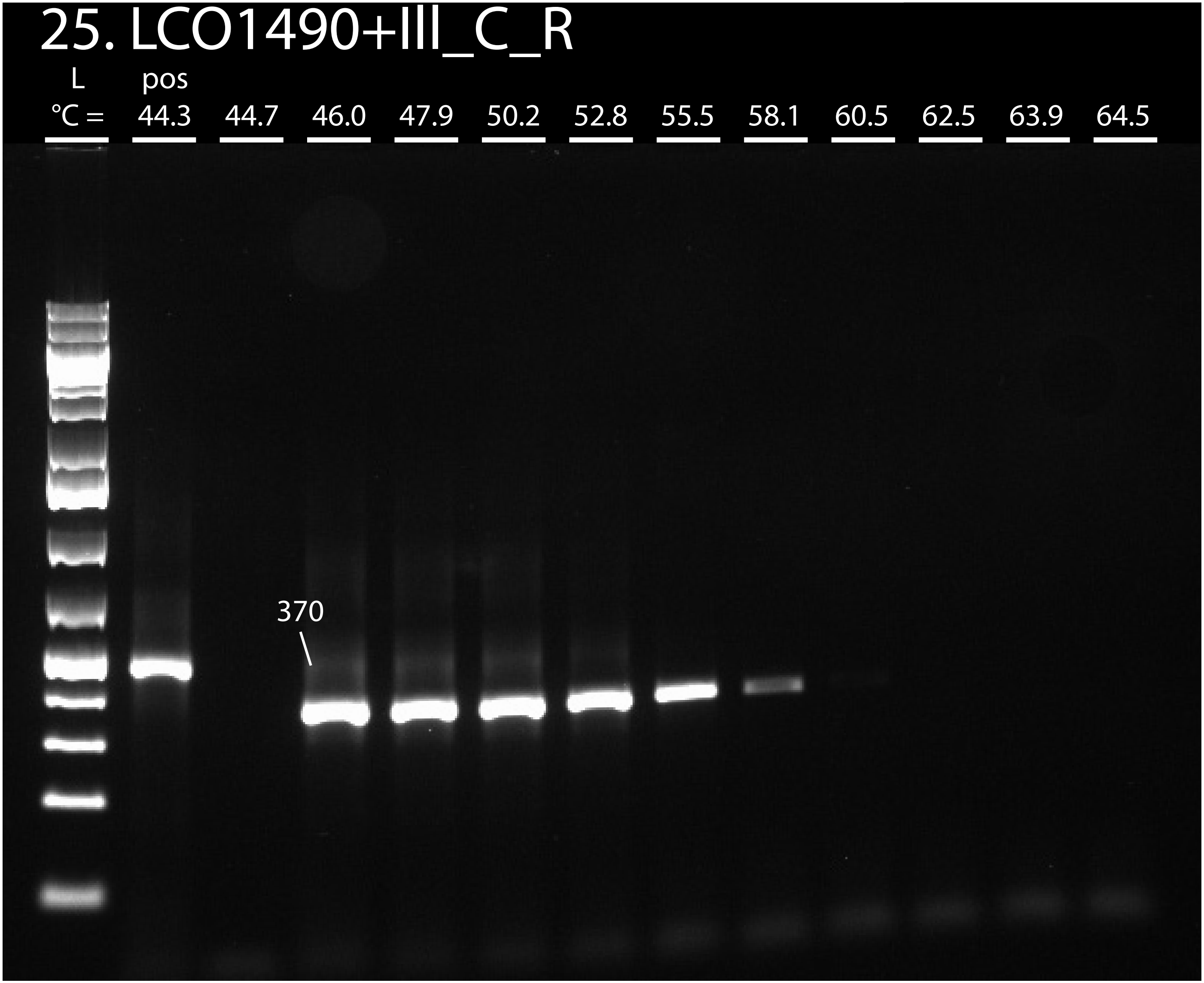

25. LCO1490+lll_C_R
L
pos
°C =
44.3
44.7
46.0
47.9
50.2
52.8
55.5
58.1
60.5
62.5
63.9
64.5
370

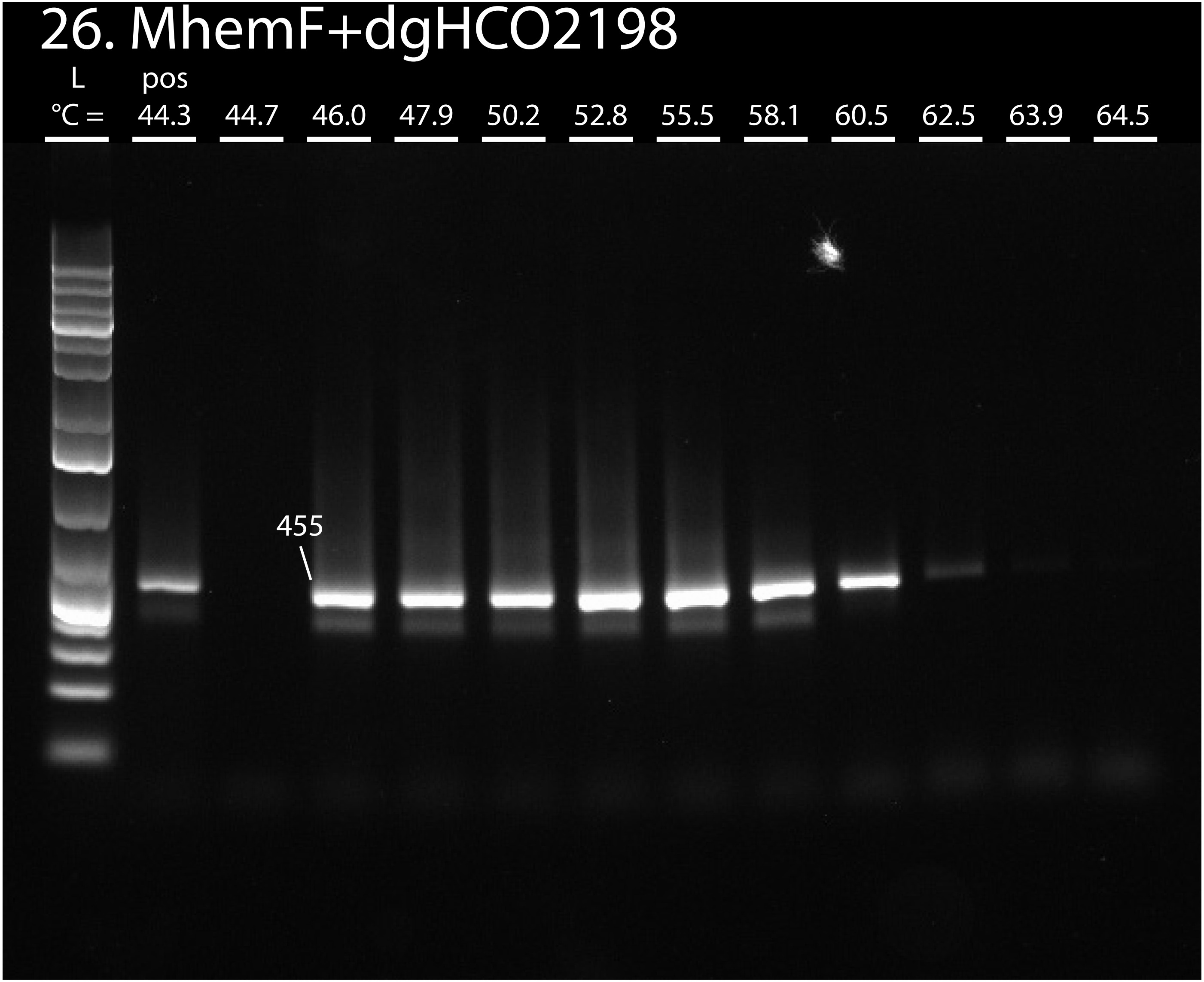
26. MhemF+dgHCO2198
L
pos
°C =
44.3
44.7
46.0
47.9
50.2
52.8
55.5
58.1
60.5
62.5
63.9
64.5
455

27. MLepF1+C_LepFolR

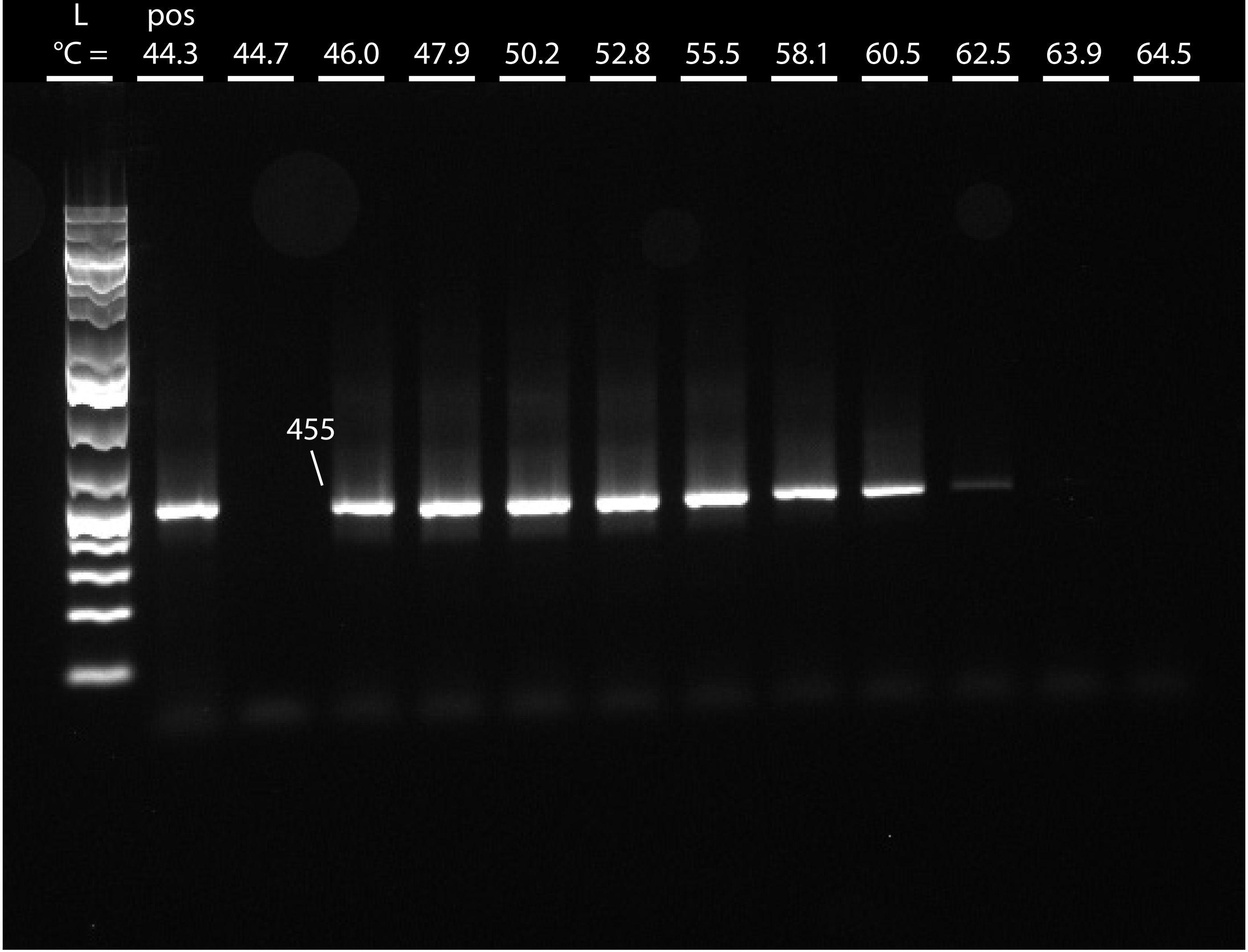

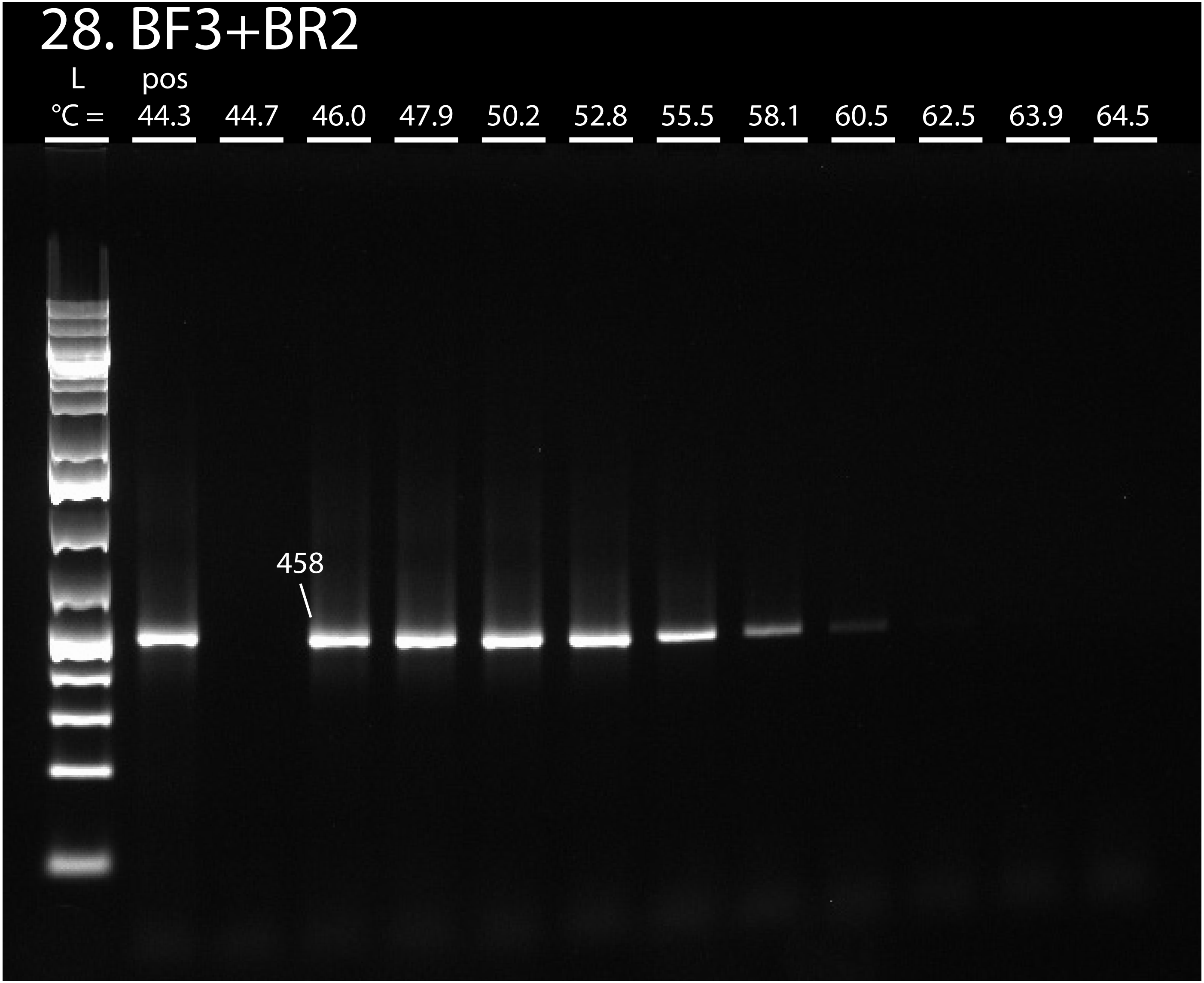

28. BF3+BR2
L
°C =
pos
44.3
44.7
46.0
47.9
50.2
52.8
55.5
58.1
60.5
62.5
63.9
64.5
458

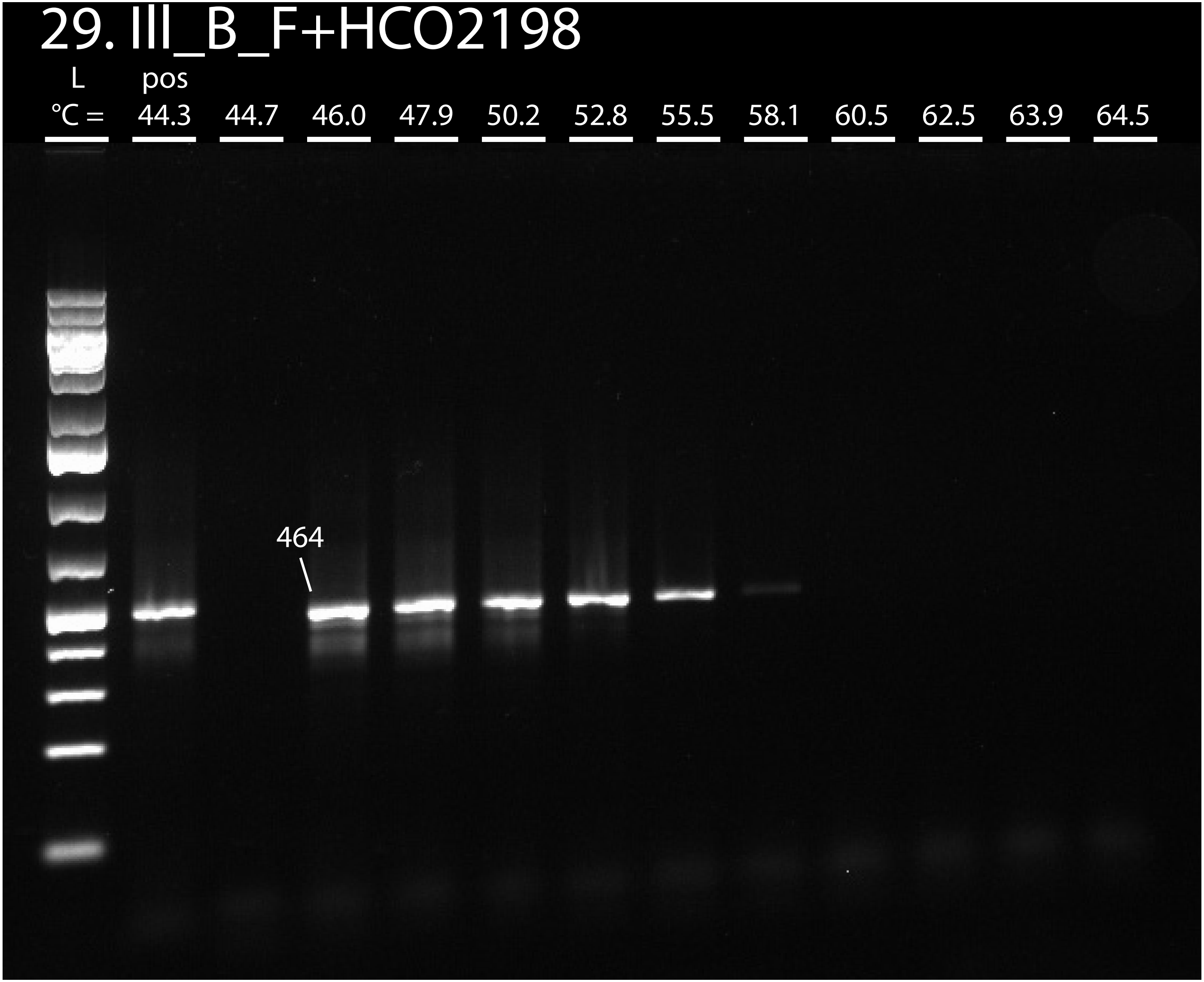
29. III_B_F+HCO2198
L
pos
°C =
44.3
44.7
46.0
47.9
50.2
52.8
55.5
58.1
60.5
62.5
63.9
64.5
464

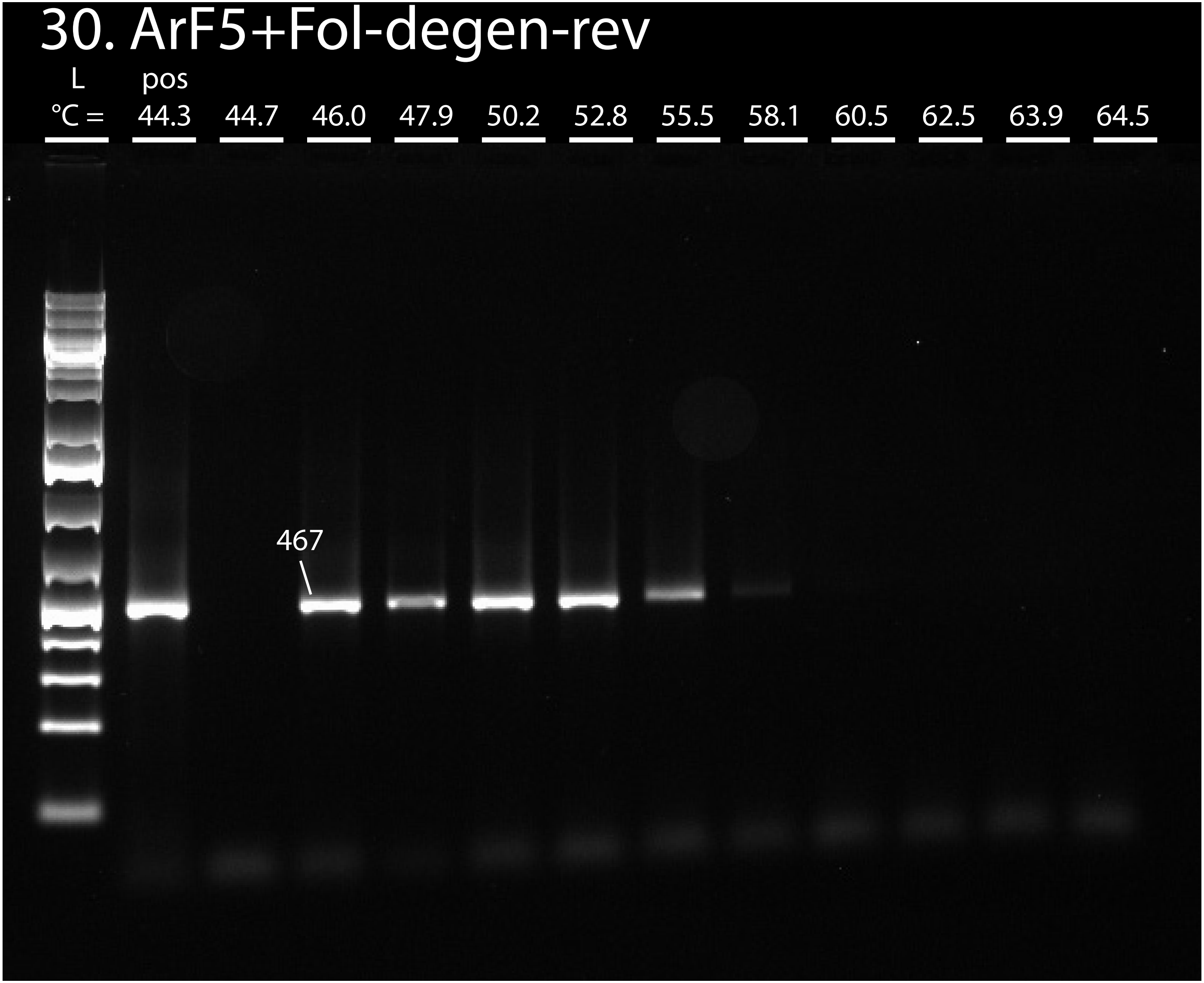
30. ArF5+Fol-degen-rev
L
pos
°C =
44.3
44.7
46.0
47.9
50.2
52.8
55.5
58.1
60.5
62.5
63.9
64.5
467

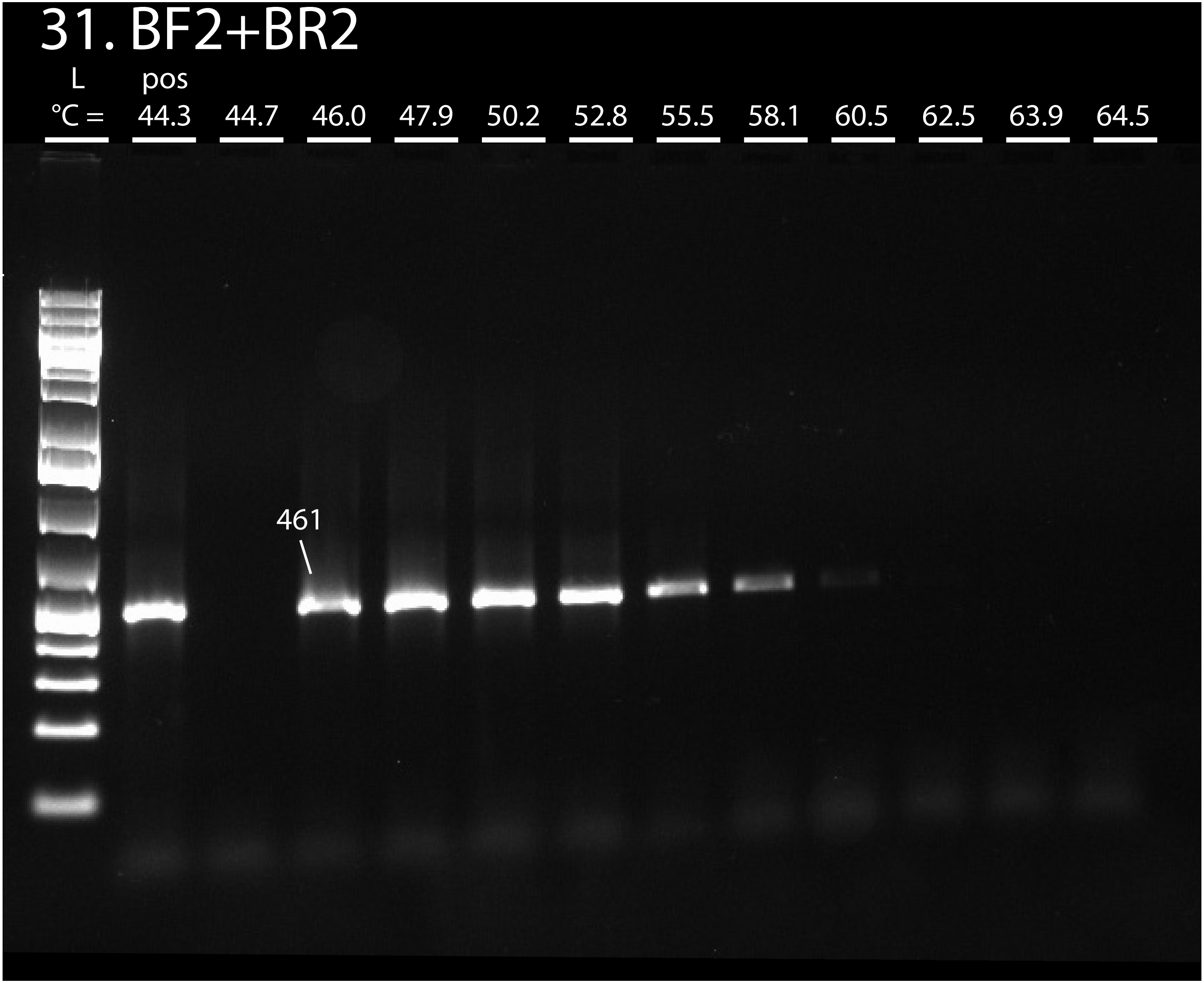
31. BF2+BR2
L
pos
°C =
44.3
44.7
46.0
47.9
50.2
52.8
55.5
58.1
60.5
62.5
63.9
64.5
461

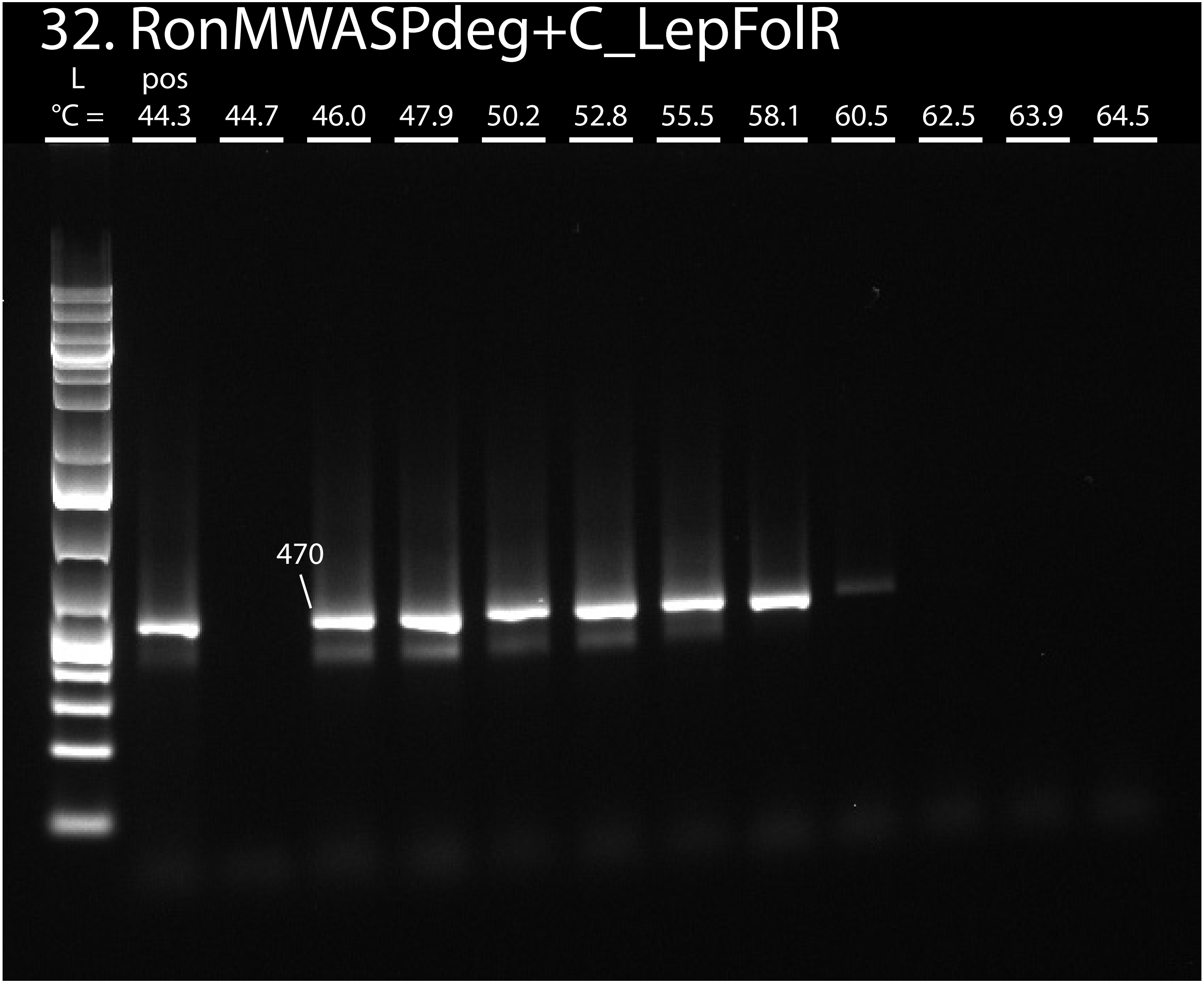

32. RonMWASPdeg+C_LepFolR
L
pos
°C =
44.3
44.7
46.0
47.9
50.2
52.8
55.5
58.1
60.5
62.5
63.9
64.5
470

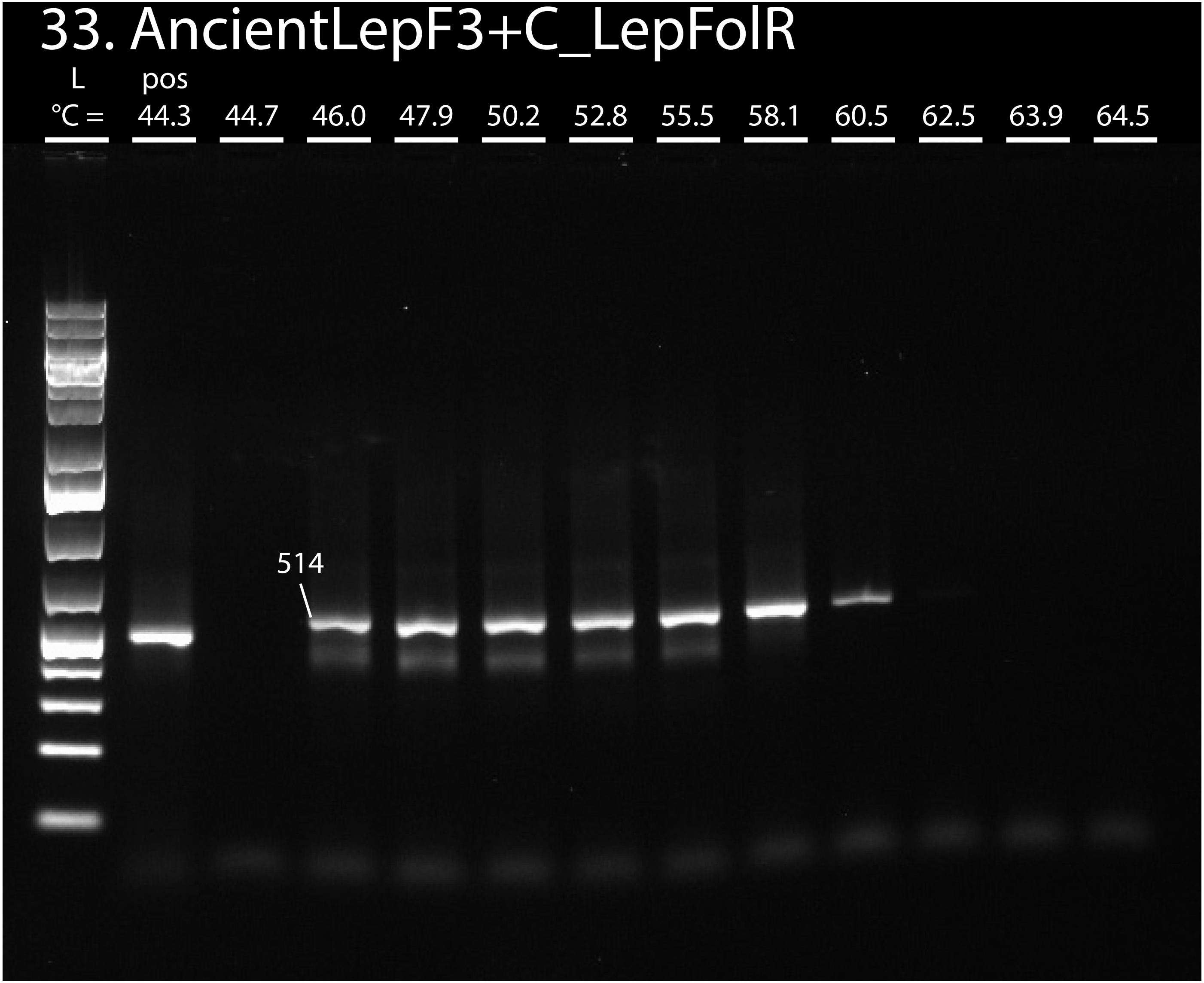
33. AncientLepF3+C_LepFolR
L
pos
°C =
44.3
44.7
46.0
47.9
50.2
52.8
55.5
58.1
60.5
62.5
63.9
64.5
514

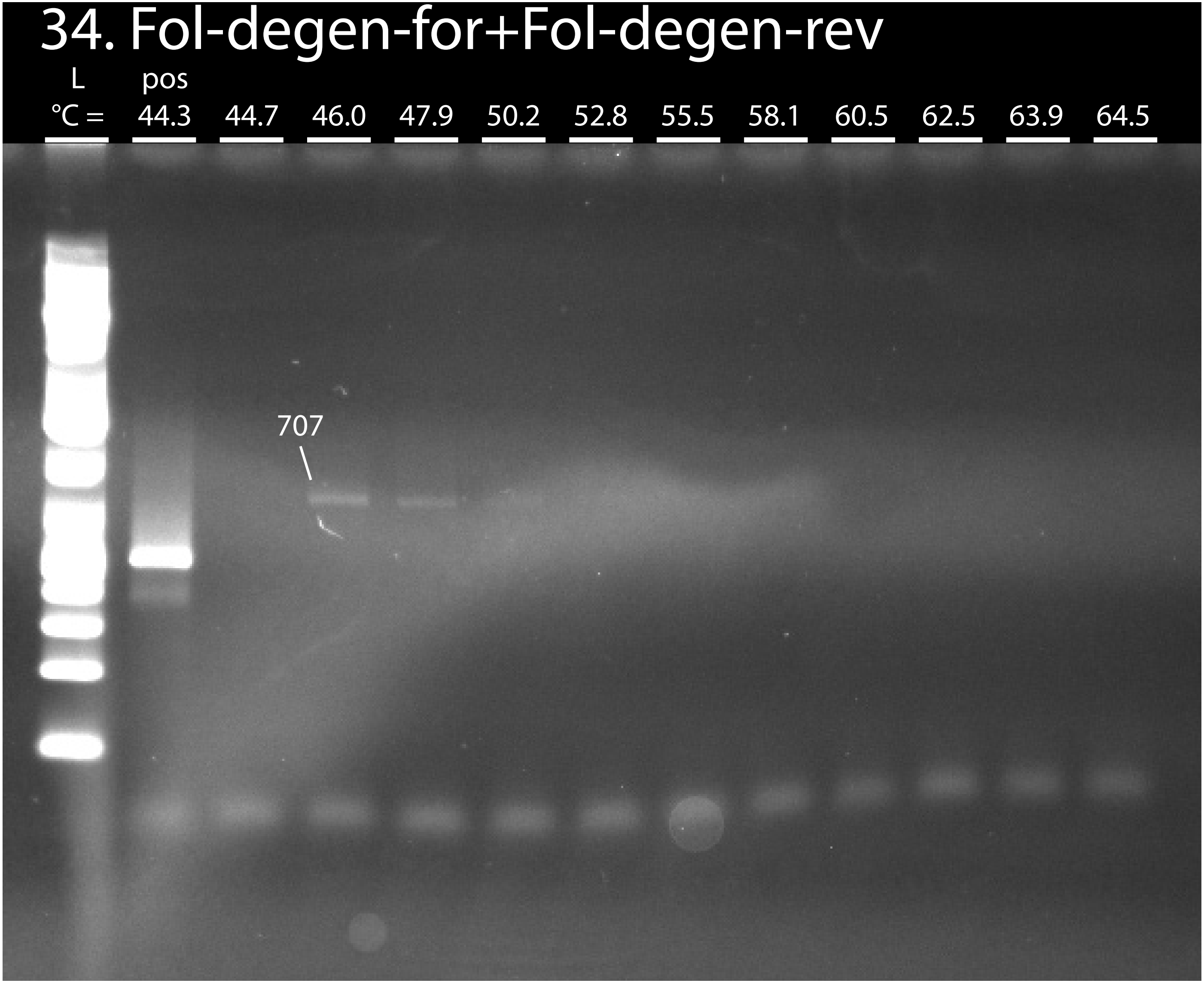
34. Fol-degen-for+Fol-degen-rev
L
pos
°C =
44.3
44.7
46.0
47.9
50.2
52.8
55.5
58.1
60.5
62.5
63.9
64.5
707

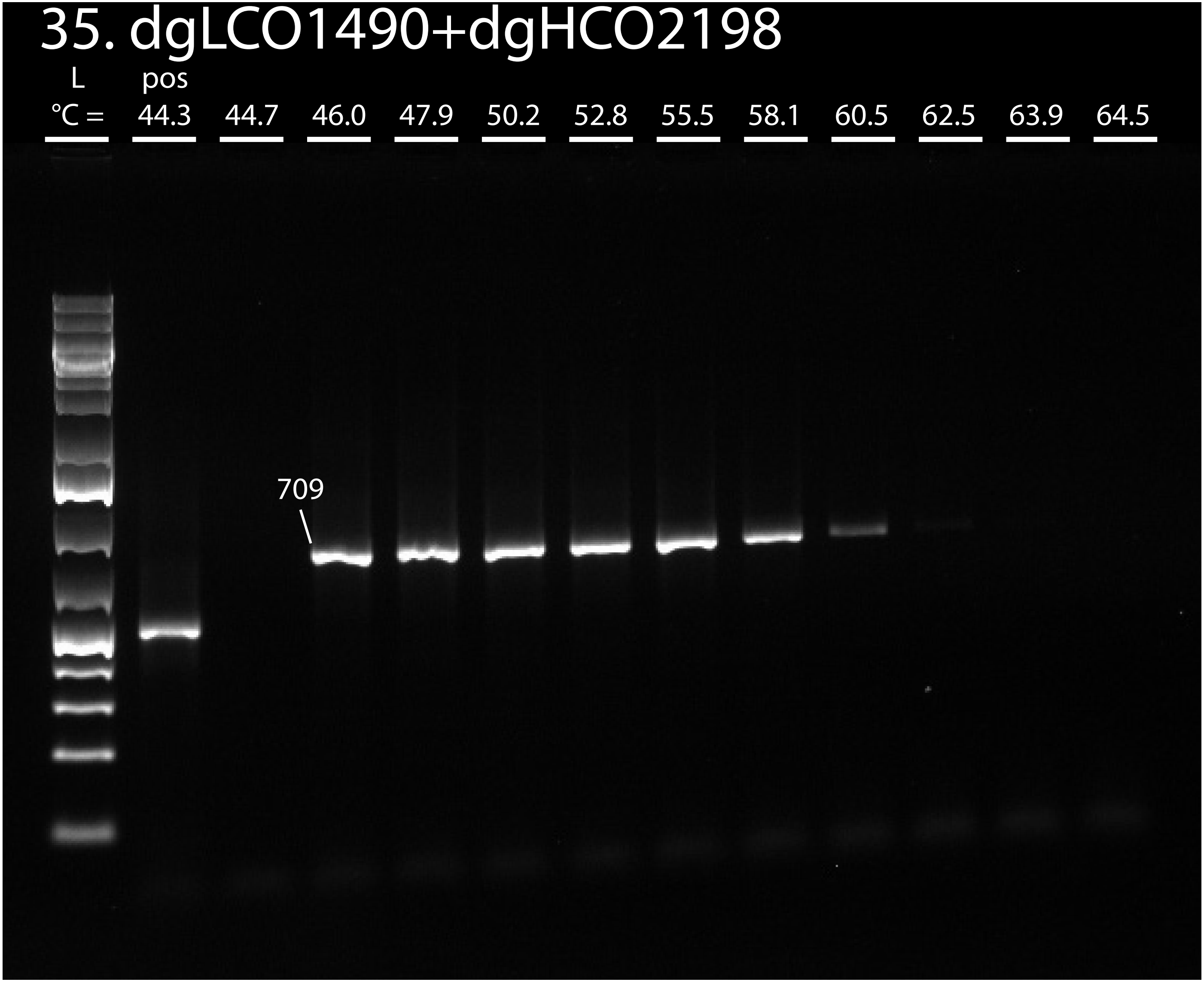
35. dgLCO1490+dgHCO2198
L
pos
°C =
44.3
44.7
46.0
47.9
50.2
52.8
55.5
58.1
60.5
62.5
63.9
64.5
709

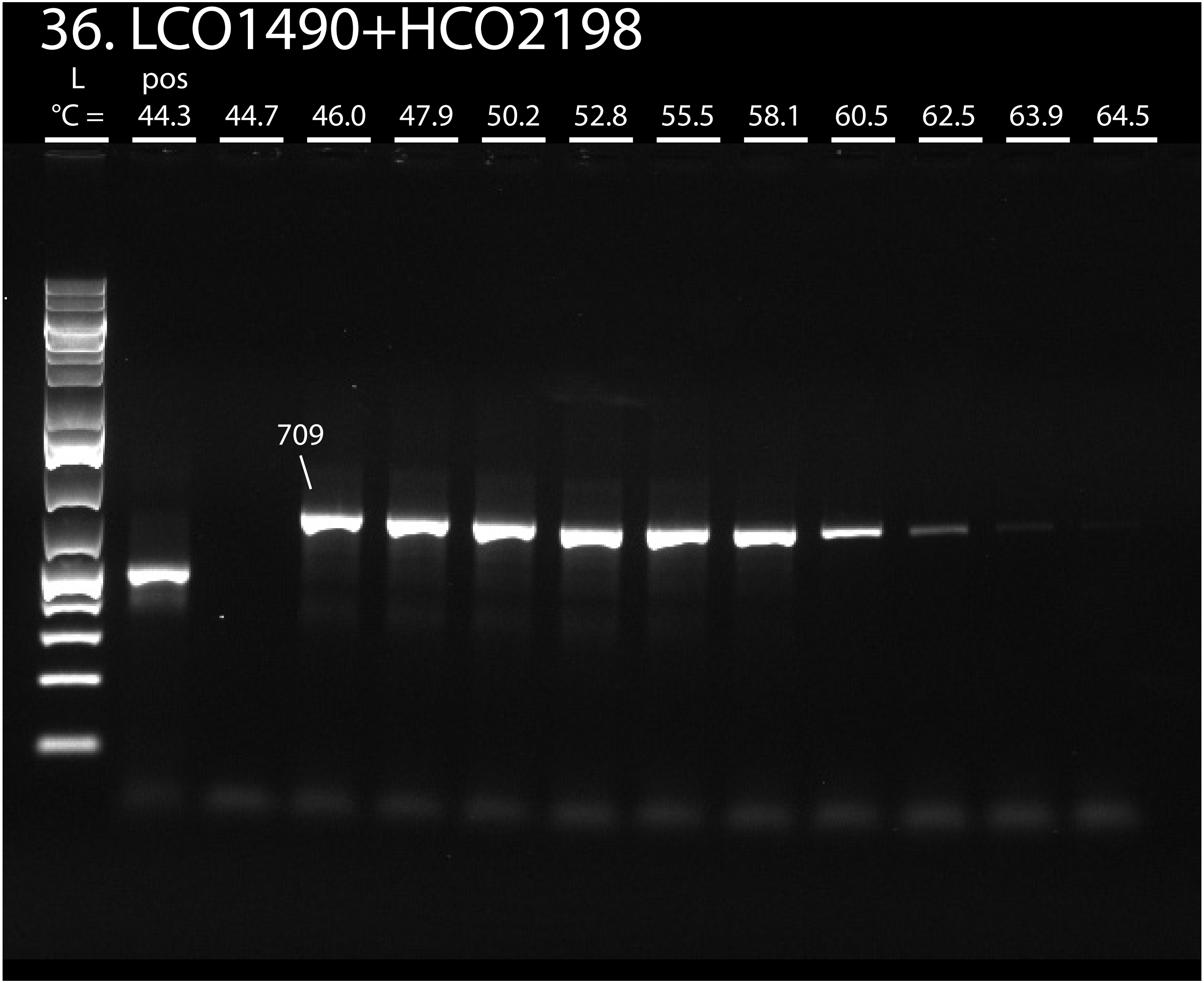
36. LCO1490+HCO2198
L
pos
°C =
44.3
44.7
46.0
47.9
50.2
52.8
55.5
58.1
60.5
62.5
63.9
64.5
709
