# Supplementary material for: Validation of COI metabarcoding primers for terrestrial arthropods
Source: PeerJ. 2019 Oct 7;7:e7745. doi: 10.7717/peerj.7745 (PMC6786254; doi:10.7717/peerj.7745)
Supplement: Figure S8 [file peerj-07-7745-s008.pdf]

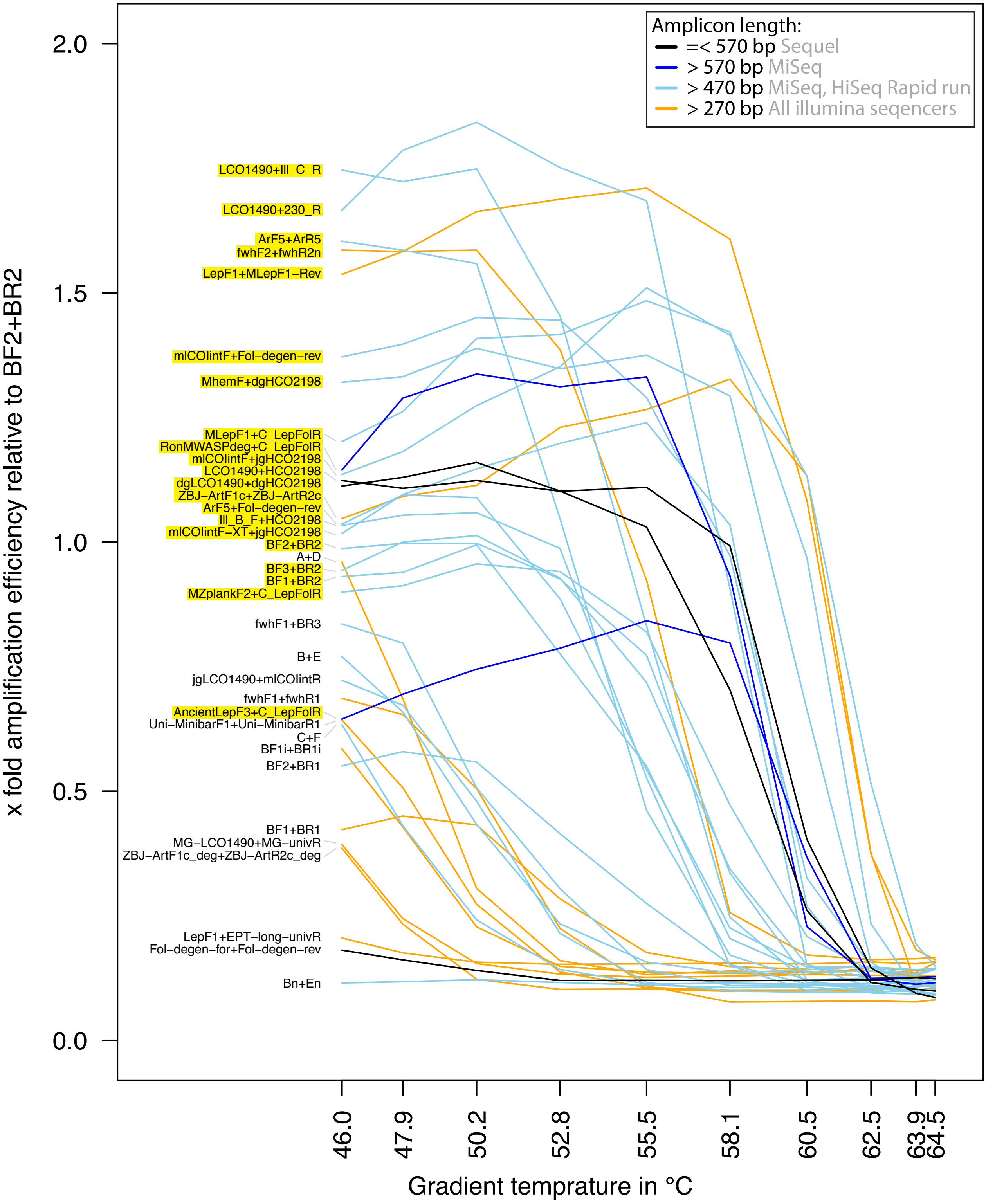

Figure S8: PCR product concentration plotted relative to BF2+BR2 positive control in each sample as different annealing temperatures and 30 PCR cycles. Primer pairs highlighted in Yellow were selected for MiSeq sequencing. Based on the amplicon length the suitability for specific primer combinations for different sequencers in indicated by the line color.
