# Supplementary material for: Validation of COI metabarcoding primers for terrestrial arthropods
Source: PeerJ. 2019 Oct 7;7:e7745. doi: 10.7717/peerj.7745 (PMC6786254; doi:10.7717/peerj.7745)
Supplement: Figure S9 [file peerj-07-7745-s009.pdf]

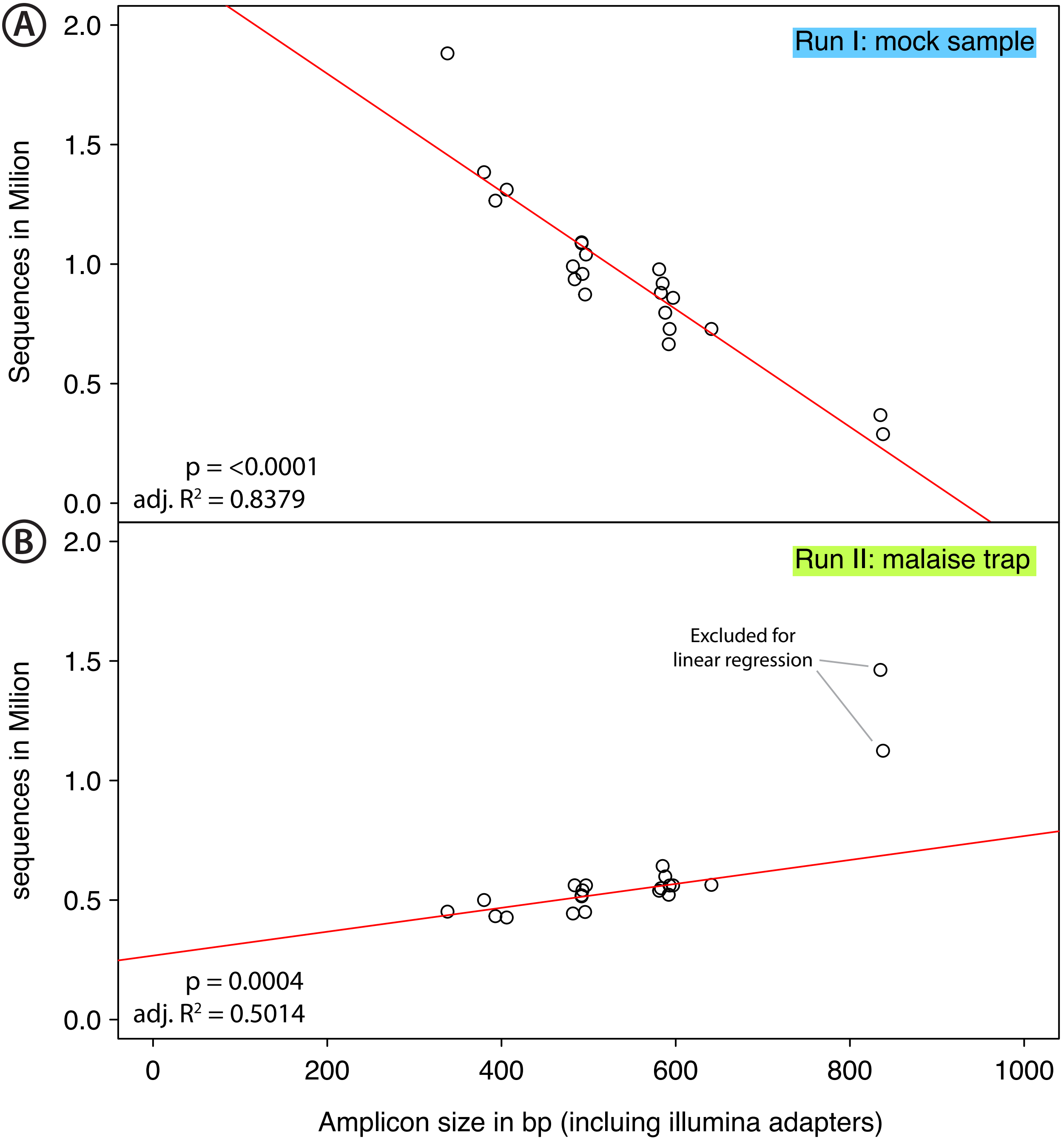

Figure S9: Sequencing depth for the mock sample metabarcoding (A) and malaise trap metabarcoding run (B) plotted against amplicon length including illumina adapters. Red lines indicate linear regression lines. **A**: Data from sequencing the mock sample with 21 primer pairs, amplicons pooled equimollary. Sequequence depth for each marker can be described with the following formula: -0.002462 x Amplicon size in bp + 2.288913, which was used in sucessive runs to account for length bias. **B**: Metabarcoding run on a complete malaise sample, amplicons equimolar pooled for sequencing, but concentration additionally adjusted to account length bias, according to the formular in A. Outlyers LCO1490 + HCO2198 and dgLCO1490 + dgHCO2198 where excluded for calculating the linear regression.
