# Supplementary material for: Validation of COI metabarcoding primers for terrestrial arthropods
Source: PeerJ. 2019 Oct 7;7:e7745. doi: 10.7717/peerj.7745 (PMC6786254; doi:10.7717/peerj.7745)
Supplement: Figure S10 [file peerj-07-7745-s010.pdf]

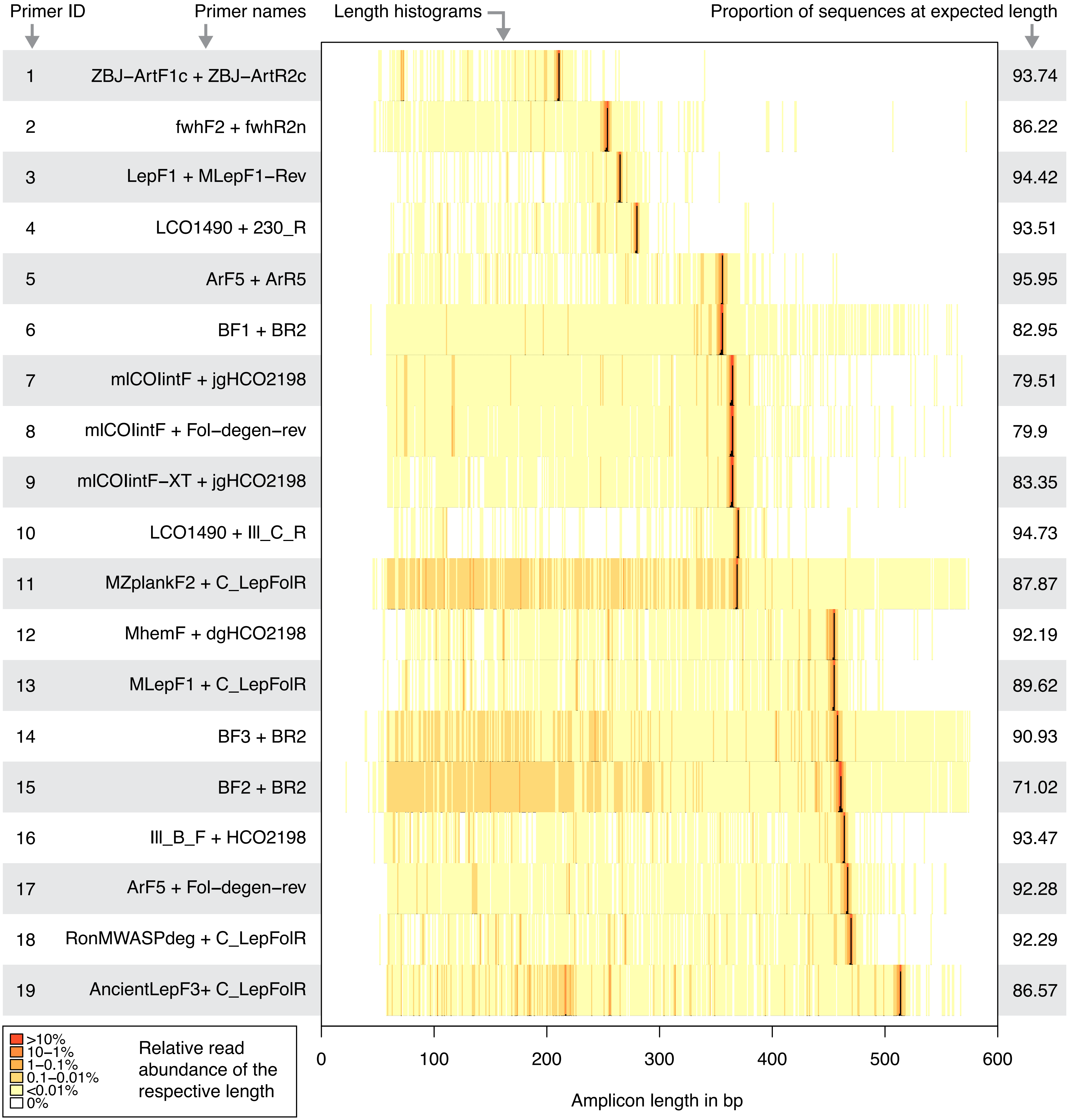

Figure S10: Histograms showing read length for 19 tested primer sets with the mock sample. Readlength calculated after paired end merging (no primer trimming, 46°C annealing temperature). Primer set 20 (LCO1490 + HCO2198) and 21 (dgLCO1490 + dgHCO2198) are not shown, since paired end merging was not possible due to the length of the full "Folmer" region. The number on the right indicates the proportion of sequences that have the exact expected amplicon length. In addition to black bars in the histogram, relative read abundance is also indicated with different shading.
