# Supplementary material for: Validation of COI metabarcoding primers for terrestrial arthropods
Source: PeerJ. 2019 Oct 7;7:e7745. doi: 10.7717/peerj.7745 (PMC6786254; doi:10.7717/peerj.7745)
Supplement: Figure S12 [file peerj-07-7745-s012.pdf]

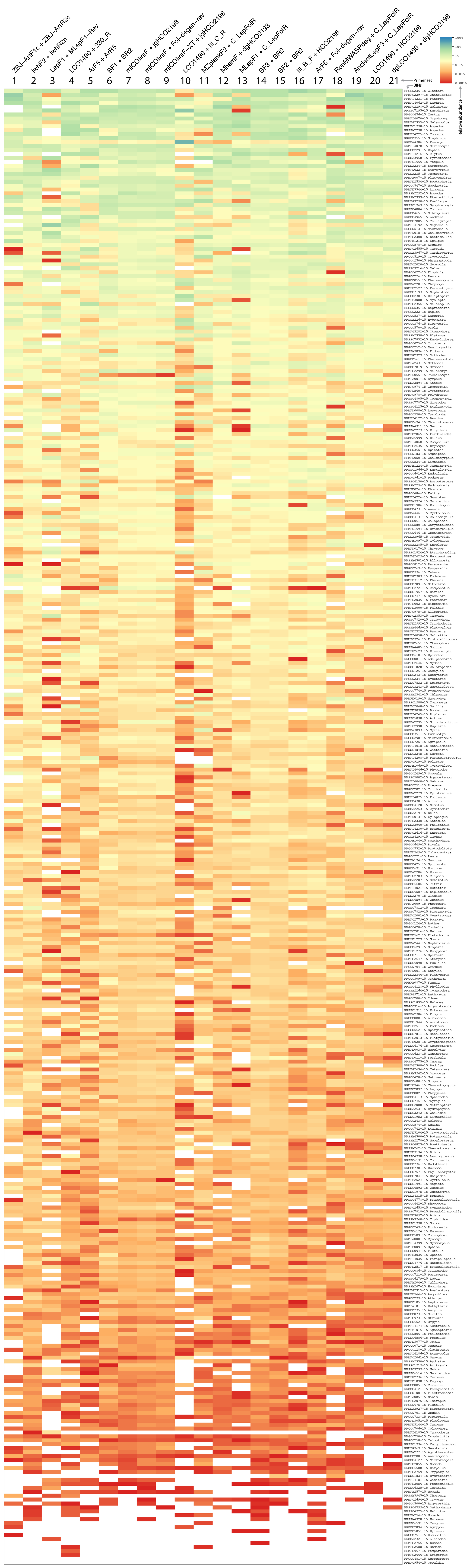


**Figure S12:** Heatmap indicating the relative read abundance for the mock sample metabarcoded with 21 different primer sets. Reads are subsampled to 100.000 reads per sample (reads with abundance below 0.001% where discarded). BINs are sorted by average relative abundance. See Table S1 for the raw data.
