# Supplementary material for: Validation of COI metabarcoding primers for terrestrial arthropods
Source: PeerJ. 2019 Oct 7;7:e7745. doi: 10.7717/peerj.7745 (PMC6786254; doi:10.7717/peerj.7745)
Supplement: Figure S13 [file peerj-07-7745-s013.pdf]

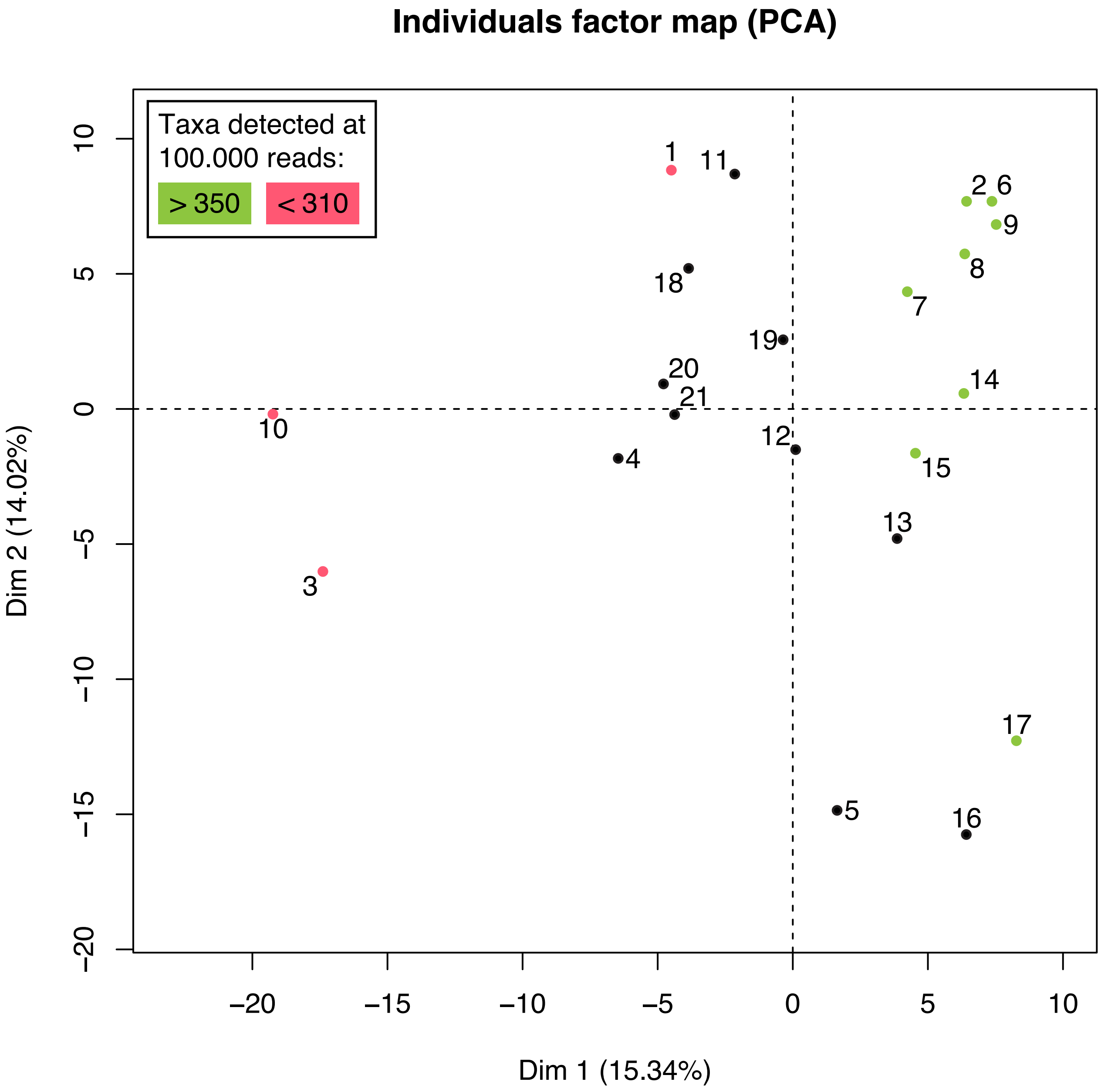

Figure S13: Pcrincipal Component analysis for the mock sample metabarcoded with 21 primer combinations. Sequencing depth was normalised to 100.000 reads for each primer sets (reads below 0.001% abundance discarded).
