# Supplementary material for: Validation of COI metabarcoding primers for terrestrial arthropods
Source: PeerJ. 2019 Oct 7;7:e7745. doi: 10.7717/peerj.7745 (PMC6786254; doi:10.7717/peerj.7745)
Supplement: Figure S14 [file peerj-07-7745-s014.pdf]

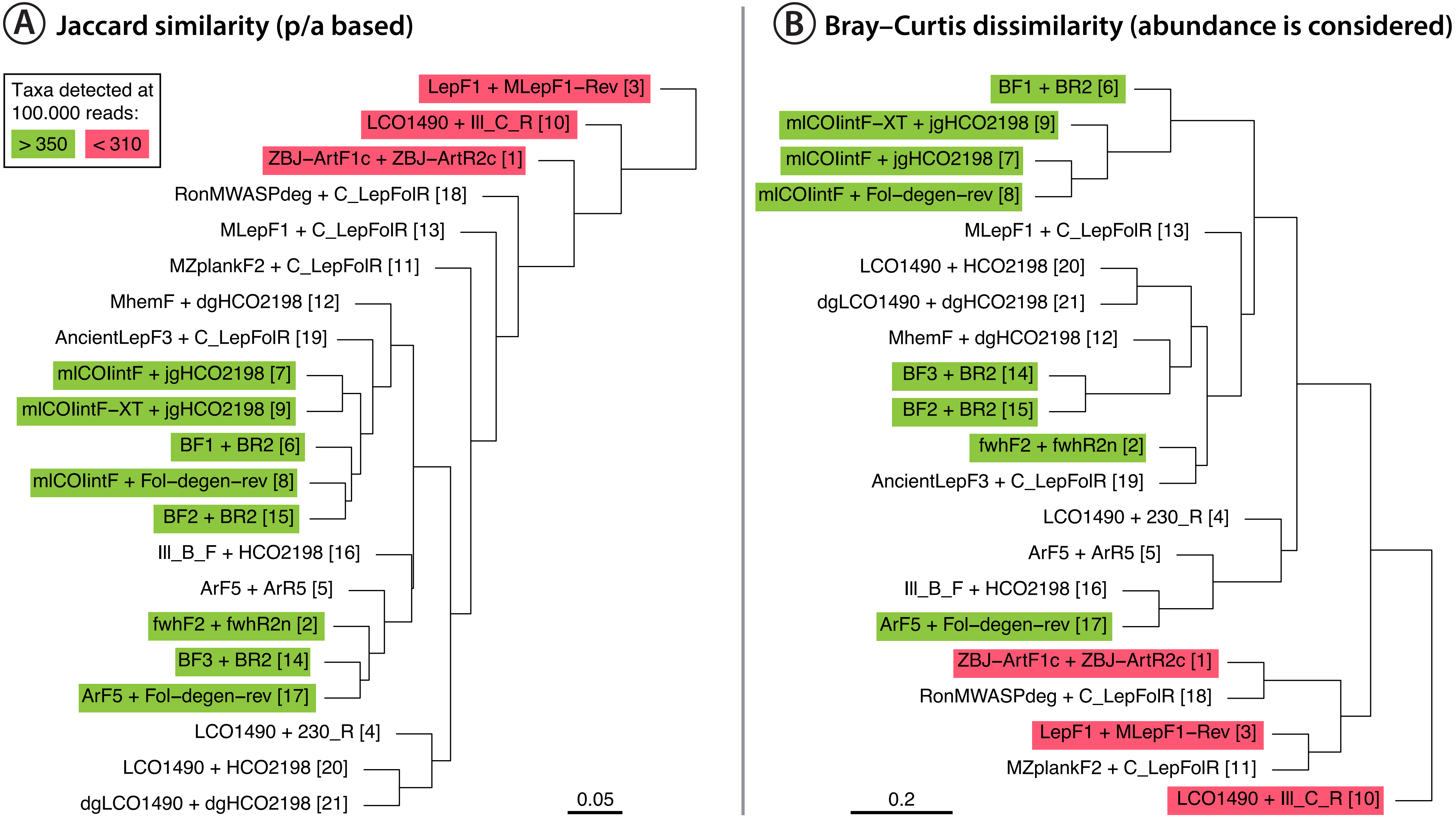

Figure S14: Cluster Dendrogram (UPGMA) for Jaccard similarity (A) and Bray–Curtis dissimilarity (B) for the mock sample metabarcoding data using 21 primer sets, generated with the R package veagan v2.5-2. If the number of taxa detected is above 350 or below 310 taxa, the primer set is highlighted in Green or Red respectively. Sequencing depth was normalised to 100.000 reads for each primer sets (reads below 0.001% abundance discarded).
