# Supplementary material for: Validation of COI metabarcoding primers for terrestrial arthropods
Source: PeerJ. 2019 Oct 7;7:e7745. doi: 10.7717/peerj.7745 (PMC6786254; doi:10.7717/peerj.7745)
Supplement: Figure S15 [file peerj-07-7745-s015.pdf]

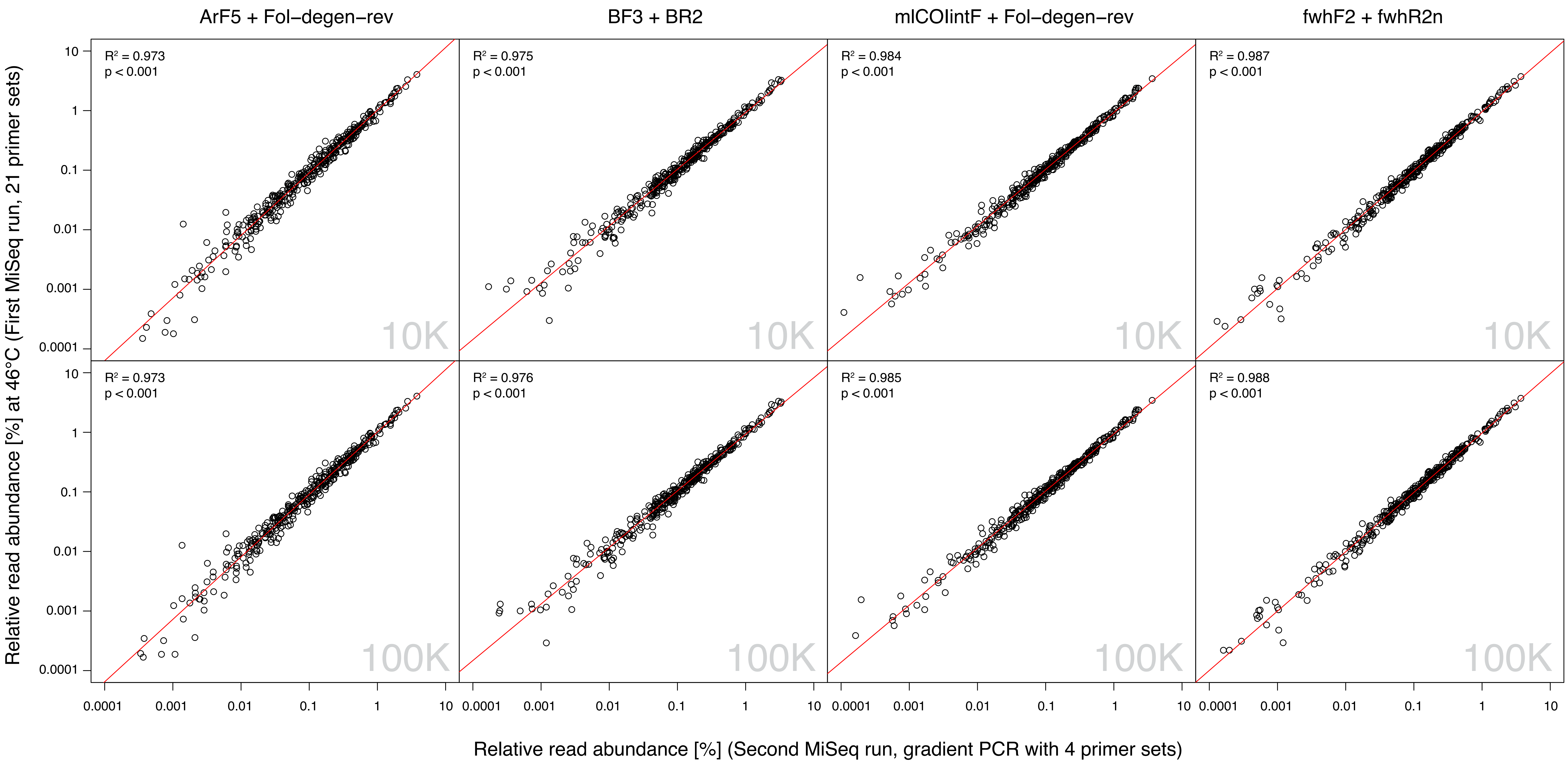


**Figure S15:** Comparison of read abundances of the 4 primer sets tested in both the mock sample primer test and gredient PCR run (both at 46°C). Sequencing depth was subsamples to 10.000 or 100.000 sequences with 1000 replicates. The red line indicates the linear regression between both samples.
