# Supplementary material for: Validation of COI metabarcoding primers for terrestrial arthropods
Source: PeerJ. 2019 Oct 7;7:e7745. doi: 10.7717/peerj.7745 (PMC6786254; doi:10.7717/peerj.7745)
Supplement: Figure S16 [file peerj-07-7745-s016.pdf]

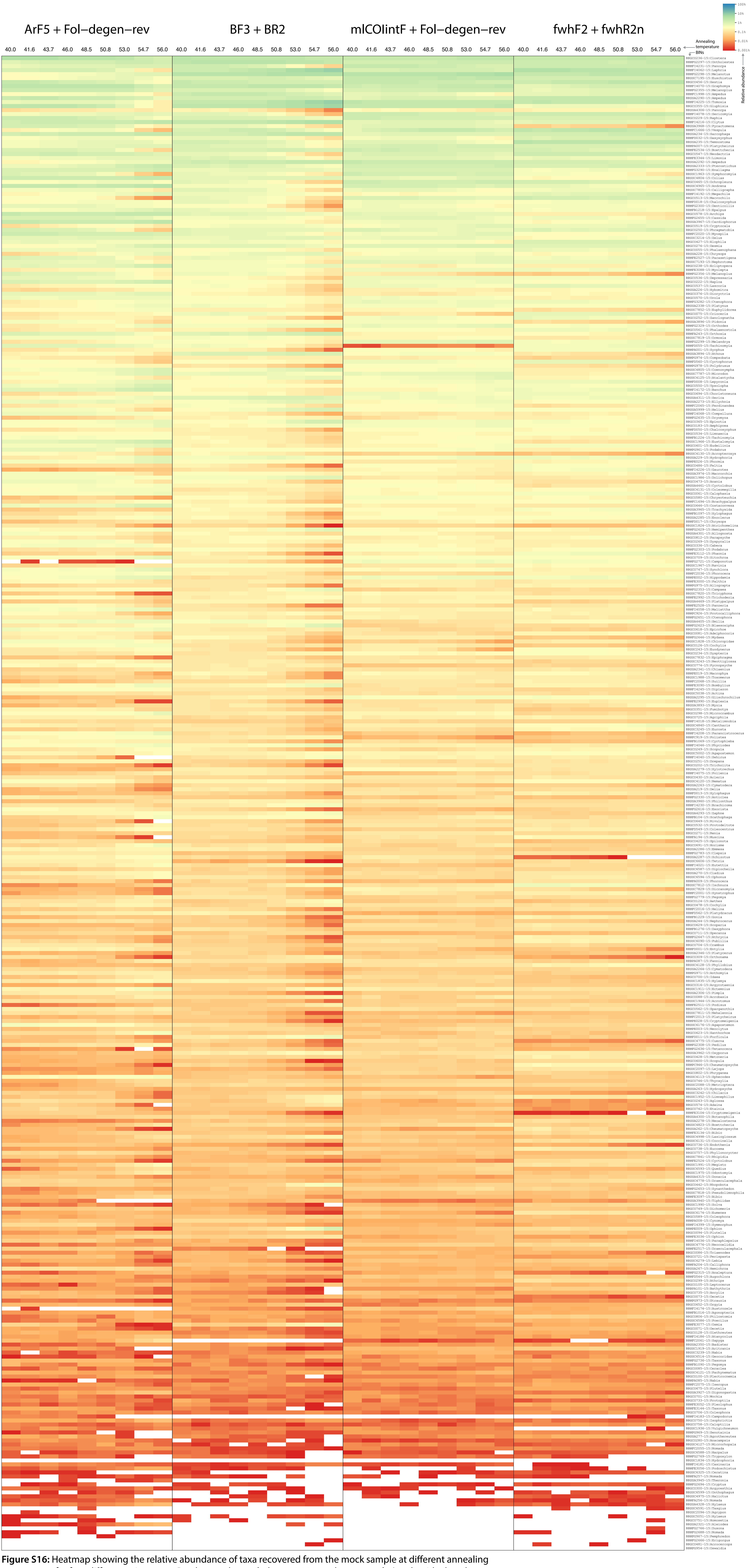

Figure S16: Heatmap showing the relative abundance of taxa recovered from the mock sample at different annealing tempratures for four different primer sets. Reads are subsampled to 100.000 reads per sample (reads with abundance below 0.001% where discarded). BINs are sorted the same way as in Figure SXXX. See Table S2 for the raw data.
