# Supplementary material for: Validation of COI metabarcoding primers for terrestrial arthropods
Source: PeerJ. 2019 Oct 7;7:e7745. doi: 10.7717/peerj.7745 (PMC6786254; doi:10.7717/peerj.7745)
Supplement: Figure S17 [file peerj-07-7745-s017.pdf]

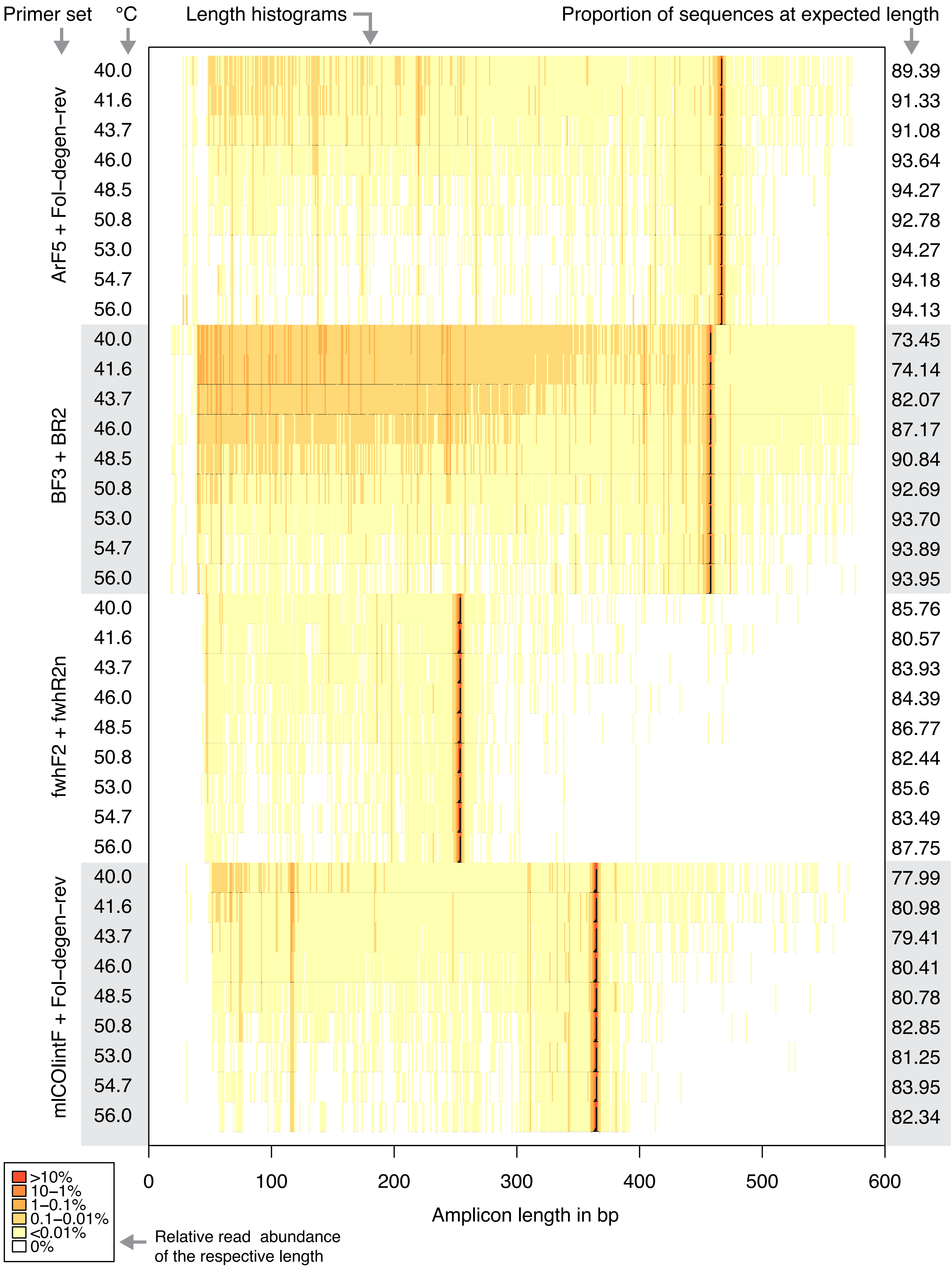

Figure S17: Histograms showing read length for four primer sets amplifing the mock sample in a gradient PCR (no primer trimming, 40-56°C). The number on the right indicates the proportion of sequences that have the exact expected amplicon length. In addition to black bars in the histogram, relative read abundance is also indicated with different shading.
