# Supplementary material for: Validation of COI metabarcoding primers for terrestrial arthropods
Source: PeerJ. 2019 Oct 7;7:e7745. doi: 10.7717/peerj.7745 (PMC6786254; doi:10.7717/peerj.7745)
Supplement: Figure S19 [file peerj-07-7745-s019.pdf]

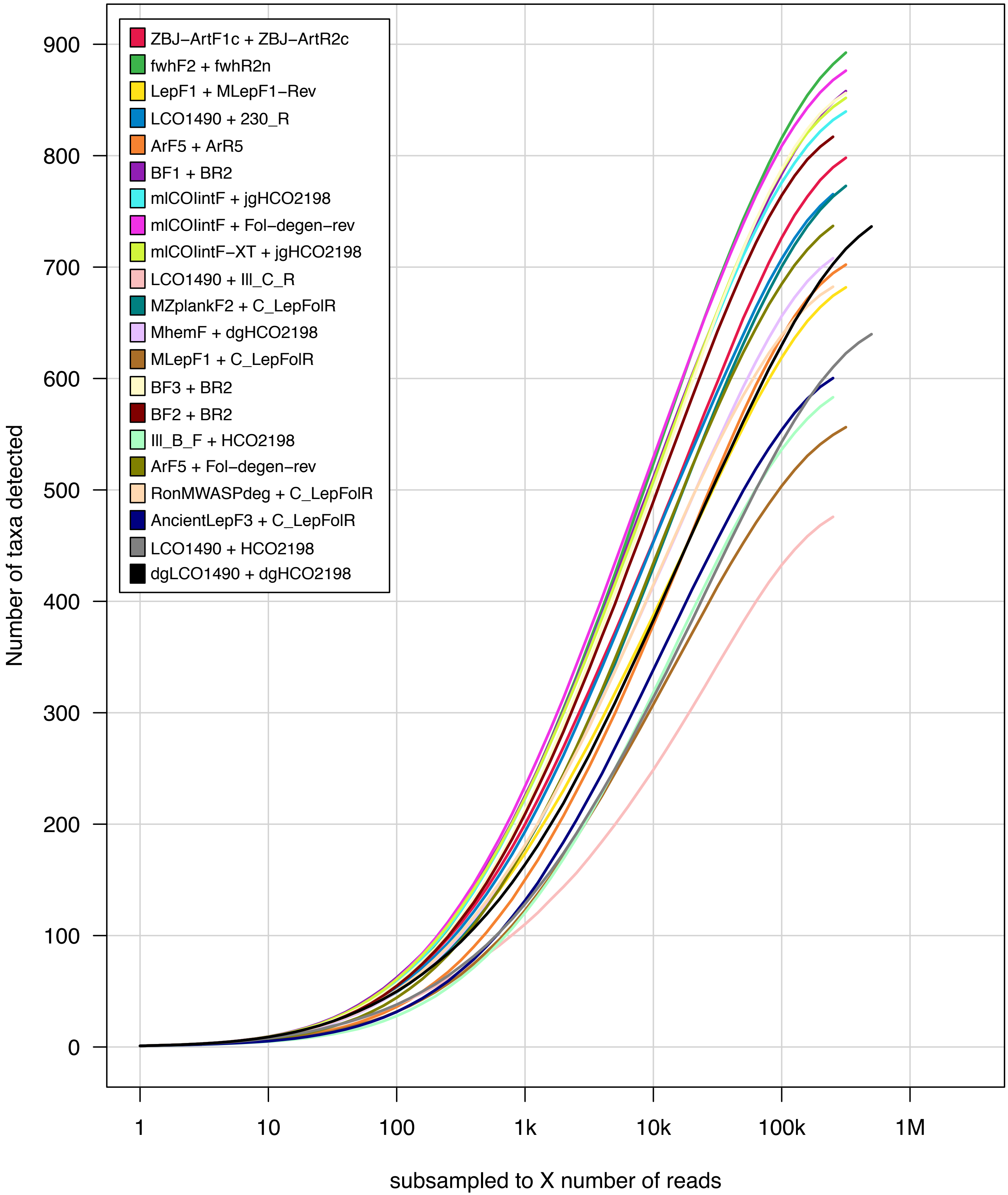

Figure S19: Rarefaction curve showing the amount of taxa recovered from the malaise trap sample at different sequencing depth for all 21 primer sets. For each data point 1000 subsamples where taken.
