# Supplementary material for: Validation of COI metabarcoding primers for terrestrial arthropods
Source: PeerJ. 2019 Oct 7;7:e7745. doi: 10.7717/peerj.7745 (PMC6786254; doi:10.7717/peerj.7745)
Supplement: Supplemental Information 1 [file peerj-07-7745-s020.zip › Scripts_1_v2/R_scripts/fusion primers/SXX 4 gradient primers/223_Hamming.pdf]

	2) fwhF2_GCACTGG	2B) fwhF2_TGATGGG	2C) fwhF2_CGGGGGA	2C) fwhF2_CGGGGGG	2C) fwhF2_CGGGGGT	8) mlCOIintF_ATTACAC	8B) mlCOIintF_GCGTATG	8C) mlCOIintF_ATTTAGG	14) BF3_CTTCCCC	14B) BF3_AACGCCA	14B) BF3_AACGCCC	14B) BF3_AACGCCT	14C) BF3_GCAAACC	17) ArF5_GTCCTGC	17B) ArF5_ATTGGAA	17C) ArF5_CAATAAG	2) fwhR2n_GACATGT	2B) fwhR2n_GATTCCT	2C) fwhR2n_CGCCGTA	2C) fwhR2n_CGCCGTG	Fol-degen-rev_ACAGCTA	Fol-degen-rev_TCGCTAA	Fol-degen-rev_TCGCTAC	Fol-degen-rev_TCGCTAG	Fol-degen-rev_TCGCTAT	Fol-degen-rev_AGGTGCT	15) BR2_TGCGGTT	14A) BR2_CTCCATC	14B) BR2_CGGATCA	14B) BR2_CGGATCG	14B) BR2_CGGATCT	Fol-degen-rev_TAGACTA	Fol-degen-rev_TCAGAAT	Fol-degen-rev_TATATAA	Fol-degen-rev_TATATAC	Fol-degen-rev_TATATAG	Fol-degen-rev_TATATAT
2) fwhF2_GCACTGG	0	4	6	5	6	7	4	5	6	7	7	7	4	3	7	5	4	6	6	5	5	4	4	3	4	7	7	6	6	5	6	7	5	6	6	5	6
2B) fwhF2_TGATGGG	4	0	4	3	4	7	5	4	7	7	7	7	6	6	6	4	6	6	5	4	6	6	6	5	6	4	4	7	6	5	6	6	5	6	6	5	6
2C) fwhF2_CGGGGGA	6	4	0	1	1	7	6	6	6	5	6	6	7	6	4	6	6	7	3	4	5	5	6	6	6	4	4	6	3	4	4	5	6	6	7	7	7
2C) fwhF2_CGGGGGG	5	3	1	0	1	7	5	5	6	6	6	6	7	6	5	5	6	7	4	3	6	6	6	5	6	4	4	6	4	3	4	6	6	7	7	6	7
2C) fwhF2_CGGGGGT	6	4	1	1	0	7	6	6	6	6	6	5	7	6	5	6	5	6	4	4	6	6	6	6	5	3	3	6	4	4	3	6	5	7	7	7	6
8) mlCOIintF_ATTACAC	7	7	7	7	7	0	7	4	3	5	4	5	5	5	3	6	6	5	7	7	5	6	5	6	6	6	7	5	6	6	6	5	6	4	3	4	4
8B) mlCOIintF_GCGTATG	4	5	6	5	6	7	0	4	7	7	7	7	4	6	7	4	6	5	6	5	5	5	5	4	5	5	6	5	6	5	6	5	5	7	7	6	7
8C) mlCOIintF_ATTTAGG	5	4	6	5	6	4	4	0	5	6	6	6	6	5	4	4	6	5	7	6	6	7	7	6	7	5	7	5	7	6	7	7	6	6	6	5	6
14) BF3_CTTCCCC	6	7	6	6	6	3	7	5	0	5	4	5	5	4	5	6	7	4	5	5	6	6	5	6	6	6	7	3	5	5	5	6	7	6	5	6	6
14B) BF3_AACGCCA	7	7	5	6	6	5	7	6	5	0	1	1	6	6	4	6	5	4	5	6	3	6	7	7	7	5	5	6	5	6	6	4	6	5	6	6	6
14B) BF3_AACGCCC	7	7	6	6	6	4	7	6	4	1	0	1	5	5	5	6	5	4	6	6	4	7	6	7	7	5	5	5	6	6	6	5	6	6	5	6	6
14B) BF3_AACGCCT	7	7	6	6	5	5	7	6	5	1	1	0	6	6	5	6	4	3	6	6	4	7	7	7	6	4	4	6	6	6	5	5	5	6	6	6	5
14C) BF3_GCAAACC	4	6	7	7	7	5	4	6	5	6	5	6	0	5	7	5	5	5	7	7	5	6	5	6	6	6	7	5	5	5	5	6	4	6	5	6	6
17) ArF5_GTCCTGC	3	6	6	6	6	5	6	5	4	6	5	6	5	0	6	7	3	6	5	5	7	5	4	5	5	7	6	3	6	6	6	7	7	6	5	6	6
17B) ArF5_ATTGGAA	7	6	4	5	5	3	7	4	5	4	5	5	7	6	0	6	7	6	5	6	4	5	6	6	6	5	5	6	6	7	7	6	5	4	5	5	5
17C) ArF5_CAATAAG	5	4	6	5	6	6	4	4	6	6	6	6	5	7	6	0	6	5	6	5	6	6	6	5	6	6	7	5	6	5	6	6	4	5	5	4	5
2) fwhR2n_GACATGT	4	6	6	6	5	6	6	6	7	5	5	4	5	3	7	6	0	4	6	6	7	6	6	6	5	6	5	6	5	5	4	5	6	4	4	4	3
2B) fwhR2n_GATTCCT	6	6	7	7	6	5	5	5	4	4	4	3	5	6	6	5	4	0	7	7	6	7	7	7	6	4	6	7	6	6	5	5	6	5	5	5	4
2C) fwhR2n_CGCCGTA	6	5	3	4	4	7	6	7	5	5	6	6	7	5	5	6	6	7	0	1	5	5	6	6	6	5	3	3	4	5	5	5	7	6	7	7	7
2C) fwhR2n_CGCCGTG	5	4	4	3	4	7	5	6	5	6	6	6	7	5	6	5	6	7	1	0	6	6	6	5	6	5	3	3	5	4	5	6	7	7	7	6	7
Fol-degen-rev_ACAGCTA	5	6	5	6	6	5	5	6	6	3	4	4	5	7	4	6	7	6	5	6	0	5	6	6	6	6	5	6	6	7	7	4	4	6	7	7	7
Fol-degen-rev_TCGCTAA	4	6	5	6	6	6	5	7	6	6	7	7	6	5	5	6	6	7	5	6	5	0	1	1	1	6	6	6	4	5	5	4	4	3	4	4	4
Fol-degen-rev_TCGCTAC	4	6	6	6	6	5	5	7	5	7	6	7	5	4	6	6	6	7	6	6	6	1	0	1	1	6	6	5	5	5	5	5	4	4	3	4	4
Fol-degen-rev_TCGCTAG	3	5	6	5	6	6	4	6	6	7	7	7	6	5	6	5	6	7	6	5	6	1	1	0	1	6	6	6	5	4	5	5	4	4	4	3	4
Fol-degen-rev_TCGCTAT	4	6	6	6	5	6	5	7	6	7	7	6	6	5	6	6	5	6	6	6	6	1	1	1	0	5	5	6	5	5	4	5	3	4	4	4	3
Fol-degen-rev_AGGTGCT	7	4	4	4	3	6	5	5	6	5	5	4	6	7	5	6	6	4	5	5	6	6	6	6	5	0	4	7	4	4	3	6	6	7	7	7	6
15) BR2_TGCGGTT	7	4	4	4	3	7	6	7	7	5	5	4	7	6	5	7	5	6	3	3	5	6	6	6	5	4	0	5	6	6	5	5	4	6	6	6	5
14A) BR2_CTCCATC	6	7	6	6	6	5	5	5	3	6	5	6	5	3	6	5	6	7	3	3	6	6	5	6	6	7	5	0	6	6	6	6	6	7	6	7	7
14B) BR2_CGGATCA	6	6	3	4	4	6	6	7	5	5	6	6	5	6	6	6	5	6	4	5	6	4	5	5	5	4	6	6	0	1	1	4	7	4	5	5	5
14B) BR2_CGGATCG	5	5	4	3	4	6	5	6	5	6	6	6	5	6	7	5	5	6	5	4	7	5	5	4	5	4	6	6	1	0	1	5	7	5	5	4	5
14B) BR2_CGGATCT	6	6	4	4	3	6	6	7	5	6	6	5	5	6	7	6	4	5	5	5	7	5	5	5	4	3	5	6	1	1	0	5	6	5	5	5	4
Fol-degen-rev_TAGACTA	7	6	5	6	6	5	5	7	6	4	5	5	6	7	6	6	5	5	5	6	4	4	5	5	5	6	5	6	4	5	5	0	6	3	4	4	4
Fol-degen-rev_TCAGAAT	5	5	6	6	5	6	5	6	7	6	6	5	4	7	5	4	6	6	7	7	4	4	4	4	3	6	4	6	7	7	6	6	0	5	5	5	4
Fol-degen-rev_TATATAA	6	6	6	7	7	4	7	6	6	5	6	6	6	6	4	5	4	5	6	7	6	3	4	4	4	7	6	7	4	5	5	3	5	0	1	1	1
Fol-degen-rev_TATATAC	6	6	7	7	7	3	7	6	5	6	5	6	5	5	5	5	4	5	7	7	7	4	3	4	4	7	6	6	5	5	5	4	5	1	0	1	1
Fol-degen-rev_TATATAG	5	5	7	6	7	4	6	5	6	6	6	6	6	6	5	4	4	5	7	6	7	4	4	3	4	7	6	7	5	4	5	4	5	1	1	0	1
Fol-degen-rev_TATATAT	6	6	7	7	6	4	7	6	6	6	6	5	6	6	5	5	3	4	7	7	7	4	4	4	3	6	5	7	5	5	4	4	4	1	1	1	0
