# Supplementary material for: Validation of COI metabarcoding primers for terrestrial arthropods
Source: PeerJ. 2019 Oct 7;7:e7745. doi: 10.7717/peerj.7745 (PMC6786254; doi:10.7717/peerj.7745)
Supplement: Supplemental Information 1 [file peerj-07-7745-s020.zip › Scripts_1_v2/R_scripts/fusion primers/SXX 4 gradient primers/223_alignment.pdf]

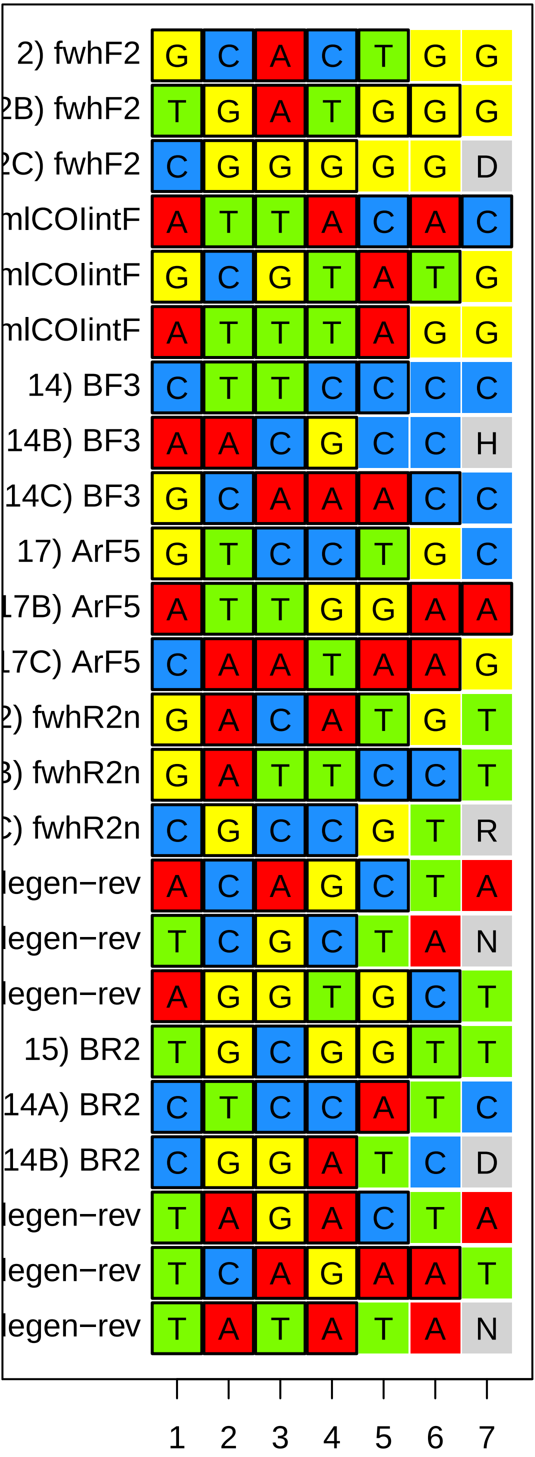

2) fwhF2 G C A C T G G
B) fwhF2 T G A T G G G
C) fwhF2 C G G G G G D
mlCOIintF A T T A C A C
mlCOIintF G C G T A T G
mlCOIintF A T T T A G G
14) BF3 C T T C C C C
14B) BF3 A A C G C C H
14C) BF3 G C A A A C C
17) ArF5 G T C C T G C
7B) ArF5 A T T G G A A
7C) ArF5 C A A T A A G
) fwhR2n G A C A T G T
) fwhR2n G A T T C C T
) fwhR2n C G C C G T R
legen−rev A C A G C T A
legen−rev T C G C T A N
legen−rev A G G T G C T
15) BR2 T G C G G T T
14A) BR2 C T C C A T C
14B) BR2 C G G A T C D
legen−rev T A G A C T A
legen−rev T C A G A A T
legen−rev T A T A T A N
1 2 3 4 5 6 7
