# Supplementary material for: Validation of COI metabarcoding primers for terrestrial arthropods
Source: PeerJ. 2019 Oct 7;7:e7745. doi: 10.7717/peerj.7745 (PMC6786254; doi:10.7717/peerj.7745)
Supplement: Supplemental Information 1 [file peerj-07-7745-s020.zip › Scripts_1_v2/R_scripts/fusion primers/SXX 4 gradient primers/223_alignment copy.pdf]

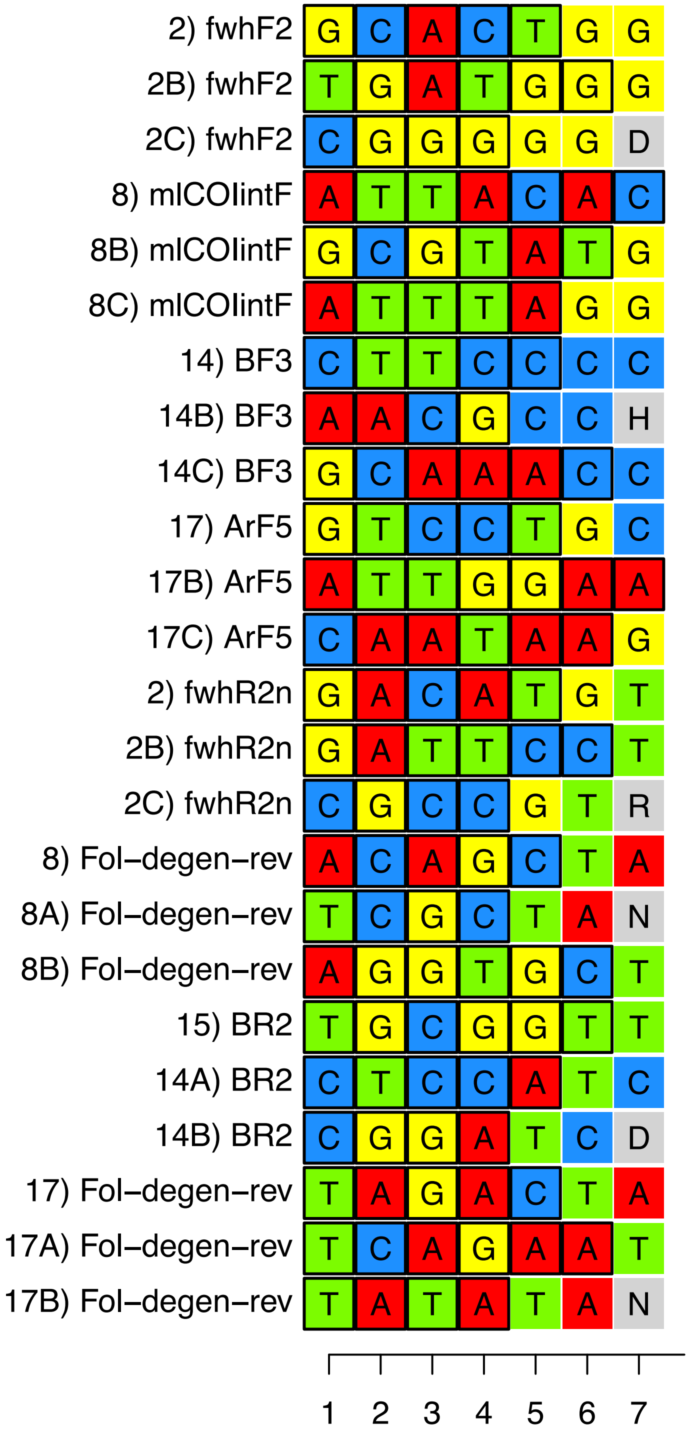
2) fwhF2 G C A C T G G
2B) fwhF2 T G A T G G G
2C) fwhF2 C G G G G G D
8) mlCOIintF A T T A C A C
8B) mlCOIintF G C G T A T G
8C) mlCOIintF A T T T A G G
14) BF3 C T T C C C C
14B) BF3 A A C G C C H
14C) BF3 G C A A A C C
17) ArF5 G T C C T G C
17B) ArF5 A T T G G A A
17C) ArF5 C A A T A A G
2) fwhR2n G A C A T G T
2B) fwhR2n G A T T C C T
2C) fwhR2n C G C C G T R
8) Fol–degen–rev A C A G C T A
8A) Fol–degen–rev T C G C T A N
8B) Fol–degen–rev A G G T G C T
15) BR2 T G C G G T T
14A) BR2 C T C C A T C
14B) BR2 C G G A T C D
17) Fol–degen–rev T A G A C T A
17A) Fol–degen–rev T C A G A A T
17B) Fol–degen–rev T A T A T A N
1 2 3 4 5 6 7
