# Supplementary material for: Validation of COI metabarcoding primers for terrestrial arthropods
Source: PeerJ. 2019 Oct 7;7:e7745. doi: 10.7717/peerj.7745 (PMC6786254; doi:10.7717/peerj.7745)
Supplement: Supplemental Information 1 [file peerj-07-7745-s020.zip › Scripts_1_v2/R_scripts/fusion primers/SXX 4 gradient primers/223_Levenshtein.pdf]

	2) fwhF2_GCACTGG	2B) fwhF2_TGATGGG	2C) fwhF2_CGGGGGA	2C) fwhF2_CGGGGGG	2C) fwhF2_CGGGGGT	8) mlCOIintF_ATTACAC	8B) mlCOIintF_GCGTATG	8C) mlCOIintF_ATTTAGG	14) BF3_CTTCCCC	14B) BF3_AACGCCA	14B) BF3_AACGCCC	14B) BF3_AACGCCT	14C) BF3_GCAAACC	17) ArF5_GTCCTGC	17B) ArF5_ATTGGAA	17C) ArF5_CAATAAG	2) fwhR2n_GACATGT	2B) fwhR2n_GATTCCT	2C) fwhR2n_CGCCGTA	2C) fwhR2n_CGCCGTG	Fol-degen-rev_ACAGCTA	Fol-degen-rev_TCGCTAA	Fol-degen-rev_TCGCTAC	Fol-degen-rev_TCGCTAG	Fol-degen-rev_TCGCTAT	Fol-degen-rev_AGGTGCT	15) BR2_TGCGGTT	14A) BR2_CTCCATC	14B) BR2_CGGATCA	14B) BR2_CGGATCG	14B) BR2_CGGATCT	Fol-degen-rev_TAGACTA	Fol-degen-rev_TCAGAAT	Fol-degen-rev_TATATAA	Fol-degen-rev_TATATAC	Fol-degen-rev_TATATAG	Fol-degen-rev_TATATAT
2) fwhF2_GCACTGG	0	4	5	5	5	6	4	5	6	6	6	6	4	3	5	4	3	5	5	4	4	4	4	3	4	6	5	6	5	4	5	5	5	6	6	5	6
2B) fwhF2_TGATGGG	4	0	4	3	4	6	5	4	7	6	6	6	6	5	5	4	5	5	5	4	6	5	5	4	5	4	4	6	5	4	5	5	5	5	5	4	5
2C) fwhF2_CGGGGGA	5	4	0	1	1	7	5	6	6	5	6	6	7	6	4	6	6	7	3	4	5	5	6	5	6	4	4	6	3	4	4	5	6	6	7	7	7
2C) fwhF2_CGGGGGG	5	3	1	0	1	7	5	5	6	6	6	6	7	6	5	5	6	7	4	3	6	6	6	5	6	4	4	6	4	3	4	6	6	7	7	6	7
2C) fwhF2_CGGGGGT	5	4	1	1	0	7	5	6	6	6	6	5	7	6	5	6	5	6	4	4	6	6	6	5	5	3	3	6	4	4	3	6	5	7	7	7	6
8) mlCOIintF_ATTACAC	6	6	7	7	7	0	6	4	3	5	4	5	5	5	3	4	6	4	7	7	5	5	4	5	5	5	7	4	5	6	6	5	5	4	3	4	4
8B) mlCOIintF_GCGTATG	4	5	5	5	5	6	0	4	6	6	6	5	4	5	6	4	4	5	4	4	5	4	4	3	3	5	4	5	4	3	4	5	4	5	5	4	5
8C) mlCOIintF_ATTTAGG	5	4	6	5	6	4	4	0	5	6	6	6	6	5	4	4	5	5	7	6	6	6	6	5	6	5	6	5	7	6	7	6	6	4	4	3	4
14) BF3_CTTCCCC	6	7	6	6	6	3	6	5	0	5	4	5	5	4	5	6	7	4	5	5	6	6	5	6	6	6	6	3	5	5	5	6	7	6	5	6	6
14B) BF3_AACGCCA	6	6	5	6	6	5	6	6	5	0	1	1	5	6	4	6	5	4	4	5	3	4	4	4	4	5	5	5	5	6	6	4	6	5	6	6	6
14B) BF3_AACGCCC	6	6	6	6	6	4	6	6	4	1	0	1	5	5	5	6	5	4	5	5	4	5	4	5	5	5	5	5	6	6	6	5	6	6	5	6	6
14B) BF3_AACGCCT	6	6	6	6	5	5	5	6	5	1	1	0	5	6	5	6	4	3	4	4	4	5	5	5	4	4	4	5	6	6	5	4	5	6	6	6	5
14C) BF3_GCAAACC	4	6	7	7	7	5	4	6	5	5	5	5	0	5	7	4	5	4	6	6	5	5	5	6	6	6	6	5	5	5	5	5	4	6	5	6	6
17) ArF5_GTCCTGC	3	5	6	6	6	5	5	5	4	6	5	6	5	0	6	7	3	4	5	4	6	4	3	4	4	5	5	3	6	6	6	6	6	6	5	6	6
17B) ArF5_ATTGGAA	5	5	4	5	5	3	6	4	5	4	5	5	7	6	0	5	6	5	5	6	4	4	5	5	5	5	5	6	5	6	6	5	4	4	5	5	5
17C) ArF5_CAATAAG	4	4	6	5	6	4	4	4	6	6	6	6	4	7	5	0	5	5	6	5	5	4	5	4	5	6	7	5	4	4	5	5	4	3	4	3	4
2) fwhR2n_GACATGT	3	5	6	6	5	6	4	5	7	5	5	4	5	3	6	5	0	4	5	4	4	6	6	6	5	4	5	5	5	5	4	5	5	4	4	4	3
2B) fwhR2n_GATTCCT	5	5	7	7	6	4	5	5	4	4	4	3	4	4	5	5	4	0	6	6	5	6	6	6	6	4	6	5	5	5	4	5	6	5	4	5	4
2C) fwhR2n_CGCCGTA	5	5	3	4	4	7	4	7	5	4	5	4	6	5	5	6	5	6	0	1	4	4	4	4	4	5	3	3	4	5	5	4	5	6	6	6	6
2C) fwhR2n_CGCCGTG	4	4	4	3	4	7	4	6	5	5	5	4	6	4	6	5	4	6	1	0	5	5	5	4	4	5	3	3	5	4	5	5	6	7	7	6	7
Fol-degen-rev_ACAGCTA	4	6	5	6	6	5	5	6	6	3	4	4	5	6	4	5	4	5	4	5	0	3	3	3	3	4	5	5	4	5	5	3	4	5	5	5	5
Fol-degen-rev_TCGCTAA	4	5	5	6	6	5	4	6	6	4	5	5	5	4	4	4	6	6	4	5	3	0	1	1	1	6	4	5	4	5	5	3	4	3	4	4	4
Fol-degen-rev_TCGCTAC	4	5	6	6	6	4	4	6	5	4	4	5	5	3	5	5	6	6	4	5	3	1	0	1	1	5	4	4	5	5	5	3	4	4	3	4	4
Fol-degen-rev_TCGCTAG	3	4	5	5	5	5	3	5	6	4	5	5	6	4	5	4	6	6	4	4	3	1	1	0	1	6	4	5	5	4	5	3	4	4	4	3	4
Fol-degen-rev_TCGCTAT	4	5	6	6	5	5	3	6	6	4	5	4	6	4	5	5	5	6	4	4	3	1	1	1	0	5	3	4	5	5	4	3	3	4	4	4	3
Fol-degen-rev_AGGTGCT	6	4	4	4	3	5	5	5	6	5	5	4	6	5	5	6	4	4	5	5	4	6	5	6	5	0	4	6	4	4	3	5	6	6	5	6	5
15) BR2_TGCGGTT	5	4	4	4	3	7	4	6	6	5	5	4	6	5	5	7	5	6	3	3	5	4	4	4	3	4	0	5	5	5	4	5	4	6	6	6	5
14A) BR2_CTCCATC	6	6	6	6	6	4	5	5	3	5	5	5	5	3	6	5	5	5	3	3	5	5	4	5	4	6	5	0	4	4	4	5	5	5	4	5	5
14B) BR2_CGGATCA	5	5	3	4	4	5	4	7	5	5	6	6	5	6	5	4	5	5	4	5	4	4	5	5	5	4	5	4	0	1	1	4	5	4	5	5	5
14B) BR2_CGGATCG	4	4	4	3	4	6	3	6	5	6	6	6	5	6	6	4	5	5	5	4	5	5	5	4	5	4	5	4	1	0	1	5	5	5	5	4	5
14B) BR2_CGGATCT	5	5	4	4	3	6	4	7	5	6	6	5	5	6	6	5	4	4	5	5	5	5	5	5	4	3	4	4	1	1	0	4	4	5	5	5	4
Fol-degen-rev_TAGACTA	5	5	5	6	6	5	5	6	6	4	5	4	5	6	5	5	5	5	4	5	3	3	3	3	3	5	5	5	4	5	4	0	3	3	3	3	3
Fol-degen-rev_TCAGAAT	5	5	6	6	5	5	4	6	7	6	6	5	4	6	4	4	5	6	5	6	4	4	4	4	3	6	4	5	5	5	4	3	0	4	4	4	3
Fol-degen-rev_TATATAA	6	5	6	7	7	4	5	4	6	5	6	6	6	6	4	3	4	5	6	7	5	3	4	4	4	6	6	5	4	5	5	3	4	0	1	1	1
Fol-degen-rev_TATATAC	6	5	7	7	7	3	5	4	5	6	5	6	5	5	5	4	4	4	6	7	5	4	3	4	4	5	6	4	5	5	5	3	4	1	0	1	1
Fol-degen-rev_TATATAG	5	4	7	6	7	4	4	3	6	6	6	6	6	6	5	3	4	5	6	6	5	4	4	3	4	6	6	5	5	4	5	3	4	1	1	0	1
Fol-degen-rev_TATATAT	6	5	7	7	6	4	5	4	6	6	6	5	6	6	5	4	3	4	6	7	5	4	4	4	3	5	5	5	5	5	4	3	3	1	1	1	0
