# Supplementary material for: Validation of COI metabarcoding primers for terrestrial arthropods
Source: PeerJ. 2019 Oct 7;7:e7745. doi: 10.7717/peerj.7745 (PMC6786254; doi:10.7717/peerj.7745)
Supplement: Supplemental Information 1 [file peerj-07-7745-s020.zip › Scripts_1_v2/R_scripts/fusion primers/SXX 21 primer pairs/alignment_v1 copy.pdf]

Primer set	No.	Primer								insert length in bp
Forward primers	1)	ZBJ–ArtF1c	G	G	A	C	A	G	A	4
	2)	fwhF2	G	C	A	C	T	G	G	5
	3)	LepF1	C	G	T	C	G	C	A	7
	4)	LCO1490	A	A	G	T	G	G	G	5
	5)	ArF5	T	G	G	T	C	G	C	5
	6)	BF1	C	T	C	C	G	A	C	5
	7)	mlCOIintF	T	G	C	T	T	G	G	5
	8)	mlCOIintF	A	T	T	A	C	A	C	7
	9)	mlCOIintF–XT	C	G	C	T	A	T	G	6
	10)	LCO1490	A	C	G	G	C	G	G	5
	11)	MZplankF2	A	A	T	T	T	A	C	7
	12)	MhemF	T	G	A	A	A	G	C	5
	13)	MLepF1	G	A	A	T	T	G	C	5
	14)	BF3	C	T	T	C	C	C	C	5
	15)	BF2	T	C	T	G	C	G	C	5
	16)	III_B_F	C	T	G	G	A	C	C	6
	17)	ArF5	G	T	C	C	T	G	C	5
	18)	RonMWASPdeg	G	A	A	G	G	C	G	6
	19)	AcientLepF3	A	G	C	T	T	T	T	5
	20)	LCO1490	G	C	C	C	G	C	A	7
	21)	dgLCO1490	A	T	T	T	T	G	G	5
Reverse primers	1)	ZBJ–ArtR2c	C	A	A	A	C	C	G	7
	2)	fwhR2n	G	A	C	A	T	G	T	5
	3)	MLepF1–Rev	T	C	T	T	A	C	G	5
	4)	230_R	A	C	G	T	C	C	T	5
	5)	ArR5	G	T	T	C	G	G	T	5
	6)	BR2	G	C	G	G	A	A	T	7
	7)	jgHCO2198	T	A	C	G	A	A	T	6
	8)	Fol–degen–rev	A	C	A	G	C	T	A	5
	9)	jgHCO2198	T	A	A	G	T	T	A	5
	10)	III_C_R	G	T	G	A	C	G	G	5
	11)	C_LepFolR	C	C	T	C	A	T	A	5
	12)	dgHCO2198	T	G	G	T	T	T	A	5
	13)	C_LepFolR	A	T	G	C	G	A	G	7
	14)	BR2	C	C	A	C	A	C	T	6
	15)	BR2	T	G	C	G	G	T	T	6
	16)	HCO2198	C	T	T	G	G	C	T	6
	17)	Fol–degen–rev	T	A	G	A	C	T	A	5
	18)	C_LepFolR	C	T	G	G	G	T	A	5
	19)	C_LepFolR	A	A	G	A	C	A	T	6
	20)	HCO2198	C	G	C	A	T	C	T	6
	21)	dgHCO2198	T	G	T	A	A	T	A	5
