# Supplementary material for: Validation of COI metabarcoding primers for terrestrial arthropods
Source: PeerJ. 2019 Oct 7;7:e7745. doi: 10.7717/peerj.7745 (PMC6786254; doi:10.7717/peerj.7745)
Supplement: Supplemental Information 1 [file peerj-07-7745-s020.zip › Scripts_1_v2/R_scripts/fusion primers/SXX 21 primer pairs/manual_alignment.pdf]

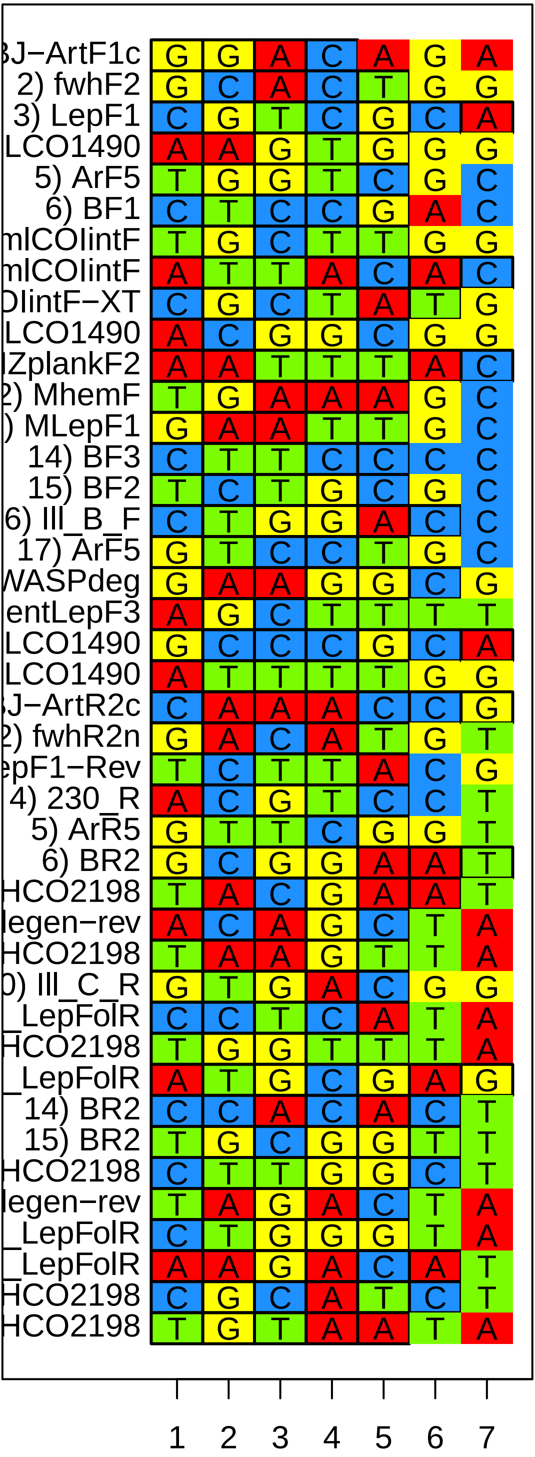
J−ArtF1c
2) fwhF2
3) LepF1
LCO1490
5) ArF5
6) BF1
mlCOlintF
mlCOlintF
OlintF−XT
LCO1490
lZplankF2
2) MhemF
) MLepF1
14) BF3
15) BF2
6) lll_B_F
17) ArF5
WASPdeg
entLepF3
LCO1490
LCO1490
J−ArtR2c
2) fwhR2n
epF1−Rev
4) 230_R
5) ArR5
6) BR2
HCO2198
legen−rev
HCO2198
0) lll_C_R
_LepFolR
HCO2198
_LepFolR
14) BR2
15) BR2
HCO2198
legen−rev
_LepFolR
_LepFolR
HCO2198
HCO2198
1
2
3
4
5
6
7
