# Supplementary material for: Validation of COI metabarcoding primers for terrestrial arthropods
Source: PeerJ. 2019 Oct 7;7:e7745. doi: 10.7717/peerj.7745 (PMC6786254; doi:10.7717/peerj.7745)
Supplement: Supplemental Information 1 [file peerj-07-7745-s020.zip › Scripts_1_v2/R_scripts/fusion primers/SXX 21 primer pairs/manual_Hamming_180705.pdf]

0	3	4	6	5	6	5	7	5	6	7	3	4	6	6	6	4	5	6	4	6	6	5	6	7	4	5	6	5	5	5	4	5	6	4	6	7	6	6	7	6	4
3	0	6	5	6	6	4	7	6	4	6	5	3	6	5	7	3	4	6	4	4	5	4	5	6	4	5	7	5	5	4	5	6	5	4	7	7	7	7	7	6	7
4	6	0	6	6	4	6	6	5	7	6	6	7	3	6	5	6	5	6	3	6	5	7	5	6	4	7	7	6	6	7	3	5	5	4	5	3	6	4	7	4	4
6	5	6	0	4	6	4	6	5	3	4	6	4	7	6	6	6	4	5	6	3	5	5	5	4	5	6	6	6	6	4	7	5	3	7	6	6	5	5	4	7	7
5	6	6	4	0	6	3	5	5	4	5	3	4	5	3	5	5	7	5	7	5	6	6	5	4	6	6	6	6	6	4	7	3	6	7	5	7	4	6	5	6	5
6	6	4	6	6	0	6	4	5	7	5	6	6	3	6	4	3	6	6	4	6	6	6	7	7	4	6	5	7	7	6	5	7	3	5	5	4	7	4	6	5	7
5	4	6	4	3	6	0	7	3	5	5	4	4	7	5	7	4	6	3	6	3	6	4	4	6	6	7	5	7	5	5	7	3	6	7	4	7	6	7	7	4	5
7	7	6	6	5	4	7	0	7	5	3	5	6	3	4	5	5	7	6	7	4	5	6	6	5	5	6	6	5	7	4	6	7	4	7	7	5	5	6	3	6	5
5	6	5	5	5	5	3	7	0	6	6	5	6	6	7	5	6	6	3	6	5	5	6	4	6	7	6	5	6	6	6	4	4	6	5	4	6	6	5	7	4	4
6	4	7	3	4	7	5	5	6	0	6	6	6	6	3	5	6	5	6	6	4	5	6	5	3	6	4	6	3	6	3	6	6	4	6	6	6	5	5	4	7	7
7	6	6	4	5	5	5	3	6	6	0	6	3	5	5	6	5	6	4	7	3	6	5	5	5	6	6	5	6	5	7	6	5	5	7	7	6	6	7	4	6	6
3	5	6	6	3	6	4	5	5	6	6	0	4	6	4	5	5	6	6	7	6	5	5	5	7	6	6	5	6	5	5	6	5	7	5	5	7	5	7	6	5	3
4	3	7	4	4	6	4	6	6	6	3	4	0	6	5	6	3	4	5	6	4	5	3	6	6	5	6	6	6	4	5	7	5	7	6	7	7	6	7	6	6	7
6	6	3	7	5	3	7	3	6	6	5	6	6	0	4	3	4	6	7	5	5	4	7	5	5	4	7	7	6	7	5	4	7	5	4	7	3	6	5	6	5	6
6	5	6	6	3	6	5	4	7	3	5	4	5	4	0	5	5	6	7	6	5	6	6	4	5	5	5	5	4	5	5	5	6	7	6	5	5	5	6	6	7	5
6	7	5	6	5	4	7	5	5	5	6	5	6	3	5	0	5	5	7	6	6	5	7	5	5	6	4	5	6	6	5	5	6	5	4	6	3	6	3	6	5	6
4	3	6	6	5	3	4	5	6	6	5	5	3	4	5	5	0	6	5	4	4	7	3	7	7	3	6	6	7	6	4	6	6	5	6	6	6	7	6	7	5	7
5	4	5	4	7	6	6	7	6	5	6	6	4	6	6	5	6	0	7	4	6	3	5	5	6	5	5	5	5	4	5	7	7	5	5	5	4	6	5	6	6	7
6	6	6	5	5	6	3	6	3	6	4	6	5	7	7	7	5	7	0	6	4	7	4	6	4	6	6	5	5	5	7	6	3	6	6	3	6	6	6	5	3	5
4	4	3	6	7	4	6	7	6	6	7	7	6	5	6	6	4	4	6	0	7	6	5	5	5	4	5	6	5	6	6	4	6	5	4	5	5	6	5	7	5	6
6	4	6	3	5	6	3	4	5	4	3	6	4	5	5	6	4	6	4	7	0	6	5	4	5	4	7	7	6	6	4	6	5	4	7	7	5	7	6	6	6	6
6	5	5	5	6	6	6	5	5	5	6	5	5	4	6	5	7	3	7	6	6	0	5	5	5	7	7	6	5	5	4	6	7	6	4	7	5	4	6	4	4	6
5	4	7	5	6	6	4	6	6	6	5	5	3	7	6	7	3	5	4	5	5	5	0	7	6	4	5	4	7	5	4	7	6	7	6	5	6	5	7	4	3	6
6	5	5	5	5	7	4	6	4	5	5	5	6	5	4	5	7	5	6	5	4	5	7	0	4	6	5	5	6	6	6	4	5	6	4	6	5	6	7	7	6	4
7	6	6	4	4	7	6	5	6	3	5	7	6	5	5	5	7	6	4	5	5	5	6	4	0	6	4	6	4	7	5	6	5	5	4	6	5	5	6	3	5	7
4	4	4	5	6	4	6	5	7	6	6	6	5	4	5	6	3	5	6	4	4	7	4	6	6	0	5	6	7	7	4	5	7	4	5	5	3	7	5	6	6	6
5	5	7	6	6	6	7	6	6	4	6	6	6	7	5	4	6	5	6	5	7	7	5	5	4	5	0	3	5	6	5	5	6	5	4	5	5	6	5	4	6	6
6	7	7	6	6	5	5	6	5	6	5	5	6	7	5	5	6	5	5	6	7	6	4	5	6	6	3	0	6	4	7	6	6	6	5	3	5	5	6	4	5	5
5	5	6	6	6	7	7	5	6	3	6	6	6	6	4	6	7	5	5	5	6	5	7	6	4	7	5	6	0	3	6	4	5	6	5	5	6	4	4	5	7	5
5	5	6	6	6	7	5	7	6	6	5	5	4	7	5	6	6	4	5	6	6	5	5	6	7	7	6	4	3	0	7	5	3	7	6	4	6	3	4	6	6	4
5	4	7	4	4	6	5	4	6	3	7	5	5	5	5	5	4	5	7	6	4	4	4	6	5	4	5	7	6	7	0	7	6	4	7	7	6	4	5	4	6	6
4	5	3	7	7	5	7	6	4	6	6	6	7	4	5	5	6	7	6	4	6	6	7	4	6	5	5	6	4	5	7	0	5	6	3	6	5	5	4	7	6	3
5	6	5	5	3	7	3	7	4	6	5	5	5	7	6	6	6	7	3	6	5	7	6	5	5	7	6	6	5	3	6	5	0	6	7	4	7	3	4	6	5	3
6	5	5	3	6	3	6	4	6	4	5	7	7	5	7	5	5	5	6	5	4	6	7	6	5	4	5	6	6	7	4	6	6	0	6	6	5	6	4	4	7	7
4	4	4	7	7	5	7	7	5	6	7	5	6	4	6	4	6	5	6	4	7	4	6	4	4	5	4	5	5	6	7	3	7	6	0	6	4	7	6	6	4	6
6	7	5	6	5	5	4	7	4	6	7	5	7	7	5	6	6	5	3	5	7	7	5	6	6	5	5	3	5	4	7	6	4	6	6	0	4	5	4	6	4	4
7	7	3	6	7	4	7	5	6	6	6	7	7	3	5	3	6	4	6	5	5	5	6	5	5	3	5	5	6	6	6	5	7	5	4	4	0	7	3	6	4	6
6	7	6	5	4	7	6	5	6	5	6	5	6	6	5	6	7	6	6	6	7	4	5	6	5	7	6	5	4	3	4	5	3	6	7	5	7	0	4	3	6	3
6	7	4	5	6	4	7	6	5	5	7	7	7	5	6	3	6	5	6	5	6	6	7	7	6	5	5	6	4	4	5	4	4	4	6	4	3	4	0	6	6	5
7	7	7	4	5	6	7	3	7	4	4	6	6	6	6	6	7	6	5	7	6	4	4	7	3	6	4	4	5	6	4	7	6	4	6	6	6	3	6	0	5	6
6	6	4	7	6	5	4	6	4	7	6	5	6	5	7	5	5	6	3	5	6	4	3	6	5	6	6	5	7	6	6	6	5	7	4	4	4	6	6	5	0	5
4	7	4	7	5	7	5	5	4	7	6	3	7	6	5	6	7	7	5	6	6	6	6	4	7	6	6	5	5	4	6	3	3	7	6	4	6	3	5	6	5	0
