# Supplementary material for: Validation of COI metabarcoding primers for terrestrial arthropods
Source: PeerJ. 2019 Oct 7;7:e7745. doi: 10.7717/peerj.7745 (PMC6786254; doi:10.7717/peerj.7745)

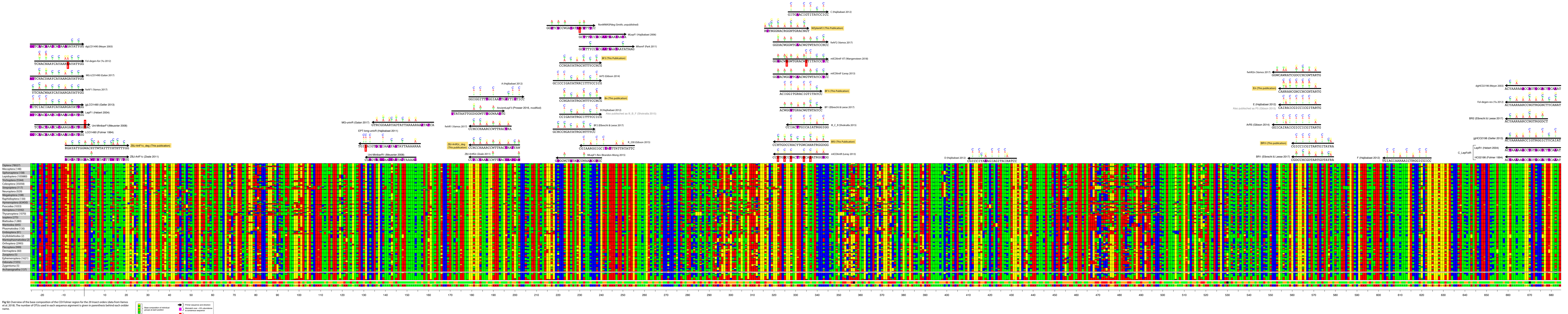


**Fig S2:** Overview of the base composition of the COI Folmer region for the 29 Insect orders (data from Vamos et al. 2018). The number of OTUs used in each sequence aignment is given in parenthesis behind each ordder name.

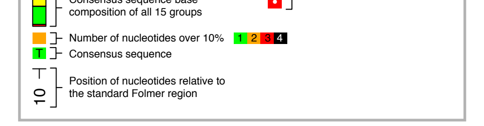

Supplement: Figure S2 [file peerj-07-7745-s002.pdf]
