# Supplementary material for: Validation of COI metabarcoding primers for terrestrial arthropods
Source: PeerJ. 2019 Oct 7;7:e7745. doi: 10.7717/peerj.7745 (PMC6786254; doi:10.7717/peerj.7745)
Supplement: Supplemental Information 1 [file peerj-07-7745-s020.zip › Scripts_1_v2/R_scripts/fusion primers/SXX 21 primer pairs/manual_Levenshtein.pdf]

	1) ZBJ-ArtF1c_GGACAGA	2) fwhF2_GCACTGG	3) LepF1_CGTCGCA	4) LCO1490_AAGTGGG	5) ArF5_TGGTCGC	6) BF1_CTCCGAC	7) mlCOIintF_TGCTTGG	8) mlCOIintF_ATTACAC	) mlCOIintF-XT_CGCTATG	10) LCO1490_ACGGCGG	11) MZplankF2_AATTTAC	12) MhemF_TGAAAGC	13) MLepF1_GAATTGC	14) BF3_CTTCCCC	15) BF2_TCTGCGC	16) Ill_B_F_CTGGACC	17) ArF5_GTCCTGC	8) RonMWASPdeg_GAAGGCG	9) AcientLepF3_AGCTTTT	20) LCO1490_GCCCGCA	21) dgLCO1490_ATTTTGG	1) ZBJ-ArtR2c_CAAACCG	2) fwhR2n_GACATGT	3) MLepF1-Rev_TCTTACG	4) 230_R_ACGTCCT	5) ArR5_GTTCGGT	6) BR2_GCGGAAT	7) jgHCO2198_TACGAAT	Fol-degen-rev_ACAGCTA	9) jgHCO2198_TAAGTTA	10) Ill_C_R_GTGACGG	11) C_LepFolR_CCTCATA	12) dgHCO2198_TGGTTTA	13) C_LepFolR_ATGCGAG	14) BR2_CCACACT	15) BR2_TGCGGTT	16) HCO2198_CTTGGCT	Fol-degen-rev_TAGACTA	18) C_LepFolR_CTGGGTA	19) C_LepFolR_AAGACAT	20) HCO2198_CGCATCT	21) dgHCO2198_TGTAATA
1) ZBJ-ArtF1c_GGACAGA	0	3	4	6	4	5	5	5	5	5	6	3	4	6	6	5	4	5	6	4	6	5	3	6	6	4	5	5	4	5	3	4	5	5	4	5	7	4	6	4	5	4
2) fwhF2_GCACTGG	3	0	6	4	5	5	3	6	4	4	6	5	3	6	5	6	3	4	5	4	4	5	3	5	6	4	5	6	4	5	3	5	6	5	4	5	5	5	6	6	4	5
3) LepF1_CGTCGCA	4	6	0	5	3	4	5	5	5	4	6	5	5	3	4	4	4	5	6	3	6	5	6	5	3	4	5	5	5	6	5	3	5	5	4	5	3	5	4	6	4	4
4) LCO1490_AAGTGGG	6	4	5	0	4	6	4	6	5	3	4	6	4	7	5	6	6	3	5	6	3	5	5	5	4	5	6	6	5	4	4	7	5	3	7	5	5	5	5	4	7	6
5) ArF5_TGGTCGC	4	5	3	4	0	5	3	5	5	4	5	3	4	5	3	3	4	5	5	4	5	6	6	4	4	4	6	6	6	5	4	6	3	5	6	5	5	4	5	5	5	5
6) BF1_CTCCGAC	5	5	4	6	5	0	6	4	5	5	5	5	6	3	3	3	3	6	6	4	6	5	6	4	5	4	5	4	5	6	5	4	7	3	4	5	4	5	4	6	5	6
7) mlCOIintF_TGCTTGG	5	3	5	4	3	6	0	7	3	5	5	4	4	6	4	6	4	5	3	5	3	6	4	3	6	4	6	5	6	5	4	6	3	4	7	4	4	5	5	6	4	5
8) mlCOIintF_ATTACAC	5	6	5	6	5	4	7	0	6	5	3	4	5	3	4	4	5	6	6	6	4	5	6	4	5	5	6	5	5	6	4	5	6	4	4	7	5	5	6	3	6	5
) mlCOIintF-XT_CGCTATG	5	4	5	5	5	5	3	6	0	5	6	5	5	6	5	5	5	6	3	6	5	5	4	4	5	5	4	5	4	6	5	3	4	4	5	4	6	5	5	5	3	4
10) LCO1490_ACGGCGG	5	4	4	3	4	5	5	5	5	0	6	6	6	6	3	5	6	3	6	5	4	5	5	5	3	5	4	5	3	6	3	6	6	3	6	5	5	5	5	4	6	7
11) MZplankF2_AATTTAC	6	6	6	4	5	5	5	3	6	6	0	6	3	5	5	5	5	6	4	7	3	6	5	4	5	6	6	5	4	3	6	5	4	5	6	7	5	5	6	4	6	5
12) MhemF_TGAAAGC	3	5	5	6	3	5	4	4	5	6	6	0	3	6	4	4	5	3	6	5	6	4	4	5	7	6	5	5	5	5	4	6	5	4	5	5	6	5	6	5	5	3
13) MLepF1_GAATTGC	4	3	5	4	4	6	4	5	5	6	3	3	0	6	5	6	3	3	5	5	4	5	3	5	5	5	6	6	6	4	5	7	5	6	6	6	5	5	7	6	5	5
14) BF3_CTTCCCC	6	6	3	7	5	3	6	3	6	6	5	6	6	0	4	3	4	6	6	5	5	4	7	4	4	4	7	6	6	7	5	4	7	5	4	6	3	6	5	6	5	6
15) BF2_TCTGCGC	6	5	4	5	3	3	4	4	5	3	5	4	5	4	0	4	4	5	6	4	5	6	6	3	5	5	5	5	4	5	4	5	6	4	5	5	4	5	4	6	5	5
16) Ill_B_F_CTGGACC	5	6	4	6	3	3	6	4	5	5	5	4	6	3	4	0	5	5	7	5	6	4	7	4	5	6	4	5	6	6	4	5	5	4	4	5	3	4	3	5	4	5
17) ArF5_GTCCTGC	4	3	4	6	4	3	4	5	5	6	5	5	3	4	4	5	0	5	5	3	4	6	3	4	4	3	6	6	6	6	4	5	6	5	6	5	5	6	6	6	5	5
8) RonMWASPdeg_GAAGGCG	5	4	5	3	5	6	5	6	6	3	6	3	3	6	5	5	5	0	7	4	6	3	4	5	5	5	5	5	5	4	4	7	7	4	5	5	4	5	5	4	5	6
9) AcientLepF3_AGCTTTT	6	5	6	5	5	6	3	6	3	6	4	6	5	6	6	7	5	7	0	6	4	7	4	5	4	5	5	5	5	5	7	5	3	5	6	3	5	5	6	5	3	5
20) LCO1490_GCCCGCA	4	4	3	6	4	4	5	6	6	5	7	5	5	5	4	5	3	4	6	0	7	6	5	5	5	4	4	5	4	6	5	4	6	5	4	5	5	6	5	6	5	6
21) dgLCO1490_ATTTTGG	6	4	6	3	5	6	3	4	5	4	3	6	4	5	5	6	4	6	4	7	0	6	5	4	5	4	7	7	6	5	4	6	5	4	7	6	5	6	6	6	6	6
1) ZBJ-ArtR2c_CAAACCG	5	5	5	5	6	5	6	5	5	5	6	4	5	4	6	4	6	3	7	6	6	0	5	4	5	6	6	6	5	5	4	6	7	5	4	7	5	4	6	4	4	6
2) fwhR2n_GACATGT	3	3	6	5	6	6	4	6	4	5	5	4	3	7	6	7	3	4	4	5	5	5	0	6	4	4	5	4	4	5	4	5	6	6	4	5	5	5	6	4	3	5
3) MLepF1-Rev_TCTTACG	6	5	5	5	4	4	3	4	4	5	4	5	5	4	3	4	4	5	5	5	4	4	6	0	4	5	5	5	6	5	4	4	5	5	4	6	4	5	6	6	5	4
4) 230_R_ACGTCCT	6	6	3	4	4	5	6	5	5	3	5	7	5	4	5	5	4	5	4	5	5	5	4	4	0	5	4	4	4	5	5	4	5	5	4	5	4	5	6	3	4	5
5) ArR5_GTTCGGT	4	4	4	5	4	4	4	5	5	5	6	6	5	4	5	6	3	5	5	4	4	6	4	5	5	0	4	4	6	5	3	5	6	4	5	3	3	6	4	6	5	6
6) BR2_GCGGAAT	5	5	5	6	6	5	6	6	4	4	6	5	6	7	5	4	6	5	5	4	7	6	5	5	4	4	0	3	5	6	5	5	5	5	4	3	5	5	5	4	4	4
7) jgHCO2198_TACGAAT	5	6	5	6	6	4	5	5	5	5	5	5	6	6	5	5	6	5	5	5	7	6	4	5	4	4	3	0	5	4	5	5	5	4	4	3	5	3	5	3	5	4
Fol-degen-rev_ACAGCTA	4	4	5	5	6	5	6	5	4	3	4	5	6	6	4	6	6	5	5	4	6	5	4	6	4	6	5	5	0	3	6	4	5	4	4	5	5	3	4	4	4	5
9) jgHCO2198_TAAGTTA	5	5	6	4	5	6	5	6	6	6	3	5	4	7	5	6	6	4	5	6	5	5	5	5	5	5	6	4	3	0	6	5	3	5	6	4	5	3	4	4	6	4
10) Ill_C_R_GTGACGG	3	3	5	4	4	5	4	4	5	3	6	4	5	5	4	4	4	4	7	5	4	4	4	4	5	3	5	5	6	6	0	6	6	3	6	4	5	4	5	4	6	5
11) C_LepFolR_CCTCATA	4	5	3	7	6	4	6	5	3	6	5	6	7	4	5	5	5	7	5	4	6	6	5	4	4	5	5	5	4	5	6	0	5	6	3	6	5	5	4	5	4	3
12) dgHCO2198_TGGTTTA	5	6	5	5	3	7	3	6	4	6	4	5	5	7	6	5	6	7	3	6	5	7	6	5	5	6	5	5	5	3	6	5	0	6	7	3	5	3	3	6	5	3
13) C_LepFolR_ATGCGAG	5	5	5	3	5	3	4	4	4	3	5	4	6	5	4	4	5	4	5	5	4	5	6	5	5	4	5	4	4	5	3	6	6	0	6	4	5	5	4	4	6	5
14) BR2_CCACACT	4	4	4	7	6	4	7	4	5	6	6	5	6	4	5	4	6	5	6	4	7	4	4	4	4	5	4	4	4	6	6	3	7	6	0	6	4	4	6	4	3	6
15) BR2_TGCGGTT	5	5	5	5	5	5	4	7	4	5	7	5	6	6	5	5	5	5	3	5	6	7	5	6	5	3	3	3	5	4	4	6	3	4	6	0	4	5	3	6	4	4
16) HCO2198_CTTGGCT	7	5	3	5	5	4	4	5	6	5	5	6	5	3	4	3	5	4	5	5	5	5	5	4	4	3	5	5	5	5	5	5	5	5	4	4	0	4	3	5	4	6
Fol-degen-rev_TAGACTA	4	5	5	5	4	5	5	5	5	5	5	5	5	6	5	4	6	5	5	6	6	4	5	5	5	6	5	3	3	3	4	5	3	5	4	5	4	0	4	3	5	3
18) C_LepFolR_CTGGGTA	6	6	4	5	5	4	5	6	5	5	6	6	7	5	4	3	6	5	6	5	6	6	6	6	6	4	5	5	4	4	5	4	3	4	6	3	3	4	0	6	5	4
19) C_LepFolR_AAGACAT	4	6	6	4	5	6	6	3	5	4	4	5	6	6	6	5	6	4	5	6	6	4	4	6	3	6	4	3	4	4	4	5	6	4	4	6	5	3	6	0	5	5
20) HCO2198_CGCATCT	5	4	4	7	5	5	4	6	3	6	6	5	5	5	5	4	5	5	3	5	6	4	3	5	4	5	4	5	4	6	6	4	5	6	3	4	4	5	5	5	0	5
21) dgHCO2198_TGTAATA	4	5	4	6	5	6	5	5	4	7	5	3	5	6	5	5	5	6	5	6	6	6	5	4	5	6	4	4	5	4	5	3	3	5	6	4	6	3	4	5	5	0
